# Lipidome Alterations following Mild Traumatic Brain Injury in the Rat

**DOI:** 10.3390/metabo12020150

**Published:** 2022-02-05

**Authors:** Eric C. Gier, Alexis N. Pulliam, David A. Gaul, Samuel G. Moore, Michelle C. LaPlaca, Facundo M. Fernández

**Affiliations:** 1 School of Chemistry and Biochemistry, Georgia Institute of Technology, Atlanta, GA 30332, USA; ericgier4@gmail.com (E.C.G.); david.gaul@chemistry.gatech.edu (D.A.G.); smoore83@gatech.edu (S.G.M.); 2 Wallace H Coulter Department of Biomedical Engineering, Georgia Institute of Technology, Emory University, Atlanta, GA 30332, USA; anpullia3@gatech.edu; 3 Petit Institute for Bioengineering and Bioscience, Georgia Institute of Technology, Atlanta, GA 30332, USA

**Keywords:** mild traumatic brain injury, closed head injury, lipidomics, animal model, ultra-performance liquid chromatography-mass spectrometry

## Abstract

Traumatic brain injury (TBI) poses a major health challenge, with tens of millions of new cases reported globally every year. Brain damage resulting from TBI can vary significantly due to factors including injury severity, injury mechanism and exposure to repeated injury events. Therefore, there is need for robust blood biomarkers. Serum from Sprague Dawley rats was collected at several timepoints within 24 h of mild single or repeat closed head impacts. Serum samples were analyzed via ultra-high-performance liquid chromatography-mass spectrometry (UHPLC-MS) in positive and negative ion modes. Known lipid species were identified through matching to in-house tandem MS databases. Lipid biomarkers have a unique potential to serve as objective molecular measures of injury response as they may be liberated to circulation more readily than larger protein markers. Machine learning and feature selection approaches were used to construct lipid panels capable of distinguishing serum from injured and uninjured rats. The best multivariate lipid panels had over 90% cross-validated sensitivity, selectivity, and accuracy. These mapped onto sphingolipid signaling, autophagy, necroptosis and glycerophospholipid metabolism pathways, with Benjamini adjusted *p*-values less than 0.05. The novel lipid biomarker candidates identified provide insight into the metabolic pathways altered within 24 h of mild TBI.

## 1. Introduction

Traumatic brain injury (TBI) is caused by single or repeated exposure to external forces impacting the skull that result in impaired brain function. The Centers for Disease Control and Prevention’s 2014 surveillance report estimated a total of 2.87 million TBI-related hospitalizations, deaths and emergency department visits in the United States. These estimates represent a 53% increase from studies conducted in 2006, with both studies reporting significant prevalence among children and the elderly [1,2]. When left untreated, TBI events may lead to related neurodegenerative disorders and health consequences, including behavioral impairment [3,4], Alzheimer’s disease [5,6], acquired epilepsy [7,8], and PTSD [9,10]. Currently, methods for diagnosing TBI are crude and do not readily inform about underlying pathological pathways. Diagnosis relies heavily on self-reported symptoms, consciousness assessment through the Glasgow coma scale [11] and brain imaging techniques [12]. These approaches are often not sufficient to capture the diverse physiological and neurochemical processes involved in TBI [13]. The complexity of TBI is highlighted by its vast spectrum of injury severities, modalities, and response mechanisms that make targeted treatment particularly challenging.

The reflection of TBI pathology in biofluids has been shown to depend on severity and injury progression, making the information held within biofluids invaluable [14]. Mild TBI (mTBI) is perhaps the most difficult to correctly diagnose. mTBI is often called “a silent epidemic” as reported incidence rates are likely much lower than the true ones, with many cases misdiagnosed, unreported, or undetected [15]. Approximately 80% of all documented TBI cases are classified as mild and do not present specific, objective clinical symptoms, therefore requiring more advanced diagnostic methods [16]. Biomarkers play a unique role as objective biological signatures capable of accurately differentiating subtle injury states, ultimately contributing more sensitive and specific diagnostic tools for TBI. 

Proteins have received considerable attention as potential TBI biomarkers. For example, the calcium binding protein S100β has been shown to change significantly in serum across a variety of both clinical and pre-clinical TBI studies [17,18]. Recently, the glial and neuronal proteins GFAP and UCH-L1 received FDA approval as part of a blood-based biomarker assay for predicting intracranial hemorrhaging and assessing the need for computed tomography scans in patients with mTBI [19]. While these protein markers have shown promise for mTBI diagnosis, none currently performs well-enough to reliably detect mTBI as a single biomarker [20]. These and other proteins are thought to more readily enter biofluids after severe injury, when the integrity of the blood–brain barrier (BBB) has been compromised [21]. In the event of lower severity injury where the BBB remains largely intact, protein molecules are less likely to enter peripheral biofluid compartments, making them less amenable to non-invasive detection methods. Lipid biomarkers, while less studied than protein counterparts as TBI biomarkers, are attractive candidates for clinical translations given their higher abundance in the brain and ability to more readily penetrate endothelial cells comprising the BBB [22,23]. Current studies into this biomarker class often take a targeted or semi-targeted approach examining a specific group or class of lipid molecules. For example, targeted analysis of sphingomyelins revealed significant increases within 24–48 h of CCI and showed a direct correlation with lesion volume [24,25]. While these lipids are highly specific to the brain and may reveal potential lipid biomarker candidates, untargeted approaches have the potential to reveal a greater breadth of biomarkers that could be even more specific. A recent study by Fiandaca et al. involved an untargeted metabolomics assessment of college athletes and identified a six-lipid biomarker panel capable of accurately classifying mTBI in athletes as early as 6 h post-injury [26]. 

**Figure 1:**
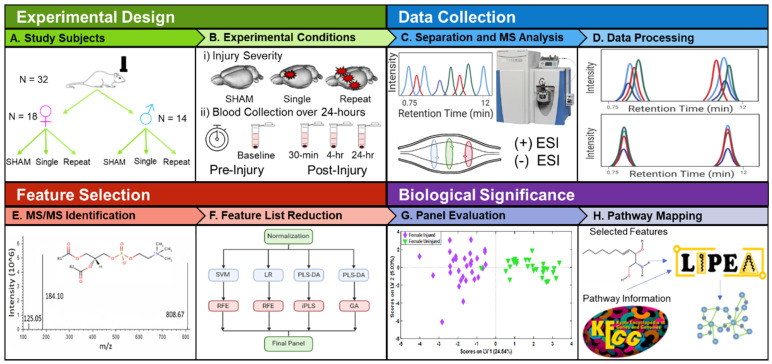
Overview of study design, data processing, feature selection and identification. (**A**) Experimental groups included both male (*n* = 14) and female (*n* = 18) sexes and were assigned to sham controls that received no injuries (*n* = 11), single impact that received one closed head impact (*n* = 10), or repeat impact that received three separate closed head impacts (*n* = 11). (**B**) Injury groups and whole blood collection. (**C**) Workflow illustrating LC-MS data collection in both positive and negative ion modes (**D**) Peak alignment, picking and integration were accomplished using Compound Discoverer v.3.0, a Thermo Scientific software. (**E**) Identification of known lipid species using MSMS spectra collected with data-dependent acquisition (DDA) and in-house databases. (**F**) Multivariate model development and feature selection using machine learning methods to identify features most relevant to differentiating control and TBI classes. (**G**) Features identified by two or more machine learning approaches were combined to create the final oPLS-DA models. (**H**) Features selected in the final panels were imported into LIPEA to determine alignment of lipids with biological pathways altered following TBI.

Previous work by our group utilized a non-targeted approach to identify a panel of 26 lipids capable of differentiating serum of moderate CCI TBI and control Sprague Dawley rat serum samples [27]. High lipidome coverage led to the identification of lipid species optimal for discriminating serum from rodent models at 3- and 7 days post-injury. The objective of the study presented here was to use a similar approach to identify lipid classes and unique lipid features in serum that undergo significant alterations within 24 h of closed head mTBI (Figure 1). A large breadth of lipid metabolites is identified using UHPLC-MS and in-house databases to capture whole class and individual lipid alterations. While the study is limited to lipids contained within databases, the trends identified could serve to guide future avenues of mTBI research toward specific lipid classes, metabolites, and known biological pathways. To our knowledge, this is the first high resolution lipidomics study examining alterations in mTBI across sexes and repeated injury events at multiple timepoints within 24 h of injury.

## 2. Results

**Figure 2:**
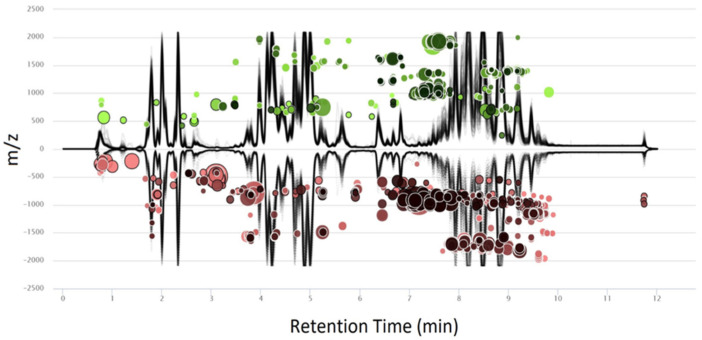
Cloud plot generated in the XCMS web-based application showing positive ion mode retention time versus *m*/*z* of features with high fold change and statistical significance between injured (green) and uninjured (red) models. The black traces outline chromatographic retention time on the *x*-axis and *m*/*z* values on the *y*-axis for each sample. Each bubble in the plot corresponds to one metabolite feature with fold change at or above 1.5 and a *p*-value at or below 0.05 using a Welch’s *t*-test. The color and size of each bubble denote the directionality and magnitude of fold change, respectively, with larger bubbles representing larger fold changes. Darker bubbles correspond to features with greater statistical significance. Features with *m*/*z* values above 2100 are truncated for ease of visibility.

Sera from 32 Sprague Dawley rats were analyzed in positive and negative ion modes to examine lipidome changes resulting from closed-head injury prior to impact (baseline), and at 30 min, 4 h, and 24 h post-injury (Figure 1A,B). All above-background features that met filter criteria (see Section 4) were used to perform unsupervised principal component analysis (PCA) (Figure S1). PCA revealed clear, tight clusters for Sprague Dawley rat reference serum samples and for a pooled quality control (QC), the latter positioned at the center of all study samples. These results indicated that LC-MS experiments were stable and that pooled samples were an accurate representation of the average study sample. Based on initial PCA results, serum from one female rat (ID. No 15) was discarded due to errors during sample preparation, and three additional samples were removed as outliers detected during T^2^ vs. Q residual analysis, reducing the number of serum samples from 114 to 108. Raw data were imported into the XCMS web-based application to visualize metabolites with abundance levels that varied significantly between all injured and uninjured serum samples in positive (Figure 2) and negative (Figure S2) ion modes. Approximately 1500 unique lipids had significantly different abundances between injured and control samples (*p* < 0.05), with fold changes above 1.5. These were projected onto cloud plots as bubbles based on retention time and *m*/*z* [28]. Significantly altered lipids belonged to a wide variety of lipid classes, with approximately 60% of significant features not matched to known compounds in the Metlin database. The diversity of metabolite signatures and the variety of lipid classes with significantly altered species highlight the potential and complexity of the lipidome as a tool for detecting mTBI phenotypes.

**Figure 3:**
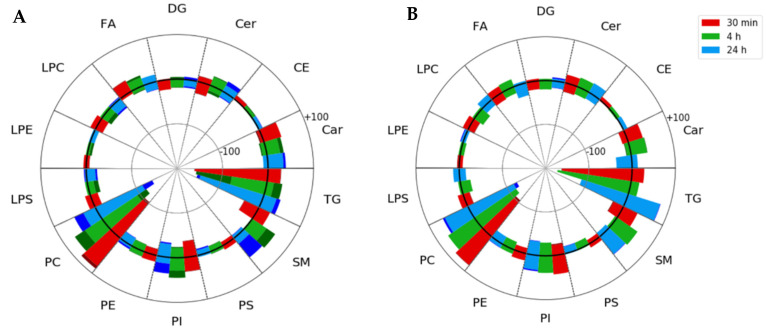
Spider plots of all identified lipids, grouped by lipid classes, shown over the time course of injury progression. Each main lipid class segment contains all three post-injury timepoints, with red, green, and blue bars corresponding to 30 min, 4 and 24 h post-injury timepoints, respectively. Circles within the spider plot correspond to 100 total identified lipids, with the central bold circle corresponding to the zero line. Each post-injury timepoint bar shows the total number of lipids that exhibited either increased median fold change in TBI samples from baseline and fell above the zero line or decreased in TBI samples and fell below the zero line. Darker colors indicate the total number of features with statistically significant changes from baseline after Benjamini–Hochberg correction, and are shown at the tip of each segment. (**A**) Analysis of identified lipids in the repeat (3×) impact injury model and (**B**) analysis of identified lipids in the single (1×) impact injury model. Car—acyl carnitines, CE—cholesteryl esters, Cer—ceramides, DG—diacylglycerols, FA—fatty acids, LPC—lysophosphatidylcholine, LPE—lysophosphatidylethanolamine, LPS—lysophosphatidylserine, PC—phosphatidylcholine, PE—phosphatidylethanolamine, PI—phosphatidylinositol, PS—phosphatidylserine, SM—sphingomyelin, TG—triacylglycerol.

In the interest of obtaining a more comprehensive understanding of lipid pathways being altered in TBI and how these alterations were reflected in the serum lipidome, data dependent acquisition (DDA) LC-MS/MS experiments were performed on both QC and reference serum samples. This led to the tentative identification of 851 species in positive ion mode and 275 species in negative ion mode. Changes in lipid metabolites belonging to the fatty acyl, glycerolipid, glycerophospholipid, and sphingolipid classes were assessed by comparing normalized areas at baseline to each of the post-injury blood collection timepoints (Figure 3). 

The investigation of alterations in lipid classes revealed prominent increases in sphingolipids with upregulation of sphingomyelins (SMs) being the most pronounced. Upregulation of the SM class was observed for both single and repeat injury groups beginning as early as 4 h post-injury. A drastic increase in the number of significantly upregulated SM lipids (q < 0.05) in repeat impact samples over single impact samples was also observed, suggesting that SM abundance increases with repeated injury events. Previous serum studies of single CCI in rodent models support the trend observed in the SM class at both 4- and 24 h post-injury in both serum and plasma [25,29]. Interestingly, Sheth et al. reported that SM concentrations in plasma showed a direct correlation with lesion volume [25]. Glycerophospholipid abundances were largely dependent on the identity of individual lipids, although the general trend in the phosphatidylcholine (PC), phosphatidylethanolamine (PE), and phosphatidylserine (PS) classes showed a decrease over time. This trend appears to be consistent across rodent serum [27,29], brain imaging [24,30], and human plasma studies [31,32] with individual glycerophospholipids varying based on injury severity and time. Glycerolipids, including triacylglycerols (TGs) and diacylglycerols (DGs), showed decreasing trends across acute post-injury timepoints for both repeat and single impacts. However, these trends also appeared to be somewhat specific to individual lipid species. Glycerolipids have received little attention as potential TBI biomarker candidates. Studies using a CCI TBI model showed significant increases in specific DGs in the brain immediately post-injury [33] and in serum at later post-injury timepoints [27].

**Figure 4:**
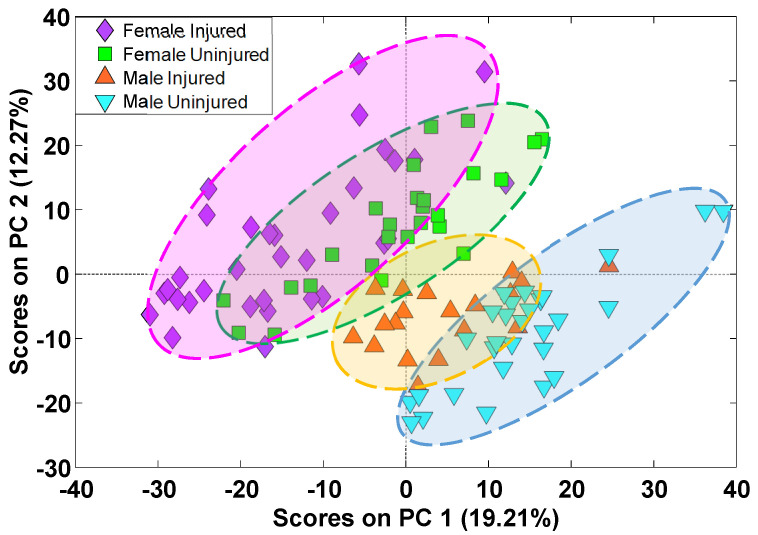
PCA score plots for all identified lipid species. The distribution of samples in principal component space shows separation between male and female animals along the diagonal of PC1 and PC2 with some overlap between injured and uninjured samples.

Alterations in abundance of all annotated lipids was further studied with PCA (Figure 4). Separation of samples from injured and uninjured animals was visible across PC1 and PC2 with significant sexual dimorphism. A similar PCA analysis was carried out only for features with *p*-values at or below 0.05 and having a median fold change at or above 1.5 (Figure S3). In both cases, the separation between injured and uninjured animals was less pronounced than the clustering by sexes. The overlap between uninjured and injured clusters was smaller for the dataset containing only the statistically significant features, as expected. The overlap between injured and uninjured classes and sex differences highlighted the need for supervised analysis for each individual sex to identify groups of lipids most relevant to mTBI.

Recursive feature selection methods were used to reduce the lipid list to those most capable of distinguishing injured and uninjured serum samples, and nested cross-validation was used to optimize parameters and assess initial models for overfitting. Models were generated using support vector machines (SVM), logistic regression, and orthogonalized partial least squares discriminant analysis (oPLS-DA). Supervised classification methods were paired with recursive feature elimination (RFE), genetic algorithms (GA), and inverse partial least squares (iPLS) feature selection methods, independently for male and female animals. The performance of these various classification models and the individual sets of features selected for each model are given in Table 1. Detailed parameters for each of the feature selection methods can be found in the supplementary information (Table S1). The various multivariate models performed with sensitivities between 68.9–94.1%, specificities between 66.4–100% and AUC between 0.75 and 0.94 using between 19 and 32 lipids. 

**Figure 5:**
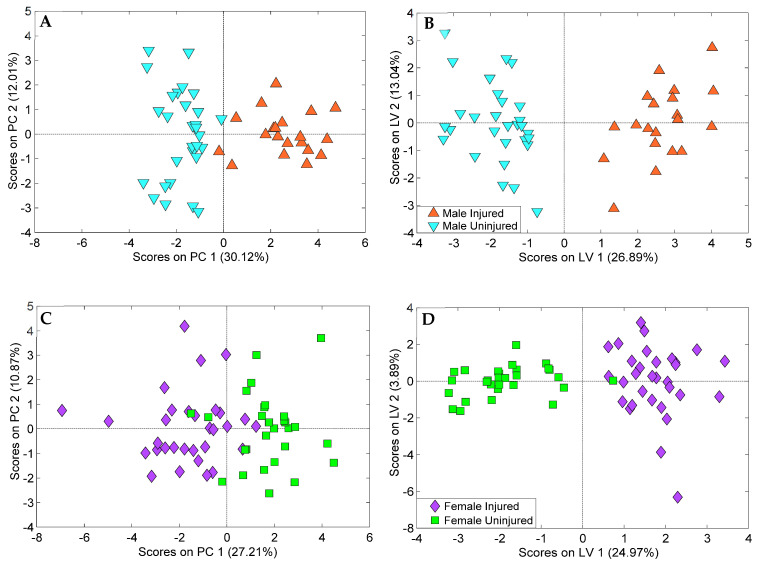
(**A**) PCA and (**B**) oPLS-DA score plot of the 20-lipid panel differentiating sera from injured and uninjured male animals with 2 orthogonal components. (**C**) PCA and (**D**) oPLS-DA score plot of the 19-lipid panel differentiating sera from injured and uninjured female animals with 2 orthogonal components. Both panels were created with 10 iterations of Venetian blinds cross-validation and 200 iterations of permutation testing. Both procedures supported a lack of evidence for overfitting.

Features selected by two or more feature selection methods were used to create a set of final oPLS-DA models containing 20 and 19 features for male and female animals, respectively (Figure 5). These models were built with 2 orthogonal components and performed with over 90% specificity, sensitivity, and accuracy using 10 iterations of Venetian blinds cross-validation. oPLS-DA results showed that injured and uninjured animals were clearly split into two classes along the first latent variable (LV) with injured samples corresponding to positive scores and uninjured samples corresponding to negative scores. Testing for overfitting for these models was performed using PCA analysis and 200 iterations of permutation testing (Figures S4 and S5). PCA analysis also revealed clustering of samples based on blood collection time post-injury. Cross-validation, permutation testing, and PCA all supported a lack of evidence for overfitting.

With an average mass error of <1 ppm for the precursor ions, the elemental formulas and head groups were determined for all lipids, with fatty acyl chain lengths identified whenever possible for all the features in the final marker panels (Table 2a,b). Supplementary Information Table S2 shows the annotation confidence for each of these species and the fragment ion assignments used to determine acyl chain lengths. One of the lipid species (#348) in the final panel for female animals likely corresponded to two possible phosphatidyl choline species that were isobaric and coeluted, making isolation of these species for MS/MS experiments impossible. Distinction of these species would require an additional element of separation such as ion mobility but was beyond the scope of this study. Significance values for each lipid in the panel were calculated using Welch’s *t*-test between baseline measurements and all post-injury timepoints. For brevity, only statistically significant values after Benjamini–Hochberg correction are reported in Table 2, with corresponding blood collection timepoints annotated. For those lipids without any univariately significant timepoints, the lowest *p*-value of the three blood collections timepoints is reported. Features with two or more significant values are denoted with an asterisk.

## 3. Discussion

**Figure 6:**
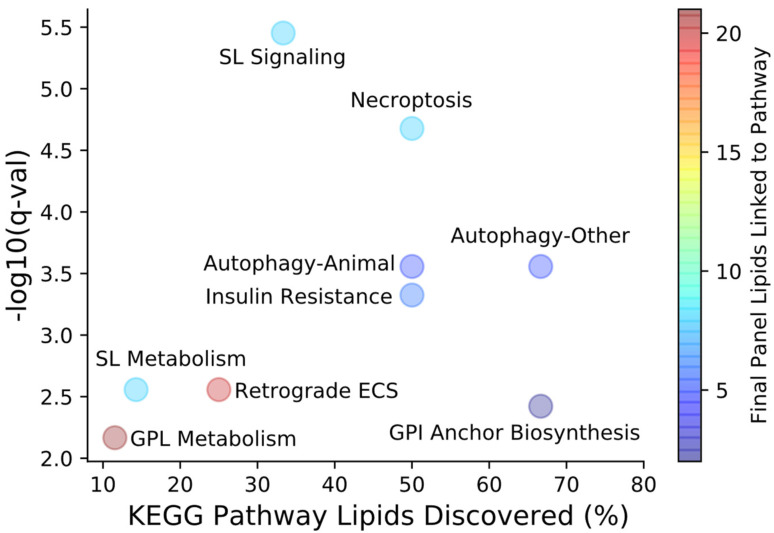
LIPEA pathway analysis for lipids contained within the final feature selection panels. The percentage of all KEGG pathway lipids discovered, shown along the *x*-axis, represents the number of final panel lipids belonging to a specific pathway out of the total number of known pathway lipids. Each of the identified pathways held statistical significance after Benjamini–Hochberg correction (q < 0.10). Colored pathway bubbles denote the total number of lipids in both final panels that can be linked to each pathway. ECS—endocannabinoid signaling, GPL—glycerophospholipid, SL—sphingolipid.

Lipid biomarkers of acute mTBI were identified using an untargeted approach for the optimal selection of features able to differentiate injured and uninjured serum samples across time and injury severity. The early injury timepoints selected in this study correspond to the acute, clinically relevant time periods following single or repeat impacts and may be indicative of early secondary injury cascades. Using high resolution mass spectrometric methods and machine learning approaches coupled with feature selection algorithms, we identified two panels of lipids that consistently separated injured samples from uninjured controls across all measured timepoints of injury progression. Identified features belonged to a variety of classes and showed the myriad of changes that occur in the lipidome following mTBI. Identified features contained within the final feature models were imported into LIPEA using nomenclatures containing the maximum amount of information on acyl chain lengths as possible to link lipids to known biological pathways (Figure 6) [34].

The presence of nine sphingolipid species across both final injury models led to the identification of sphingolipid metabolism and signaling pathways as being significantly altered in our mTBI model. All SM lipids contained in the final panels showed increases in median FC following single and repeat TBI in excess of sham controls beginning at 4 h post-injury with the largest increases consistently exhibited at 24 h in repeat injury models. SM(d16:0/d18:1), a very similar feature to the identified SM(d34:1) contained in the final male panel, and Cer(d18:1) species have been shown to significantly increase at the site of CCI injury as early as 3 and 1 days post-injury, respectively [24]. The changes observed for these lipids in the brain may have been the result of blood located at the site of injury. Follow up work by the same investigators demonstrated the potential of ceramides as biomarkers in regions of the brain separate from the injury site [35]. The primary ceramide synthesis pathways involve acetylation and subsequent reduction in sphinganine or hydrolysis of SMs via acid sphingomyelinases (ASM). Once synthesized, ceramide can be further hydrolyzed by acid ceramidases (AC) to form sphingosine [36]. The alterations in the Cer lipid class followed a similar trend to that of SM, but the percent of statistically significant increased features was lower than for SM. Conversely, the abundance of sphinganine and sphingosine were lower relative to baseline at all post-injury timepoints beginning at 4 h for both single and repeat impacts. Changes in relative abundance of the SM class and sphingosine suggest inhibition of ASM and AC, respectively. The natural inhibition of ASM and AC serves as a defense mechanism for regulating pathways of cell death. Notably, inhibitors of ASM and AC have been suggested as clinical therapies for other brain disorders such as Alzheimer’s disease and major depressive disorder [37,38]. Inhibition of ASM has also been shown to reduce the impairment of neurogenesis and improve behavioral deficits following repetitive mTBI in mice [39]. The findings in this study are perhaps even more promising when considering that that the lipid mass of the human brain is comprised of approximately 15% SM compared to 5% in rats [40], suggesting that the sphingolipids in the panels may be ideal biomarkers for monitoring the rates ASM and AC activity in serum following mTBI.

Autophagy is the process of lysosomal degradation of macromolecular substances that is invariably linked to lipid metabolism through the synthesis and degradation of lipids such as TG and PL [41,42]. Autophagy has been reported in rodent models of fluid percussion and weight drop injury and in humans, primarily through the study of Beclin-1 and LC3-II proteins [43,44,45]. These studies have shown peak expression of Beclin-1 as early as 6 h and increased expression of both Beclin-1 and LC3-II as early as 1-hour post-injury. While the activation of autophagy following TBI is relatively undisputed, whether it is protective or detrimental has yet to be determined [46]. Regulation of autophagy has been proposed as a therapeutic target for TBI as it can provide protection for the compromised BBB and suppress apoptosis, inflammation and oxidative stress [47,48,49]. During autophagy, lipids such as TG, PI and PE are broken down to form free fatty acids and glycerol. Activation of autophagy may therefore partially explain the decreasing trend observed in all identified TG and PE species in the final feature panels as well as the increase in lysoPI(18:0) formed from the breakdown of PI species. Further research on the temporal appearance of these lipids may reveal new findings useful for monitoring autophagy progression following TBI, identifying novel lipid biomarkers to complement protein biomarker candidates.

Most species in the final lipid panels were phospholipid species, leading to LIPEA identification of the glycerophospholipid metabolism pathway. The role of phospholipids in TBI response is widely regarded as complex; numerous studies of the TBI lipidome have revealed lipid fluctuations based on full class and specific lipid molecules [50,51,52]. In both our single and repeat injury models, PC, PE, and PS decreased in abundance over the first 24 h post-injury. Several species-specific trends unique to the injury model were observed for glycerophospholipids, with human studies supporting these trends [53]. Decreases in phospholipid species via the activation of phospholipases likely contributed to the increased formation of free fatty acids such as arachidonic acid and stearic acid, which increased at all post-injury timepoints in our repeat injury model. We previously reported arachidonic acid as a potential biomarker of moderate TBI and differences in abundance in our repeat mTBI model appear to follow a similar increasing trend at acute post-injury timepoints [27]. Interestingly arachidonic acid was selected by the GA feature selection model (feature #161) for our male models but was not selected for the final panel as it was only discovered by a single feature selection method. Biomarker candidates proposed in the Fiandaca et al. study for differentiating injured and uninjured athletes at 6 h post-injury included stearic acid and PE(38:6) and showed trends similar to those in our single and repeat injury models at the nearest timepoint of 4 h [26]. LysoPC(20:4) was also identified in our study, but showed minimal deviation from baseline at 4 h post-injury.

Necroptosis, or programmed necrosis, is a more recently identified form of cellular death occurring in response to stress or glucose/oxygen deprivation that is distinct from apoptotic cell death [54]. Untargeted lipidomic analysis of induced cellular necroptosis has shown significant accumulation of Cer, suggesting that accumulation increases with the progression of necroptosis [55,56]. Interestingly, a similar trend was observed in our study, where the number of ceramides significantly increased from baseline (q < 0.05) grew over the progression of injury (Figure 3A). Of the 40 unique ceramide species identified, 10 showed significant increases 24 h after repeat injury, whereas 2 and 0 ceramide species showed significant increase at 4 h and 30 min post-injury, respectively. The same increase for Cer was not observed in the single impact injury models. While the number of Cer with higher abundance increased over the course of injury progression, no individual species showed statistically significant changes from baseline at any post-injury timepoint. Cer and TG present in the final panels also led to the identification of insulin resistance as a significantly altered pathway following TBI. Studies of single and repetitive TBI have identified brain insulin resistance as a potential therapeutic target and indicator for mortality [57,58]. TG(18:1_18:2_18:2) specifically has been identified as being negatively correlated with homeostatic maintenance for insulin resistance in a variety of lipoproteins and deceases in abundance correspond to increases in brain insulin resistance [59].

There are important limitations to the work presented that should be recognized. First, the markers identified in this study were selected as the optimum panel across all measured acute post-injury timepoints in both single and repeat injury models. Additional markers may present as better candidates at individual timepoints across this study or as markers better suited for either single or repeat impact events. Correlation of these features to histopathological, behavioral, and brain abundance were not measured in this study. Further studies will be needed to link our findings in serum to the broader picture of TBI. Repeated studies using these and other injury models are necessary to verify the accuracy of these markers in serum across independent pre-clinical studies. While these markers were extensively tested for overfitting, further testing in pre-clinical and clinical studies is needed prior to extending these findings to the clinic.

## 4. Materials and Methods

### 4.1. Chemicals

Chemicals used to prepare mobile phases and solutions included LC-MS grade water and acetonitrile (ACN) (Fisher Scientific International, Inc., Pittsburg, PA, USA), isopropyl alcohol (IPA) (Honeywell International, Inc., Charlotte, NC, USA), formic acid (purity > 99.5%) (CovaChem, LLC., Loves Park, IL, USA), and ammonium formate (purity > 99.995%) (Sigma-Aldrich, Inc., St. Louis, MO, USA). Uninjured Sprague Dawley rat serum (ab7488, Abcam, PLC., Cambridge, UK) was used as a reference during LC-MS data collection and to supplement MS/MS data collection. SPLASH II Lipidomix (Sigma-Aldrich, Inc., St. Louis, MO, USA) was used as an analytical internal standard for MS experiments.

### 4.2. Injury Protocol and Blood Collection

All procedures involving Sprague Dawley rat models were performed in accordance with guidelines set forth in the Guide for the Care and Use of Laboratory Animals (U.S. Department of Health and Human Services, Washington, DC, USA, Pub no. 85-23, 1985) and were approved by the Georgia Institute of Technology Institutional Animal Care and Use Committee (protocol #A100188). Female (*n* = 18) and male (*n* = 14) Sprague Dawley rats (8 weeks old; Charles River, Wilmington, MA, USA) weighing between 250–300 g were kept on 12 h reverse light-dark cycles, with food and water available *ad libitum*. Animals were randomly assigned to sham procedure (*n* = 11), single impact (*n* = 10), and three impacts (*n* = 11) groups (Figure 1A). Sham procedure animals received no injuries and were used to ensure that any lipids altered by the administration of anesthesia and handling did not play a role in classification based on injury state.

A CCI pneumatic injury device (Pittsburgh Precision Instruments, Pittsburgh, PA, USA) was modified for use in closed head single and repetitive impacts by adhering a 1 cm silicone stopper (Renovators Supply Manufacturing, Erving, MA, USA) to the standard CCI piston. Prior to injuries, all rat groups were anesthetized with isoflurane (induction: 5% isoflurane; maintenance: 2–3% isoflurane), and the head was placed on a 1-inch thick ethylene-vinyl acetate foam (#86095K46, McMaster-Carr, Elmhurst, IL, USA). Thirty seconds after removal of the isoflurane supply injury was induced to the dorsal surface of the head, centered above the midpoint between bregma and lambda skull suture landmarks at a velocity of 5 m/s. The single impact injury group received one impact with a 5 mm head displacement. The repeat injury group received a total of 3 impacts at 2 min intervals, with head displacements of 5 mm, 2 mm, and 2 mm, respectively. Sham procedure animals were subjected to all procedures as injured animals excluding impact(s) (Figure 1B). Following the final impact, righting time was recorded, and animals were monitored until mobile and exhibiting normal behaviors. Righting time was observed to be significantly different between sham and repeat impact animals but not significantly different between sham and single impacts (Table S3, Figure S6). Animals were returned to home cages and singly housed with soft bedding during recovery. Animals were monitored after injury and returned to home cages with respective cage mates until sacrifice. At each collection time (baseline/pre-injury, 30 min, 4 h, 24 h), rats were anesthetized with isoflurane (5% induction, 2–3% maintenance) and approximately 200 µL of whole blood was collected from a tail artery punctured by 20-gauge Precision Glide needles and stored on ice. Blood was collected from the gingival vein (*n* = 5) when the tail artery was not assessable. Whole blood samples were allowed to coagulate at room temperature for 45 min. Samples were then centrifuged at 4 °C for 15 min at 2500× *g*, and serum was separated into 50 μL aliquots and stored at −80 °C. The blood collection protocol was conducted in accordance with previously published methods [27]. Timepoints for blood collection were determined based on BBB permeability studies that demonstrate BBB permeability as early as 30 min and peaking between 4–6 h post-injury [60,61].

### 4.3. Sample Preparation and Ultrahigh Performance Liquid Chromatography-Mass Spectrometry (UHPLC-MS) Analysis

A standard spiked IPA solution was prepared with 250 µL of SPLASH II Lipidomix and 14.750 mL of IPA. Serum samples were thawed on ice for 1 h prior to the addition of the IPA solution in a 1:3 *v*/*v* ratio to separate lipids and small non-polar metabolites from proteins. Mixtures of serum and IPA solution were vortexed for 10 s and centrifuged at 16,000× *g* for 7 min. The supernatant was then collected for LC-MS analysis. Sample blanks were prepared with 50 µL of LC-MS grade water, and pooled QC samples were prepared from 5 µL aliquots of all study subject serum samples. Serum reference samples from uninjured Sprague Dawley rat serum were processed in the same manner as study subject serum samples. All samples were run in a randomized order over 2.5 days of consecutive instrument time. QC samples were interleaved every 24 runs to evaluate LC-MS system stability and to account for time-dependent batch effects.

Reverse phase (RP) chromatography was preformed using a Vanquish Horizon UHPLC (Thermo Fisher Scientific, Inc., Waltham, MA, USA) instrument. Mobile phase A was a (40:60 *v*/*v*) water/ACN mixture and mobile phase B was a (90:10 *v*/*v*) IPA/ACN mixture. Both mobile phases contained 0.1% formic acid and 10 mM ammonium formate solution (Table S4). The stationary phase used was a 2.1 × 50 mm Accucore C30 column with 2.1 µm particle size. Analysis with an ID-X Orbitrap Tribrid mass spectrometer (Thermo Fisher Scientific, Inc., Waltham, MA, USA) in both positive and negative ion modes was used following LC separation for all samples. The scan range was 150–2000 *m*/*z* (Figure 1C). Data dependent acquisition (DDA) experiments were performed in positive and negative modes on QC and reference serum samples. In total four DDA experiments were performed. All experiments included the use of an exclusion list and two, one in each ionization mode, employed an inclusion list [62]. Details for LC-MS and DDA-MS/MS methods are given in Table S4. 

### 4.4. Data Processing

Raw spectral data from LC-MS experiments were processed using Compound Discoverer v3.0.0 software (Thermo Fischer Scientific, Inc., Waltham, MA, USA) and the XCMS web-based application (xcmsonline.scripps.edu (accessed on 25 October 2020)). Initial steps involved retention time alignment between samples, peak area integration, peak picking, and QC area normalization (Figure 1D). Features eluting with the solvent front or having retention times below 0.75 min were removed to account for potential ion suppression effects in that retention time region [63]. Further filtering was applied to remove features not present in at least 75% of all samples at concentrations 5 times above the baseline abundance, and features with coefficients of variation (CV_m_) greater than 20% in QC samples. A combined set of 14,119 spectral features, 3646 and 10,473 from the negative and positive ion modes, respectively, was obtained.

ChemSpider and in-house mzVault database searches were used to obtain a list of tentative IDs based on accurate mass, isotope pattern, and MS/MS data whenever possible (Figure 1E). Each lipid feature was identified according to the following confidence levels: (1) compounds matched to existing in house database standards by accurate mass (<2 ppm), isotopic abundance, fragmentation spectrum, and retention time; (2) compounds annotated according to accurate mass, isotopic abundance, and fragmentation consistent with Lipid Maps and Human Metabolome Database (HMDB) entries; (3) accurate mass match matched to Lipid Maps and HMDB entries and fragmentation showing a few matching characteristic fragment ions [64,65,66]. Feature identification led to a panel of 1126 annotated lipid species (Table S5). These features were imported as a single matrix into MATLAB (MATLAB R2019a, The Mathworks, Natick, MA, USA, with PLS Toolbox v8.1.1, Eigenvector Research Inc., Wenatchee, WA, USA) and Python (Python Software Foundation, Beaverton, OR, USA, with Scikit-learn v0.24.2) [67] for further uni- and multi-variate analysis.

### 4.5. Feature Selection and Pathway Mapping

Features were preprocessed using autoscaling prior to binary classification. Serum samples were separated based on sex to avoid confounding effects. Sham and samples collected prior to injury (baseline samples) were collectively grouped in a single non-injured class while all serum samples collected post-injury from single and repeat impact animals were grouped into a single injured class. This was done to increase the total number of samples in each class, prevent overfitting of the data, and increase statistical significance of the models. Predictive models were created using four different combinations of classifiers and feature selection methods. Models were optimized using 10-fold nested cross-validation. Analysis was performed separately on both male and female animals. Prediction models included SVM, logistic regression, and oPLS-DA. Lipids that distinguished uninjured from injured animals were selected using RFE, GA and iPLS [68,69,70]. Cost function optimization for SVM and logistic regression models was preformed using a grid search and nested cross-validation within Python prior to RFE feature selection. Detailed settings for prediction models and feature selection methods can be found in the supporting information (Table S1). Lipid features selected by two or more feature selection methods were used to create final oPLS-DA models for classifying injured and uninjured serum samples. Reported *p*-values are from Welch’s *t*-test following Benjamini–Hochberg adjustment [71]. Median fold changes were calculated comparing injured serum to corresponding baseline measurements. Further evaluation to support a lack of evidence for overfitting included 200 iterations of permutation testing for both final optimized panels (Figure S5) and PCA comparison of clusters produced by the 1116-feature set and the final models [72].

Known feature ID of the optimized lipid panels were imported into the lipid pathway enrichment analysis (LIPEA) web tool using an abbreviated lipid format and the Rattus Norvegicus organism background [34]. Pathways identified as possessing significant association with the potential biomarker panels discovered were those with Benjamini–Hochberg corrected q-values < 0.05. Data and code for SVM and LR models generated through this work are available through the NIH Metabolomics Workbench under the study ID ST001950 (http://dx.doi.org/10.21228/M8TB0S (accessed on 24 October 2021)). 

## 5. Conclusions

The results described in this manuscript illustrate the potential of lipids as serum biomarkers for TBI across a range of variables, including acute post-injury timepoints, sex, and injury severity. Despite the demonstrated success in predicting the presence of TBI in animals, additional work is required to test the robustness of the proposed biomarker candidates, as well as to evaluate whether these markers are specific to brain injury or if they are reflective of systemic inflammation processes or other changes in generic damage-associated molecular pathways. Lipid species not covered in this study, such as glycosphingolipids and oxidized lipids, may also be useful lipid biomarker candidates in addition to those presented here. Additional post-injury subacute and chronic timepoints would help determine the optimal biomarker sampling time and to gauge injury progression and recovery trajectories. While it is proposed that small non-polar lipid molecules can permeate the BBB, this has only been definitively shown in higher severity injury models. While this is likely true to a similar or lesser extent for lower severity injuries, further research is still required to validate this claim. Measuring alterations of the proposed serum biomarkers in brain tissue directly would also be important to further understand the mechanisms involved in TBI pathophysiology. Additionally, investigation of the spatial distribution of the lipid features identified in this work and others using mass spectrometry imaging techniques would enhance the qualitative and quantitative understanding of lipid-related TBI pathogenesis directly at the site of injury and in surrounding brain regions.

## Figures and Tables

**Table 1:** Classifier performance and selected features used to build models for distinguishing serum-derived lipids from injured and uninjured animals. Classifier and feature selection pairs were run independently for male and female animals. Cross-validation estimates were calculated using the average area under the curve (AUC) of five random subsets and served to evaluate performance of each model on previously unseen data. When models were trained on the full dataset (all samples), AUC estimates approached unity. Features selected by two or more feature selection methods are shown in bold and were used to create the final classification models.

Classifier	Feature Selection Method	Sex	Number of Features	Cross-Validation Estimate, AUC (SD)	All Samples, AUC	Selected Features
Linear SVM	RFE	M	27	0.875 (0.133)	0.980	**63**, **89**, 244, **258**, **365**, 378, 417, **453**, 457, **459**, **476**, **497**, **527**, **541**, 543, **551**, **570**, 635, **651**, **788**, **792**, 798, **808**, 857, **967**, **1095**, **1114**,
Logistic Regression	RFE	M	24	0.840 (0.174)	0.992	88, **89**, 183, 279, **365**, **453**, 457, **459**, 473, **476**, 486, 502, **527**, **543**, **551**, **570**, **601**, **651**, **652**, **788**, **792**, **808**, 1104, **1114**
oPLS-DA	GA	M	31	0.941 (0.062)	1.000	17, **63**, 161, 171, 174, 209, 278, 316, **365**, 407, 494, **497**, 513, **527**, 531, **543**, 550, **551**, 567, 589, **601**, 616, 621, 626, 627, **652**, 745, 774, **788**, 1080, **1114**
oPLS-DA	iPLS	M	20	0.891 (0.090)	0.992	61, 101, **258**, 273, 321, 346, **365**, 473, **527**, **543**, **570**, 617, **652**, 851, 876, 951, 994, 998, 1008, **1095**
Linear SVM	RFE	F	28	0.766 (0.140)	0.953	**8**, 10, **35**, **103**, 104, **282**, **328**, **346**, **348**, 349, **388**, **437**, 457, 460, 490, 615, **757**, 780, 784, **813**, **825**, **874**, **875**, 920, **989**, 1026, 1044, **1110**
Logistic Regression	RFE	F	29	0.752 (0.120)	0.976	**8**, **35**, 73, 81, 86, **103**, 263, **282**, **328**, **346**, **348**, **388**, 417, **437**, 443, **455**, 532, **620**, 745, **757**, **813**, **825**, **874**, 875, 972, 988, **989**, 1055, **1110**
oPLS-DA	GA	F	29	0.949 (0.156)	0.993	**8**, **27**, **103**, 154, 270, 378, 387, 408, 416, **455**, 477, 531, 538, 550, **620**, 647, 648, 652, 669, 712, 717, 719, 774, **825**, 854, 869, 1082, 1095, **1110**
oPLS-DA	iPLS	F	24	0.880 (0.110)	0.943	**27**, 34, 141, 146, 149, 153, 299, **328**, 381, 410, 425, 529, 590, **620**, 621, 634, 675, 714, 751, 773, 842, 903, 936, **989**

**Table 2:** **a**: Annotation of the final 20-lipid panel in male rats. **b**: Annotation of final 19-lipid panel in female rats. Retention time, observed exact mass with instrumental error, observed electrospray adduct, predicted elemental formula, significant *p*-values for timepoints between repeat TBI and baseline, and fold change (FC) are reported. Positive FC values correspond to increased abundance in serum from injured animals vs. baseline samples, and negative FC values correspond to decreased abundance in injured animals. Fatty acid chain information is provided based on MS/MS information. Detailed MS/MS fragmentation information is provided in the supporting information (Table S2a,b).

Feature Number	Retention Time (min)	*m*/*z* Mass Error (ppm)	Detected Ion	Elemental Formula	Annotation	*p*-Value (TBI vs. Baseline)	Fold Change	Time
**a**								
63	8.893	716.6343 −0.253	[M+NH_4_]^+^	C_49_H_78_O_2_	CE(22:5)	0.0655	1.340	4 h
89	7.303	652.6605 −0.329	[M+H]^+^	C_42_H_85_NO_3_	Cer(d18:0/24:0)	0.0292	−1.553	4 h
258	2.186	601.3349 −0.518	[M+H]^+^	C_27_H_53_O_12_P	LysoPI(18:0)	0.0147	1.190	24 h
365	5.442	800.6168 −0.096	[M+H]^+^	C_45_H_86_NO_8_P	PC(18:2_19:0)	0.245	−1.042	30 min
453	4.982	880.6071 0.446	[M+HCO_2_]^−^	C_48_H_86_NO_8_P	PC(18:0_22:5)	0.0483	1.207	4 h *
459	4.742	878.5919 0.958	[M+HCO_2_]^−^	C_48_H_84_NO_8_P	PC(18:0_22:6)	0.0198	1.192	4 h
476	4.337	846.6008 −0.494	[M+H]^+^	C_49_H_84_NO_8_P	PC(41:7)	0.0323	1.425	4 h
497	4.127	858.6014 0.153	[M+H]^+^	C_50_H_84_NO_8_P	PC(42:8)	0.249	1.070	4 h
527	4.773	718.5752 0.230	[M+H]^+^	C_40_H_80_NO_7_P	PC(O-16:1/16:0)	0.146	−1.603	4 h
543	4.326	816.5910 0.387	[M+H]^+^	C_48_H_82_NO_7_P	PC(O-18:2_22:6)	0.0375	1.493	30 min *
551	5.557	772.6218 −0.197	[M+H]^+^	C_44_H_86_NO_7_P	PC(O-18:1/18:1)	0.277	1.035	30 min
570	5.768	798.6379 0.386	[M+H]^+^	C_46_H_88_NO_7_P	PC(O-38:3)	0.221	1.054	24 h
601	4.317	818.6062 −0.162	[M+H]^+^	C_48_H_84_NO_7_P	PC(O-18:1/22:6)	0.00800	1.607	4 h **
651	5.588	704.5591 −0.433	[M+H]^+^	C_39_H_78_NO_7_P	PE(O-34:1)	0.0156	−1.605	4 h *
652	6.297	704.5590 −0.504	[M+H]^+^	C_39_H_78_NO_7_P	PE(O-18:1/16:0)	0.0144	−1.656	4 h *
788	3.676	689.5595 −0.217	[M+H]^+^	C_38_H_77_N_2_O_6_P	SM(d33:1)	0.0953	1.153	24 h
792	3.978	703.5759 0.797	[M+H]^+^	C_39_H_79_N_2_O_6_P	SM(d34:1)	0.0335	1.296	24 h
808	3.613	727.5758 0.606	[M+H]^+^	C_41_H_79_N_2_O_6_P	SM(d36:3)	0.000266	1.632	4 h *
1095	8.758	984.8954 −0.423	[M+NH_4_]^+^	C_63_H_114_O_6_	TG(60:4)	0.221	−1.648	24 h
1114	9.754	1014.9420 −0.782	[M+NH_4_]^+^	C_65_H_120_O_6_	TG(18:1_20:1_24:1)	0.0275	−1.446	4 h
**b**								
8	0.811	246.1701 0.284	[M+H]^+^	C_12_H_23_NO_4_	Car(5:0)	0.0137	−1.374	30 min
27	1.325	414.3215 0.313	[M+H]^+^	C_23_H_43_NO_5_	Car(16:1-OH)	0.0932	1.660	4 h
35	1.701	442.3528 0.316	[M+H]^+^	C_25_H_47_NO_5_	Car(18:1-OH)	0.00239	2.019	4 h
103	7.349	708.6512 0.868	[M+HCO_2_]^−^	C_43_H_85_NO_3_	Cer(d18:1/25:0)	0.182	1.260	4 h
282	3.997	718.5386 0.654	[M+H]^+^	C_39_H_76_NO_8_P	PE(16:0_18:1)	0.00113	−2.354	4 h **
328	4.598	772.5858 0.286	[M+H]^+^	C_43_H_82_NO_8_P	PC(17:0_18:2)	0.156	−1.407	24 h
346	4.434	784.5856 −0.036	[M+H]^+^	C_44_H_82_NO_8_P	PC(18:1_18:2)	0.0471	−1.381	24 h
348	4.471	828.5764 −1.111	[M+HCO_2_]^−^	C_44_H_82_NO_8_P	PC(16:0_20:3)	0.215	1.183	24 h
388	5.752	814.6323 −0.277	[M+H]^+^	C_46_H_88_NO_8_P	PC(18:0_20:2)	0.113	1.077	24 h
437	4.243	864.5763 0.996	[M+HCO_2_]^−^	C_47_H_82_NO_8_P	PC(17:0_22:6)	0.0510	1.112	24 h
455	4.646	880.6078 1.206	[M+HCO_2_]^−^	C_48_H_86_NO_8_P	PC(18:0_22:5)	0.125	1.382	30 min
620	4.510	742.5392 0.712	[M+H]^+^	C_41_H_76_NO_8_P	PE(18:1_18:2)	0.00600	−2.169	24 h **
757	1.899	838.5572 −0.148	[M+Na]^+^	C_44_H_82_NO_10_P	PS(38:2)	0.150	−1.357	24 h
813	5.578	759.6379 −0.112	[M+H]^+^	C_43_H_87_N_2_O_6_P	SM(d16:0_ 22:1)	0.00597	1.456	4 h *
825	5.316	771.6381 −0.113	[M+H]^+^	C_44_H_87_N_2_O_6_P	SM(d39:2)	0.0143	1.619	24 h
874	1.840	302.3054 −1.448	[M+H]^+^	C_18_H_39_NO_2_	Sphinganine (C18)	0.131	−1.281	4 h
875	1.699	300.2898 0.299	[M+H]^+^	C_18_H_37_NO_2_	Sphingosine (C18)	0.205	−1.508	24 h
989	8.176	898.7861 −0.280	[M+NH_4_]^+^	C_57_H_100_O_6_	TG(18:1_18:2_18:2)	0.0128	−2.530	4 h
1110	8.837	998.9114 −0.076	[M+NH_4_]^+^	C_64_H_116_O_6_	TG(61:4)	0.000911	−2.341	4 h *

* Feature held statistical significance at one other timepoint under repeat TBI vs. baseline comparison; ** Feature held statistical significance at all timepoints under repeat TBI vs. baseline comparison.

## Data Availability

Data and code for SVM and LR models generated through this work are available through the NIH Metabolomics Workbench under study ID ST001950 (http://dx.doi.org/10.21228/M8TB0S (accessed on 24 October 2021)). Raw data are provided in mzML format, data processed with Compound Discoverer is available in an excel file and machine learning code is compiled with Juptyer notebooks.

## Supplementary Materials

The following supporting information can be downloaded at: https://www.mdpi.com/article/10.3390/metabo12020150/s1, Figure S1: PCA scores plot for the complete LC-MS dataset, Figure S2: Negative ion mode XCMS cloud plot, Figure S3: PCA of statistically significant features, Figure S4: PCA using reduced lipid panels for samples as a function of time, Figure S5: Permutation testing for PLS-DA models with selected features, Figure S6: Acute neurological assessment of righting reflex time, Table S1: Parameters used for model generation and feature selection, Table S2: MS/MS of selected lipids, Table S3: Summary of characteristics of animal cohort and experimental design, Table S4: LC-MS method parameters, Table S5: Table of identified lipids.
