# Supplementary material for: Lipidome Alterations following Mild Traumatic Brain Injury in the Rat
Source: Metabolites. 2022 Feb 5;12(2):150. doi: 10.3390/metabo12020150 (PMC8878543; doi:10.3390/metabo12020150)
Supplement: Supplementary file 1 [file metabolites-12-00150-s001.zip › mTBI Metabolites_SI_122621.pdf]

Supporting information for

Lipidome Alterations Following Mild Traumatic Brain Injury.

Eric C. Gier[1], Alexis N. Pulliam[2], David A. Gaul[1,3], Samuel G. Moore[1,3], Michelle C. LaPlaca[2,3] *, and Facundo M. Fernández[1,3] *

[1]School of Chemistry and Biochemistry, Georgia Institute of Technology, Atlanta, Georgia 30332, United States.

[2]Wallace H Coulter Department of Biomedical Engineering, Georgia Institute of Technology, Atlanta, Georgia 30332, United States.

[3]Petit Institute for Bioengineering and Bioscience, Georgia Institute of Technology, Atlanta, Georgia 30332, United States.

*Correspondence: facundo.fernandez@chemistry.gatech.edu (F.M.F.); Tel.: +1-404-385-4432 (F.M.F.); michelle.laplaca@bme.gatech.edu (M.C.L.); Tel.: +1-404-385-5044 (M.C.L.)

Table of Contents

Figure S1. PCA scores plot for the complete LC-MS dataset.

Figure S2. Negative ion mode XCMS cloud plot.

Figure S3. PCA of statistically significant features.

Figure S4. PCA using reduced lipid panels for samples as a function of time.

Figure S5. Permutation testing for PLS-DA models with selected features.

Figure S6. Acute neurological assessment of righting reflex time.

Table S1. Parameters used for model generation and feature selection.

Table S2. MS/MS of selected lipids.

Table S3. Summary of the characteristics of the animal cohort and experimental design.

Table S4. LC-MS method parameters.

Table S5. Table of identified lipids.

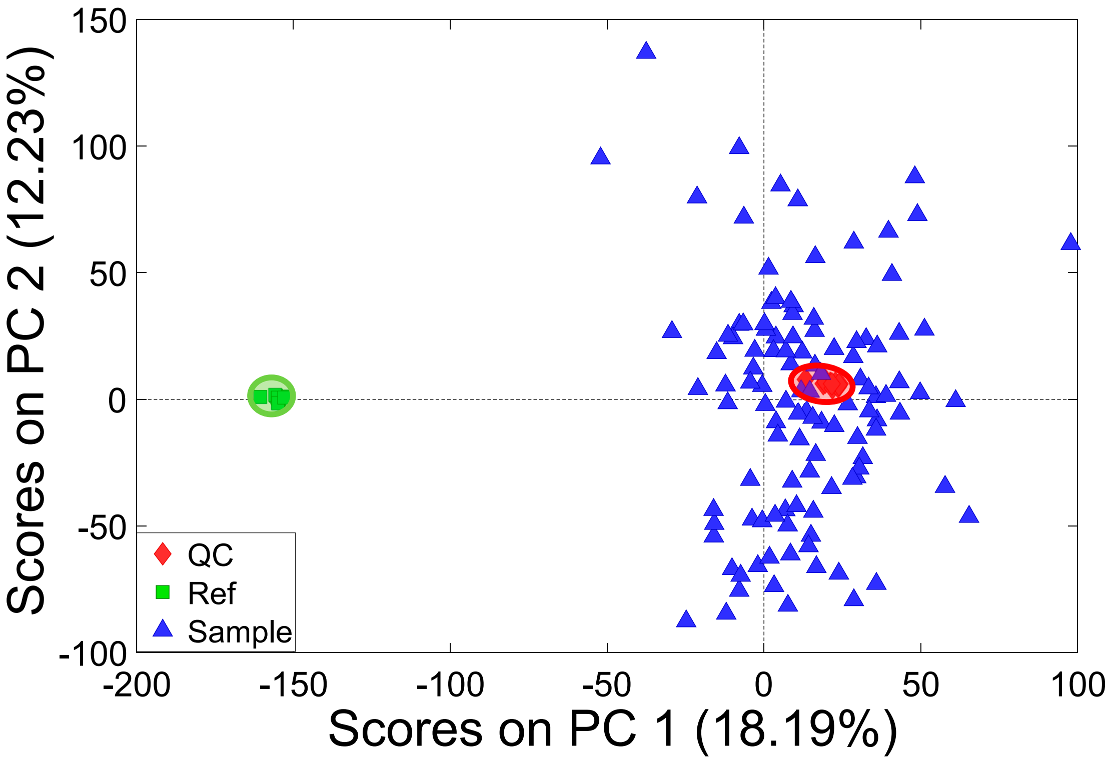

Supplementary Figure S1: Principal Component Analysis (PCA) scores plot of LC-MS data for quality control (QC), uninjured reference Sprague-Dawley rat serum (Ref), and Sprague-Dawley rat serum from all study subjects. A combined 14,119 features were detected above background levels after initial data processing in the positive and negative ion modes. QC samples showed good clustering in the center of all study samples, indicating that the collected LC-MS data had excellent stability over the entire lipidomic analysis.

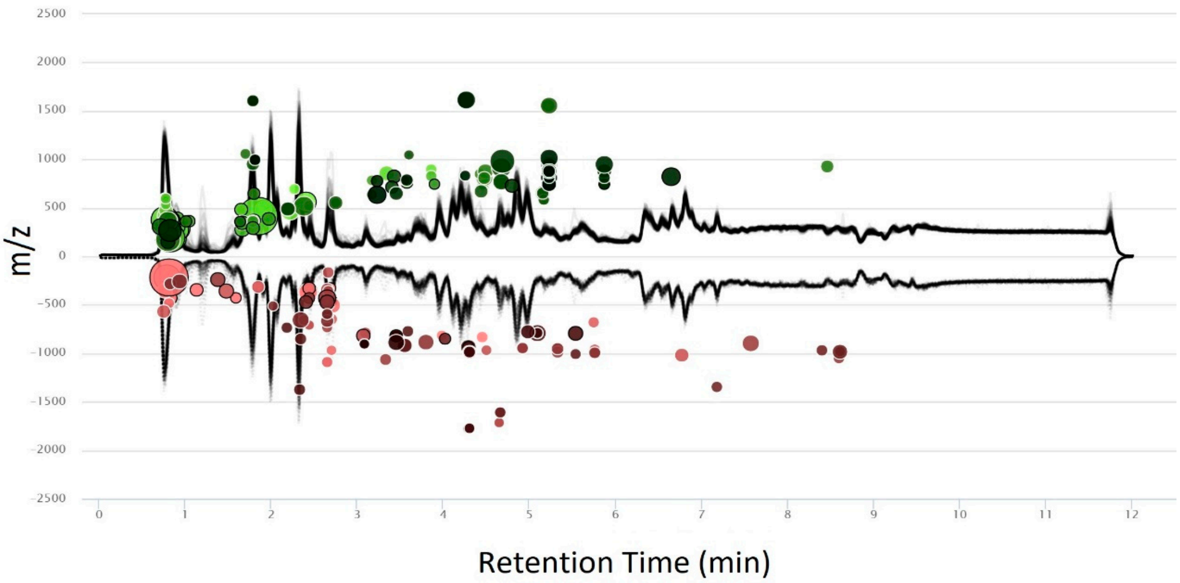

Supplementary Figure S2: XCMS cloud plot showing retention time versus *m/z* for negative ion mode features with high fold changes and statistical significance between injured (green) and uninjured (red) animals. The black traces show the chromatographic retention time on the x-axis. The LC-MS features' *m/z* values are shown on the y-axis. Each bubble in the plot corresponds to a lipid feature with a fold change at or above 1.5 and a *p*-value at or below 0.05 using Welch's t-test. The color and size of each bubble denote the directionality and magnitude of fold change, respectively, with larger bubbles representing larger fold changes. Darker bubbles correspond to features with greater statistical significance. Features with *m/z* values above 2100 were truncated for clarity purposes.

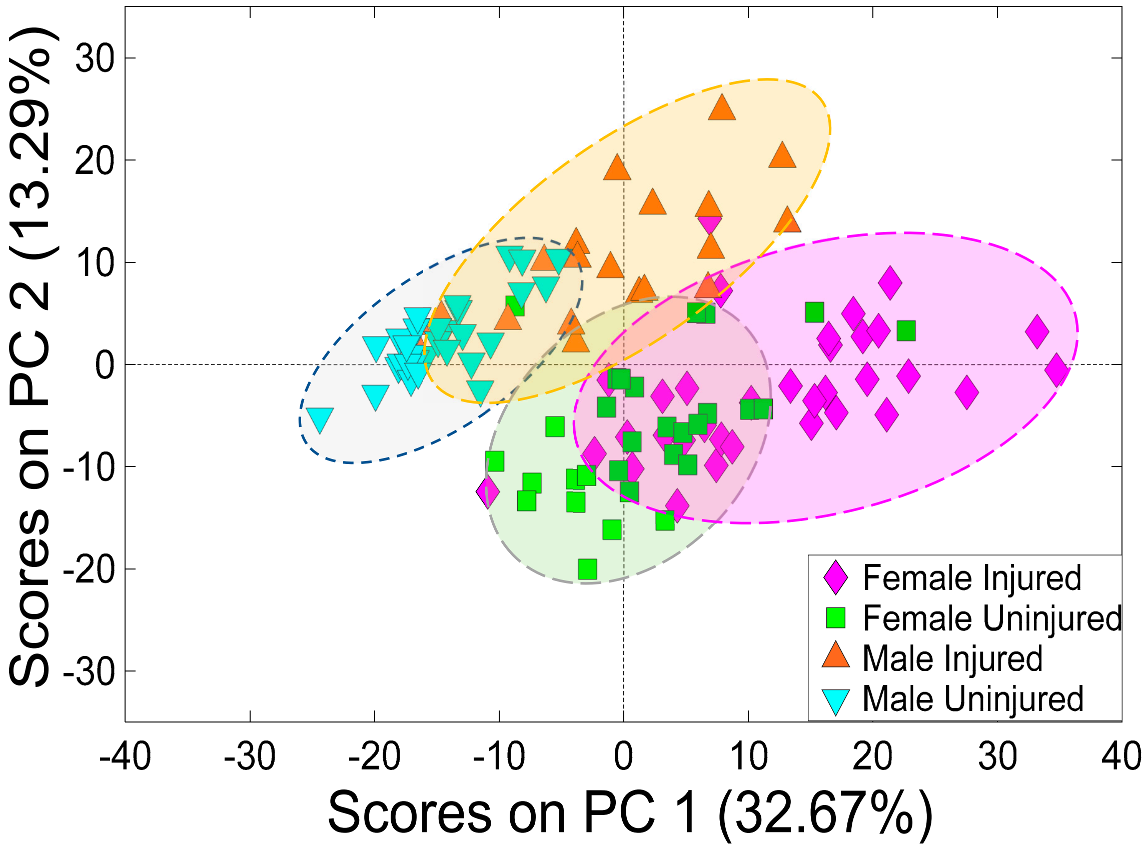

Supplementary Figure S3: PCA scores plot for features with p-values at or below 0.05 and having a median fold change at or above 1.5 between injured and uninjured animals. The distribution of samples shows separation between male and female sexes along the diagonal of PC1 and PC2. Separation of injured and uninjured serum is subtler but still significant, with some overlap indicating the need for supervised classification methods.

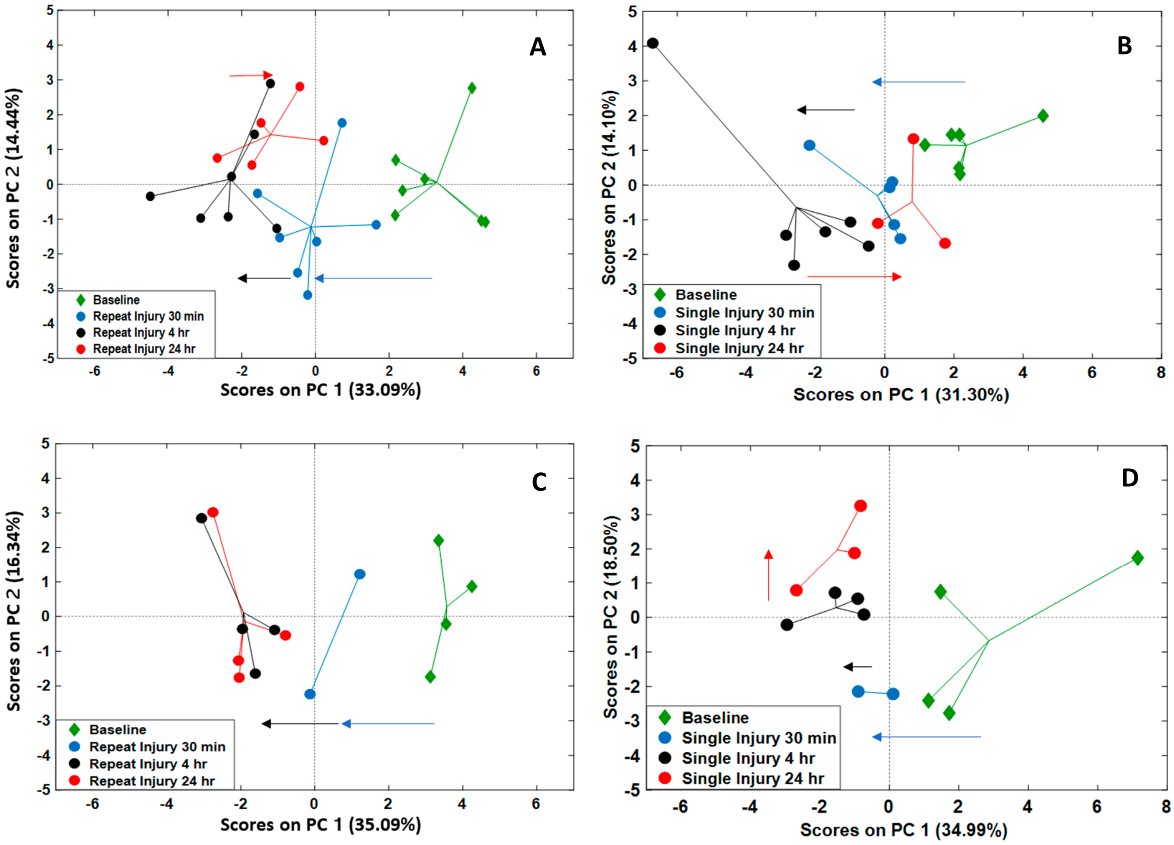

Supplementary Figure S4: PCA score plots using the reduced lipid panels for female (A-B) and male (C-D) animal models. Samples in the score plots contain either repeat impact sera (A and C) or single impact sera (B and D). Arrows are drawn from the centroid of the previous blood collection time point to the centroid of the next blood collection timepoint in the study to highlight the trends between sequential blood collections. Injury samples are primarily separated along the first principal component with uninjured baseline samples generally corresponding to positive scores on PC1 and injured samples corresponding to negative scores on PC1. Female sera collected at 24 h from single impact injury models (B) grouped closer to baseline samples than any other blood collection timepoint across the injury models studied, indicating that the lipids selected as TBI markers returned close to pre-injury levels in the lower severity female injury model.

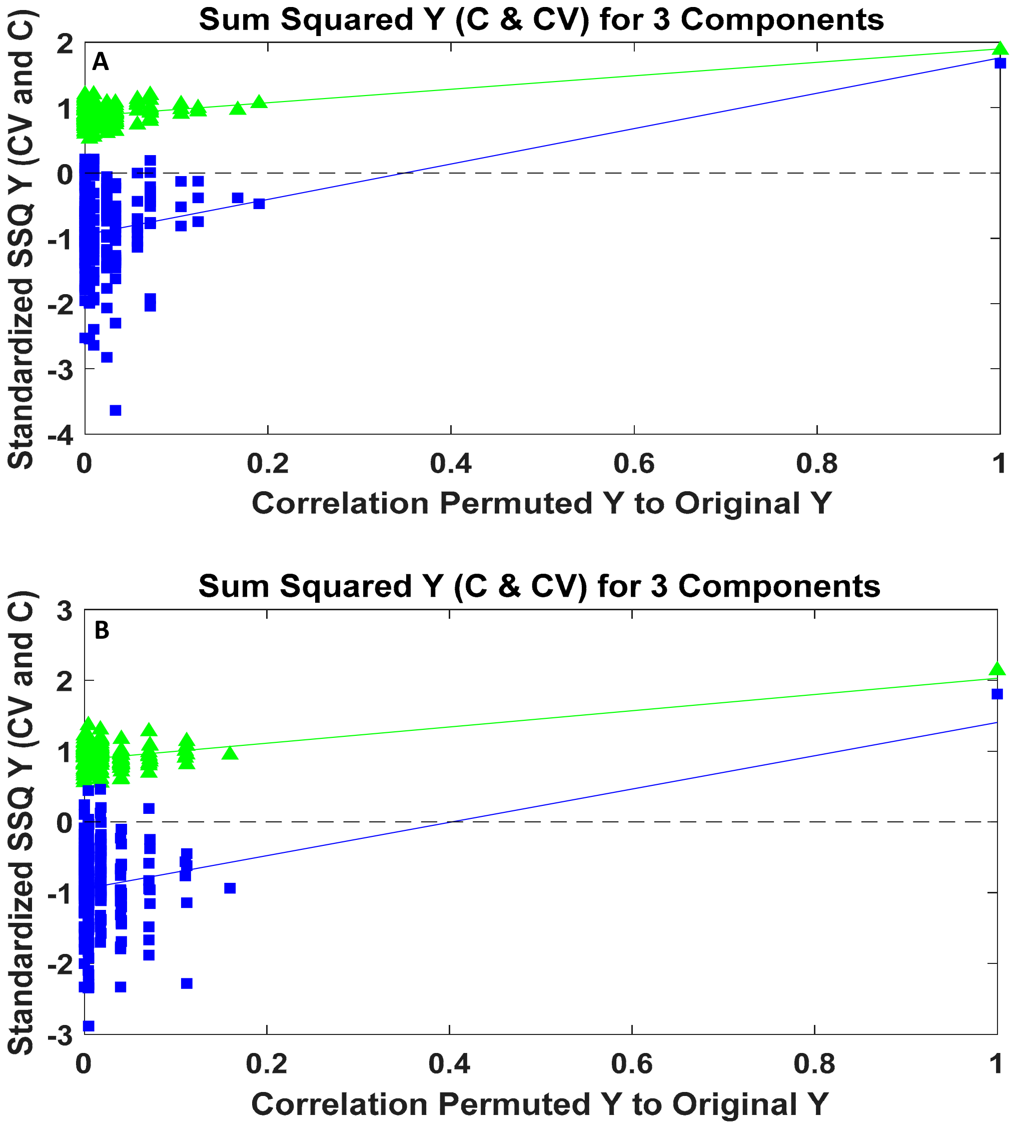

Supplementary Figure S5: Permutation test results for lipid panels for male S5A and female S5B animals to evaluate for overfitting. Random reordering of class assignments over 200 iterations provided incorrect class assignments to the data while attempting to build PLS-DA models. In general, the cross-validated results shown in blue are close to the self-predicted results shown in green and permutated results shown on the left are multiple standard deviations away from the original model shown on the far right indicating a strong lack of evidence for overfitting. Permutation tests were assessed using Wilcoxon, Rand t-test, and the sign test (p < 0.000-0.005) in cross-validation indicating that the models are significant at the 99% confidence level.

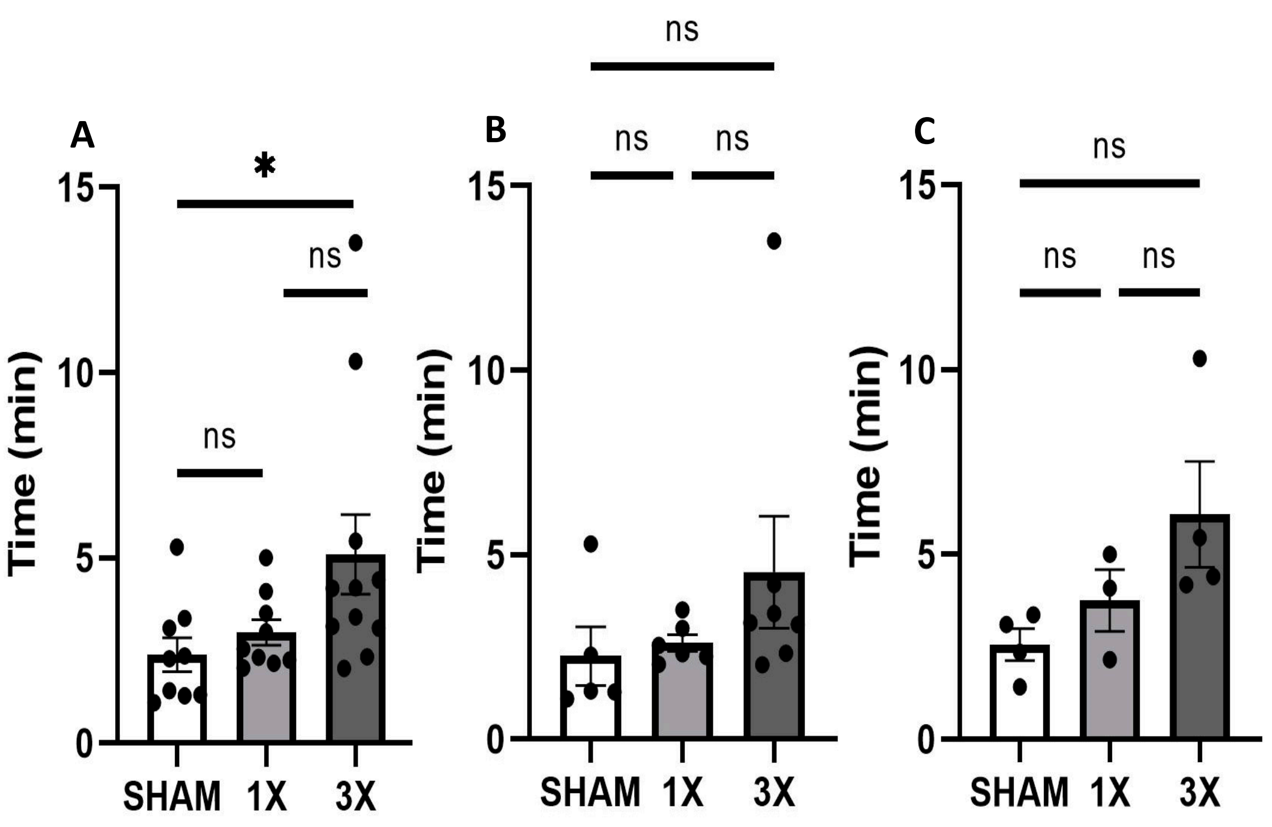

Supplementary Figure S6: Acute neurological assessment of righting reflex time for all S6A, male S6B, and female S6C animal models. Analysis between groups was conducted using a Kruskal Wallis one way analysis of variance and corrected with a post hoc Dunn test. Data are presented as bar charts with mean and standard error of the mean shown. Righting reflex times were significantly longer in repetitive mTBI groups than SHAM control across all animal models. However, when righting reflex was considered individually for each sex righting reflex times were not statistically significant.

Supplementary Table S1: Parameters for model classifiers and feature selection methods. A) Logistic regression and support vector machine hyperparameters were optimized using a grid search within the inner cross-validation loop with training data withheld by the outer loop. Recursive feature elimination was then used to select variables most relevant for classifying injured and uninjured serum samples. B) Genetic algorithm and C) interval partial least squares feature selection parameters utilized on oPLS-DA models with 3 latent variables.

A)

Parameter	Penalty Terms	Regularization Parameter (C)	Kernel Coefficient (γ)	Inner Cross-validation Splits	Outer Cross-validation Splits	RFE Variable Selection Range
Logistic Regression	L1, L2	0.001, 0.01, 0.05, 0.1, 0.5, 1.0, 10	-	5	10	19-32
Support Vector Machine	-	0.001, 0.01, 0.05, 0.1, 0.5, 1.0, 10	0.001, 0.01, 0.1, 0.15, 0.175, 0.2, 1.0, 10	5	10	19-32

B)

**Genetic Algorithm Feature Selection**	
Population Size	64
Percent of Initial Terms Included	30
Window Width	1
Target Number Maximum Variables	30
Target Number Minimum Variables	10
Penalty Slope	0.05
Maximum Generations	200
Percent Convergence	50%
Mutation Rate	0.005
Regression Method	Partial Least Squares
Cross-validation	Random
Number of Cross-validation Data Splits	8
Number of Iterations of Cross-validation	10
Replicate Runs	5

C)

**Interval PLS Feature Selection**	
Mode	Reverse
Step Size	1
Algorithm	PLS
Number of Intervals	Automatic
Interval Size	1

Supplementary Table S2: Annotated MS/MS spectra for features selected in the final male A) and female B) models distinguishing TBI and uninjured samples. Ions selected for fragmentation are underlined in the corresponding instrument polarity. Metabolite identities were assigned with the following levels of confidence: 1) compounds matched to existing standards by accurate mass, isotopic abundance, fragmentation spectrum, and retention time; 2) compounds annotated according to accurate mass, isotopic abundance, and fragmentation consistent with Lipid Maps and Human Metabolome Database (HMDB) entries; 3) accurate mass match matched to Lipid Maps and HMDB entries and fragmentation showing a few matching characteristic fragment ions.

A)

Feature ID	Fragment Ion m/z	Relative Intensity	Fragment Annotation	Specific Comments [ID level]
63<br>CE(22:5)	716.6343<br>453.0328<br>369.3494<br>173.4529	0.01<br>0.14<br>0.17<br>1.00	$[M+NH_4]^+$<br>-<br>Cholestane<br>-	Accurate mass match (Lipid Maps) [3]
89<br>Cer(d18:0/24:0)	652.6611<br>634.6459<br>368.3879<br>284.2947<br>266.2849<br>265.2710<br>264.2681<br>173.4528	0.95<br>1.00<br>0.08<br>0.17<br>0.11<br>0.19<br>0.22<br>0.20	$[M+H]^+$<br>$[M+H-H_2O]^+$<br>NL FA 18:0<br>$[FA\ 18:0+H]^+$<br>$[FA\ 18:0+H-H_2O]^+$<br>-<br>$[M+H-2H_2O-FA\ 24:0]^+$<br>-	Consistent with predicted spectrum (HMDB) [2]
258<br>LysoPI(18:0)	601.3351<br>583.3239<br>565.3137<br>533.3405<br>429.3200<br>421.2713<br>341.3049<br>335.0734<br>267.2689<br>163.4789<br>155.0102	0.03<br>0.41<br>0.16<br>0.03<br>0.03<br>0.03<br>1.00<br>0.03<br>0.01<br>0.01<br>0.15	$[M+H]^+$<br>$[M+H-H_2O]^+$<br>$[M+H-2H_2O]^+$<br>-<br>-<br>Loss of inositol ring<br>Loss of headgroup<br>Headgroup - $C_3H_5O_2$<br>$[C_{18}H_{35}O]^+$<br>Inositol ring<br>Propenol-phosphate	Consistent with predicted spectrum (HMDB) [2]
365<br>PC(18:2_19:0)	844.6080<br>784.6520<br>777.1300<br>708.8340<br>504.3801<br>369.9151<br>297.3781<br>283.3771<br>279.3021<br>232.7551<br>168.1110	1.00<br>0.30<br>0.06<br>0.09<br>0.03<br>0.03<br>0.17<br>0.03<br>0.41<br>0.03<br>0.03	$[M+HCO_2]^-$<br>$[M-CH_3]^-$<br>-<br>-<br>NL FA 18:2<br>-<br>$[FA\ 19:0+HCO_2]^-$<br>-<br>$[FA\ 18:2+HCO_2]^-$<br>-<br>Phosphocholine - CH_3	Consistent with in-house database entry [1][1], matched to feature 367 in negative mode

453<br>PC(18:0_22:5)	880.6072<br>820.6680<br>534.4890<br>508.4361<br>329.2821<br>309.2221<br>303.2471<br>283.2440<br>224.0691<br>168.2290	1.00<br>0.23<br>0.02<br>0.03<br>0.20<br>0.06<br>0.06<br>0.14<br>0.01<br>0.01	$[M+HCO_2]^-$<br>$[M-CH_3]^-$<br>-<br>Loss of FA 22:5 as ketene<br>$[FA\ 22:5-H]^-$<br>-<br>-<br>$[FA\ 18:0-H]^-$<br>GPC - CH_3-H_2O<br>Phosphocholine-CH_3	Consistent with in-house database [1]
459<br>PC(18:0_22:6)	878.5926<br>818.6650<br>508.4620<br>327.2741<br>283.3051<br>224.1081	1.00<br>0.25<br>0.05<br>0.16<br>0.25<br>0.01	$[M+HCO_2]^-$<br>$[M-CH_3]^-$<br>Loss of FA 22:6 as ketene<br>$[FA\ 22:6-H]^-$<br>$[FA\ 18:0-H]^-$<br>GPC-CH_3-H_2O	Consistent with in-house database [1]
476<br>PC(41:7)	846.6019<br>759.5728<br>184.0729<br>124.9995	0.72<br>0.16<br>1.00<br>0.09	$[M+H]^+$<br>-<br>Choline phosphate<br>$[C_2H_5PO_4+H]^+$	Consistent with in-house database entry [1]
497<br>PC(42:8)	858.6013<br>734.4346<br>572.7509<br>485.5082<br>434.7412<br>186.0770<br>184.0729<br>124.9995	1.00<br>0.06<br>0.06<br>0.06<br>0.06<br>0.07<br>0.98<br>0.10	$[M+H]^+$<br>-<br>-<br>-<br>-<br>-<br>Choline phosphate<br>$[C_2H_5PO_4+H]^+$	Consistent with in-house database [1]
527<br>PC(O-16:1/16:0)	762.5659<br>702.5413<br>464.3153<br>446.3012<br>255.2323	1.00<br>0.53<br>0.06<br>0.04<br>0.86	$[M+HCO_2]^-$<br>$[M-CH_3]^-$<br>Loss of FA 16:0 as ketene<br>NL FA 16:0 and CH_3<br>$[FA\ 16:0-H]^-$	Consistent with in-house database [1][1]
543<br>PC(O-18:2/22:6)<br>or<br>PC(P-18:1/22:6)	816.5910<br>758.5881<br>675.5391<br>550.3530<br>506.4411<br>184.1151<br>125.0041<br>104.1941	1.00<br>0.03<br>0.01<br>0.01<br>0.01<br>0.93<br>0.09<br>0.01	$[M+H]^+$<br>-<br>-<br>NL of FA O-18:2 or FA P-18:1<br>NL of FA 22:6+H_2O<br>Choline phosphate<br>$[C_2H_5PO_4+H]^+$<br>Choline	Consistent with predicted spectrum (HMDB) [2]
551<br>PC(O-18:1/18:1)	816.6511<br>756.6771<br>685.5311<br>492.5041<br>474.4060<br>403.3710	1.00<br>0.63<br>0.01<br>0.05<br>0.03<br>0.01	$[M+HCO_2]^-$<br>$[M-CH_3]^-$<br>Loss of choline and HCO_2<br>NL FA 18:1 as ketene and CH_3<br>NL FA 18:1 and CH_3<br>Loss of choline and FA 18:1	Consistent with in-house database entry [1].

	281.2901<br>255.1611<br>168.0911	0.44<br>0.03<br>0.01	[FA 18:1-H]$^-$<br>-<br>Phosphocholine - CH_3	
570<br>PC(O-38:3)	<u>798.6378</u><br>615.5211<br>593.4400<br>391.2760<br>262.3650<br>184.0911<br>167.0481<br>148.9841<br>146.9501<br>125.0261	1.00<br>0.06<br>0.04<br>0.09<br>0.02<br>0.60<br>0.03<br>0.10<br>0.03<br>0.03	[M+H]$^+$<br>NL PC headgroup<br>-<br>-<br>-<br>Choline phosphate<br>-<br>-<br>-<br>[$C_2H_5PO_4$+H]$^+$	Consistent with in-house database [1]
601<br>PC(O-18:1/22:6)	<u>862.5976</u><br>802.5701<br>492.3436<br>474.3322<br>327.2312<br>283.2412	1.00<br>0.30<br>0.09<br>0.05<br>0.25<br>0.10	[M+HCO_2]$^-$<br>[M-CH_3]$^-$<br>Loss of FA 22:6 as ketene<br>NL FA 22:6 and CH_3<br>[FA 22:6-H]$^-$<br>[FA 22:6-H]$^-$-CO_2	Consistent with in-house database [1][3]
651<br>PE(O-34:1)	<u>704.5595</u><br>563.5520<br>308.2630<br>184.0740	0.81<br>1.00<br>0.04<br>0.14	[M+H]$^+$<br>NL PE headgroup<br>-<br>Choline Phosphate	Consistent with in-house database entry [1]
652<br>PE(O-18:1/16:0)	<u>704.5585</u><br>563.5840<br>436.2441<br>392.2901<br>313.2311<br>294.2521<br>282.2801<br>184.0181	1.00<br>0.04<br>0.06<br>0.15<br>0.54<br>0.06<br>0.05<br>0.05	[M+H]$^+$<br>NL PE headgroup<br>NL FA O-18:1<br>-<br>[FA 16:0+$C_3H_5O_3$]$^+$<br>-<br>-<br>Choline Phosphate	Consistent with in-house database entry [1][2]
788<br>SM(d33:1)	<u>689.5596</u><br>184.1501<br>125.0631<br>104.1510	0.68<br>1.00<br>0.07<br>0.01	[M + H]$^+$<br>Choline Phosphate<br>[$C_2H_5PO_4$+H]$^+$<br>Choline	Consistent with predicted spectrum (Lipid Maps) [2]
792<br>SM(d34:1)	<u>703.5752</u><br>264.3511<br>184.1531<br>125.2991<br>104.4380	1.00<br>0.01<br>0.94<br>0.10<br>0.01	[M+H]$^+$<br>-<br>Choline Phosphate<br>[$C_2H_5PO_4$+H]$^+$<br>Choline	Consistent with predicted spectrum (Lipid Maps) [2]
808<br>SM(d36:3)	<u>727.5758</u><br>582.2816<br>316.6257<br>184.0729<br>173.4537<br>124.9994	0.60<br>0.03<br>0.03<br>1.00<br>0.10<br>0.06	[M+H]$^+$<br>-<br>-<br>Choline phosphate<br>-<br>[$C_2H_5PO_4$+H]$^+$	Accurate mass match (Lipid Maps) [3]

1095<br>TG(60:4)	984.8954<br>949.8510<br>939.1470<br>780.8890<br>680.5690<br>647.8100<br>369.4361<br>267.1091<br>264.4141<br>218.7111<br>102.1741	0.33<br>0.07<br>0.02<br>0.02<br>0.03<br>0.03<br>0.02<br>0.02<br>0.02<br>0.02<br>1.00	$[M+NH_4]^+$<br>$[M+H-H_2O]^+$<br>-<br>-<br>-<br>-<br>-<br>-<br>-<br>-<br>-	Accurate mass match (Lipid Maps) [3]
1114<br>TG(18:1_20:1_24:1)	1014.943<br>997.2231<br>980.0580<br>955.1960<br>936.9100<br>715.6310<br>687.5630<br>481.4121	1.00<br>0.05<br>0.16<br>0.07<br>0.09<br>0.19<br>0.05<br>0.05	$[M+NH_4]^+$<br>$[M+H]^+$<br>$[M+H-H_2O]^+$<br>-<br>-<br>NL FA 18:1 and NH_3<br>NL FA 20:1 and NH_3<br>-	Consistent with predicted spectrum (HMDB) [2][2]

B)

Feature ID	Fragment Ion m/z	Relative Intensity	Fragment Annotation	Specific Comments [ID level]
8<br>Car(5:0)	246.1700<br>228.1151<br>187.0971<br>144.1551<br>118.1160	1.00<br>0.02<br>0.09<br>0.02<br>0.03	$[M+H]^+$<br>$[M+H-H_2O]^+$<br>-<br>-<br>-	Consistent with standard in (HMDB) [1]
27<br>Car(16:1 + O)	414.3216<br>379.3641<br>102.1330	1.00<br>0.03<br>0.04	$[M+H]^+$<br>-<br>-	Accurate mass match (Lipid Maps) [3]
35<br>Car(18:1 + O)	442.3529<br>367.7886<br>304.5762<br>173.4536	1.00<br>0.03<br>0.03<br>0.05	$[M+H]^+$<br>-<br>-<br>-	Accurate mass match (Lipid Maps) [3]
103<br>Cer(d18:1/25:0)	708.6514<br>662.7590<br>614.5810<br>422.3861<br>406.5031<br>381.3791<br>380.4821<br>363.3271<br>263.2061<br>237.2990	1.00<br>0.39<br>0.02<br>0.03<br>0.09<br>0.01<br>0.02<br>0.04<br>0.01<br>0.01	$[M+HCO_2]^-$<br>$[M-H]^-$<br>$[M-HCHO-H_2O]^-$<br>NL 240.3 Sphingosine base<br>NL 256.2 Sphingosine base<br>$[FA 25:0-H]^-$<br>-<br>$[FA 25:0-H_2O-H]^-$<br>C_{18} Sphingosine fragment<br>C_{18} Sphingosine fragment	Consistent with in-house database [1]
282<br>PE(16:0_18:1)	716.5236<br>568.8780	1.00<br>0.02	$[M-H]^-$<br>-	Consistent with in-house database [1][1],

	281.2951<br>255.2870<br>139.9481	0.56<br>0.31<br>0.03	$[FA\ 18:1-H]^-$<br>$[FA\ 16:0-H]^-$<br>Ethanolamine phosphate	matched to feature 610 in negative ion mode
328<br>PC(17:0_18:2)	816.5766<br>756.6310<br>494.3041<br>293.2611<br>281.2611<br>279.2861<br>269.2411<br>267.2350<br>255.2270<br>224.1371	1.00<br>0.30<br>0.03<br>0.03<br>0.05<br>0.45<br>0.18<br>0.02<br>0.01<br>0.01	$[M+HCO_2]^-$<br>$[M-CH_3]^-$<br>Loss of FA 18:2 as ketene<br>-<br>$[FA\ 18:1-H]^-$<br>$[FA\ 18:2-H]^-$<br>$[FA\ 17:0-H]^-$<br>$[FA\ 17:1-H]^-$<br>$[FA\ 16:0-H]^-$<br>GPC-CH_3-H_2O	Consistent with in-house database [1][1], potential co-elution of multiple species, matched to feature 332 in the negative mode
346<br>PC(18:1_18:2)	828.5759<br>768.6321<br>506.3701<br>488.3891<br>281.2841<br>279.3030<br>224.1361<br>168.0581	1.00<br>0.30<br>0.03<br>0.01<br>0.22<br>0.49<br>0.01<br>0.01	$[M+HCO_2]^-$<br>$[M-CH_3]^-$<br>Loss of FA 18:2 as ketene<br>NL FA 18:2-CH_3<br>$[FA\ 18:1-H]^-$<br>$[FA\ 18:2-H]^-$<br>GPC-CH_3-H_2O<br>Phosphocholine-CH_3	Consistent with in-house database [1][1], matched to feature 349 in the negative mode
348<br>PC(16:0_20:3)	828.5767<br>768.6440<br>506.3651<br>480.3601<br>462.3390<br>305.2651<br>281.2591<br>279.2631<br>255.2741<br>224.0100<br>168.0551	1.00<br>0.29<br>0.01<br>0.03<br>0.01<br>0.38<br>0.03<br>0.09<br>0.15<br>0.01<br>0.01	$[M+HCO_2]^-$<br>$[M-CH_3]^-$<br>Loss of FA 18:2 as ketene-CH_3<br>Loss of FA 20:3 as ketene-CH_3<br>NL FA 20:3-CH_3<br>$[FA\ 20:3-H]^-$<br>$[FA\ 18:1-H]^-$<br>$[FA\ 18:2-H]^-$<br>$[FA\ 16:0-H]^-$<br>GPC-CH_3-H_2O<br>Phosphocholine-CH_3	Consistent with in-house database [1][3], co-elution of PC(18:1_18:2)
388<br>PC(18:0_20:2)	814.6237<br>796.6660<br>746.6799<br>631.6279<br>548.3790<br>530.2960<br>524.3740<br>506.5780<br>397.4550<br>369.3200<br>184.0960<br>125.0531<br>104.1811	1.00<br>0.01<br>0.01<br>0.01<br>0.01<br>0.01<br>0.01<br>0.01<br>0.01<br>0.01<br>0.84<br>0.07<br>0.01	$[M+H]^+$<br>$[M+H-H_2O]^+$<br>$[M+H-N(CH_3)_3]^+$<br>NL Phosphocholine<br>Loss of FA 18:0<br>Loss of FA 18:0-H_2O<br>Loss of FA 20:2<br>Loss of FA 20:2-H_2O<br>-<br>-<br>Choline phosphate<br>$[C_2H_5PO_4+H]^+$<br>Choline	Consistent with predicted spectrum (HMDB) [2]
437<br>PC(17:0_22:6)	864.5764<br>804.6061	1.00<br>0.38	$[M+HCO_2]^-$<br>$[M-CH_3]^-$	Consistent with in-house database [1][3]

	494.5361<br>327.2111<br>283.2271<br>269.2811	0.03<br>0.13<br>0.07<br>0.15	Loss of FA 22:6 as ketene<br>[FA 22:6-H]$^-$<br>[FA 22:6-H]$^-$-CO_2<br>[FA 17:0-H]$^-$	
455<br>PC(18:0_22:5)	880.6079<br>820.6680<br>534.4890<br>508.4361<br>329.2821<br>309.2221<br>303.2471<br>283.2440<br>224.0691<br>168.2290	1.00<br>0.23<br>0.02<br>0.03<br>0.20<br>0.06<br>0.06<br>0.14<br>0.01<br>0.01	$[M+HCO_2]^-$<br>$[M-CH_3]^-$<br>-<br>Loss of FA 22:5 as ketene<br>[FA 22:5-H]$^-$<br>-<br>-<br>[FA 18:0-H]$^-$<br>GPC-CH_3-H_2O<br>Phosphocholine-CH_3	Consistent with in-house database [1][3]
620<br>PE(18:1_18:2)	740.5241<br>478.2925<br>281.2473<br>279.2317<br>196.0366<br>173.4400<br>140.0108	1.00<br>0.04<br>0.41<br>0.83<br>0.03<br>0.04<br>0.02	$[M-H]^-$<br>Loss of FA 18:2 as ketene<br>[FA 18:1-H]$^-$<br>[FA 18:2-H]$^-$<br>-<br>-<br>Ethanolamine phosphate ion	Consistent with in-house database [1][1] Matched to feature 621 in negative ion mode.
757<br>PS(38:2)	838.5572<br>816.5530<br>518.3131<br>459.2631<br>313.2860<br>184.0780<br>147.0011	0.05<br>0.09<br>1.00<br>0.31<br>0.12<br>0.02<br>0.14	$[M+Na]^+$<br>$[M+H]^+$<br>-<br>-<br>-<br>Choline phosphate<br>-	Accurate mass match (HMDB) [3]
813<br>SM(d16:0/22:1)	803.6295<br>743.6034<br>449.3139<br>173.4412<br>168.0417	0.83<br>1.00<br>0.01<br>0.01<br>0.28	$[M+HCO_2]^-$<br>$[M-CH_3]^-$<br>Loss of FA 22:1 as ketene<br>-<br>Phosphocholine-CH_3	Consistent with in-house database [1][1]
825<br>SM(d39:2)	771.6320<br>184.1040<br>125.0390<br>104.2470	1.00<br>0.89<br>0.06<br>0.02	$[M+H]^+$<br>Choline Phosphate<br>$[C_2H_5PO_4+H]^+$<br>Choline	Consistent with predicted spectrum (Lipid Maps) [2]
874<br>Sphinganine (C18)	302.3054<br>284.2945<br>266.2840<br>254.2838<br>240.9719<br>240.2687<br>185.5219<br>173.4523	1.00<br>0.50<br>0.05<br>0.11<br>0.01<br>0.02<br>0.01<br>0.02	$[M+H]^+$<br>$[M+H-H_2O]^+$<br>$[M+H-2H_2O]^+$<br>-<br>-<br>-<br>-<br>-	Consistent with standard in (HMDB) [1]

875<br>Sphingosine (C18)	300.2897<br>282.2630<br>265.2150<br>264.2501<br>252.2441	0.07<br>1.00<br>0.01<br>0.06<br>0.08	$[M+H]^+$<br>$[M+H-H_2O]^+$<br>NL NH_3<br>$[M+H-2H_2O]^+$<br>$C_{17}H_{34}N$	Consistent with predicted spectrum (HMDB) [2]
989<br>TG(18:1_18:2_18:2)	898.7860<br>881.7991<br>601.5811<br>599.5450<br>579.5660<br>578.5190<br>339.3331<br>337.3251<br>265.2121<br>263.1971<br>245.2041	1.00<br>0.15<br>0.45<br>0.24<br>0.02<br>0.02<br>0.01<br>0.01<br>0.02<br>0.03<br>0.01	$[M+NH_4]^+$<br>$[M+H]^+$<br>NL FA 18:2+NH_3<br>NL FA 18:1+NH_3<br>-<br>-<br>FA 18:1 $[RC=O+74]^+$<br>FA 18:2 $[RC=O+74]^+$<br>FA 18:1 $[RC=O]^+$<br>FA 18:2 $[RC=O]^+$<br>FA 18:2 $[RC=O]^+-H_2O$	Consistent with in-house database [1][4]
1110<br>TG(61:4)	998.7811<br>980.7710<br>962.9290<br>936.8170<br>881.6740<br>754.1990<br>391.4181<br>371.9621<br>356.0800<br>326.3090<br>324.3600<br>283.1300	1.00<br>0.25<br>0.35<br>0.49<br>0.44<br>0.58<br>0.49<br>0.60<br>0.35<br>0.29<br>0.25<br>0.38	$[M+NH_4]^+$<br>$[M+NH_4-H_2O]^+$<br>-<br>-<br>-<br>-<br>-<br>FA 22:4 $[RC=O+74]^+-H_2O$<br>-<br>-<br>-<br>-	Accurate mass match (HMDB) [3]

[1]ID level determined by feature detected in positive mode. FA chain information was determined using a paired feature in the negative mode and the Lipid Maps prediction tool for glycerophospholipids. https://www.lipidmaps.org/tools/structuredrawing/GP_p_form.php

[2]FA chain information matched to HMDB predicted spectra

[3]FA chain information determined using the Lipid Maps prediction tool for glycerophospholipids.

[4]FA chain information determined using the Lipid Maps prediction tool for glycerolipids.

Supplementary Table S3: Summary of animal cohort characteristics including sex, injury class, blood collection time points, time to right, and time of first impact. Blood collection time points are labelled sequentially from baseline (A), 30 min post-injury (B), 4 h post-injury (C), and 24 h post injury (D). Time to right indicates the time required for the study subject to return to its feet when placed on its back after the final injury. Sham animals were not injured but were treated identically to injured animals, with the impact time for sham animals corresponding to the end of isoflurane administration.

	**Sex**	**Injury**	**Blood Collection**	**Time to Right (s)**	**Impact Time of Day**
1	Male	Sham	C, D	2.36	12:38
2	Male	Sham	A, B, C, D	1.42	13:37
3	Male	Single Impact	A, C, D	2.34	14:34
4	Male	Repeat Impact	A, C, D	4.18	16:09
5	Male	Sham	A, B, C, D	3.10	12:45
6	Male	Single Impact	A, B, C, D	4.10	13:35
7	Male	Sham	A, B, C, D	3.37	14:33
8	Male	Single Impact	A, B, C, D	2.16	15:29
9	Male	Repeat Impact	A, C, D	10.31	11:48
10	Male	Single Impact	A, C, D	5.01	12:51
11	Male	Repeat Impact	A, B, C, D	4.40	13:05
12	Male	Repeat Impact	A, B, C, D	5.46	13:56
13	Female	Single Impact	A, B, C	2.54	10:08
14	Female	Sham	A, B, C, D	5.30	10:57
15	Female	Single Impact	A, B, C	2.03	09:18
16	Female	Repeat Impact	A, B, C	3.40	10:08
17	Female	Single Impact	A, B, C, D	3.00	13:44
18	Female	Sham	A, B, C, D	1.30	14:28
19	Female	Sham	B, C, D	1.09	15:15
20	Female	Repeat Impact	A, B, C, D	3.10	16:10
21	Female	Sham	C, D	1.28	17:02
22	Female	Single Impact	A, C, D	3.50	09:33
23	Female	Repeat Impact	A, B, C, D	2.33	12:33
24	Female	Repeat Impact	A, B, C	3.16	13:36
25	Male	Sham	A, B, C, D	2.53	11:25
26	Male	Sham	A, B, C, D	2.45	14:02
27	Female	Single Impact	A, B, C, D	2.24	09:54
28	Female	Repeat Impact	A, B, C, D	13.5	11:22
29	Female	Single Impact	A, B, C, D	2.32	12:12
30	Female	Repeat Impact	A, B, C, D	4.19	13:08
31	Female	Sham	A, B, C, D	2.28	11:53
32	Female	Repeat Impact	A, B, C, D	2.02	12:41

Supplementary Table S4: LC-MS method information: A) Chromatographic gradient (mobile Phase A: water and acetonitrile [40:60] and B: isopropanol and acetonitrile [90:10]) both phases contained 10 mM acetonitrile and 0.1% formic acid. Positive and negative ion modes utilized identical solvent gradients. B) Summary of MS acquisition parameters. C) Summary of data dependent acquisition parameters. QC and Reference samples were analyzed with two DDA methods per polarity. Each polarity utilized one method with an inclusion list and a second method which did not. All methods employed an exclusion list.

A)

**Positive or Negative Mode**		
**Time (min)**	**%A**	**%B**
0	80	20
1	40	60
5	30	70
5.5	15	85
8	10	90
8.2	0	100
10.5	0	100
10.7	80	20
12	80	20

B)

**Parameter**	**Negative Mode**	**Positive Mode**
Ion Source Type	ESI	ESI
Ion Voltage	-2500 V	3500 V
Ion Transfer Tube Temperature	275 ˚C	275 ˚C
Vaporizer Temperature	320 ˚C	320 ˚C
Detector Type	Orbitrap	Orbitrap
Orbitrap Resolution	120000	120000
Scan Range	150-2000 *m/z*	150-2000 *m/z*
Maximum Ion Injection Time	50 ms	200 ms
RF Lens	40%	40%

C)

**Parameter**	**Positive Mode DDA**		**Negative Mode DDA**	
Isolation Window	0.4 m/z		0.4 m/z	
Activation Type	HCD		HCD	
HCD Collision Energies (%)	10, 30, 50		10, 30, 50	
Detector Type	Ion Trap	Orbitrap	Ion Trap	Orbitrap
Maximum Injection Time	35 ms	54 ms	100 ms	150 ms
Ion Trap Scan Rate	Rapid		Rapid	
Orbitrap Resolution	30000		30000	
Targeted Mass Inclusion Tolerance	5 ppm		5 ppm	
Targeted Mass Exclusion Tolerance	15 ppm		10 ppm	
Dynamic Exclusion Tolerance	5 ppm		10 ppm	

Supplementary Table S5: Table of annotated lipid compounds with ionization mode and retention time. Median FC from baseline timepoints are shown where positive numbers correspond to features with greater abundance in post baseline blood collected and negative numbers corresponding to greater abundance in the baseline blood samples.

Feature	Mode	Name	RT [min]	SHAM 30 min	SHAM 4 h	SHAM 24 h	Single Impact 30 min	Single Impact 4 h	Single Impact 24 h	Repeat Impact 30 min	Repeat Impact 4 h	Repeat Impact 24 h
1	RPPos	arginine	0.73	-1.147	-1.228	-1.324	-1.667	-1.713	-1.454	-1.927	-1.252	-1.195
2	RPPos	bilirubin	2.53	1.104	1.215	-1.152	1.088	1.508	-1.022	1.022	1.256	1.432
3	RPPos	bis(2-ethylhexyl)phthalate	8.91	1.145	-1.009	1.159	1.028	1.013	-1.358	1.190	1.486	1.406
4	RPPos	carnitine	0.79	1.171	-1.162	1.192	1.032	1.104	2.045	-1.236	-1.293	1.238
5	RPPos	Car(2:0)	0.80	1.508	-1.020	1.012	1.962	1.939	1.298	1.288	1.269	1.455
6	RPPos	Car(3:0)	0.80	-1.013	1.020	-1.097	1.319	1.265	1.746	-1.473	-1.172	-1.069
7	RPPos	Car(4:0)	0.90	1.054	-1.481	-1.340	1.146	-1.322	1.021	-1.729	-1.477	-1.153
8	RPPos	Car(5:0)	0.81	-1.041	-1.316	-1.700	-1.206	1.236	-1.175	-1.338	-1.638	-1.321
9	RPPos	Car(6:0)	0.82	1.474	-1.092	1.161	1.022	-1.109	-1.037	1.189	1.188	1.246
10	RPPos	Car(8:0)	0.91	1.234	-1.107	1.325	1.739	1.119	1.777	2.096	1.474	1.836
11	RPPos	Car(10:0)	0.96	1.146	1.042	1.065	1.094	1.180	1.195	1.879	1.379	1.606
12	RPPos	Car(10:1)	0.93	1.478	1.068	1.029	-1.272	-1.261	-1.367	1.595	1.381	1.845
13	RPPos	Car(12:0)	1.14	1.261	1.336	1.000	1.133	1.145	-1.044	1.434	1.382	1.235
14	RPPos	Car(12:1)	1.04	1.230	1.342	-1.112	-1.104	1.130	-1.116	1.352	1.522	1.390
15	RPPos	Car(13:0)	1.25	1.477	1.727	-1.044	1.062	-1.153	-1.238	1.266	1.343	1.001
16	RPPos	Car(13:1)	1.13	1.355	1.491	1.030	-1.377	1.010	-1.120	1.487	1.145	1.078
17	RPPos	Car(14:0)	1.52	1.290	1.172	1.016	1.396	1.233	-1.062	1.611	1.470	1.015
18	RPPos	Car(14:0-OH)	1.19	1.576	1.405	-1.121	1.340	1.198	-1.438	1.885	2.061	1.082
19	RPPos	Car(14:1)	1.28	1.297	1.385	1.079	1.129	1.140	-1.072	1.637	1.583	1.229
20	RPPos	Car(14:1-OH)	1.07	1.410	1.077	-1.594	1.006	-1.023	-1.389	1.828	1.278	1.143
21	RPPos	Car(14:2)	1.14	1.237	1.516	1.117	-1.006	1.323	1.182	1.450	1.308	1.186
22	RPPos	Car(14:2-OH)	0.98	1.047	1.233	-1.245	-1.601	-1.196	-1.517	1.166	1.327	1.033
23	RPPos	Car(15:0)	2.47	1.062	-1.167	-1.370	1.150	1.371	-1.270	1.540	1.450	-1.063
24	RPPos	Car(16:0)	1.93	1.270	1.222	-1.063	1.319	1.421	-1.249	1.373	1.961	1.327
25	RPPos	Car(16:0-OH)	1.58	1.256	1.289	-1.039	1.330	1.397	-1.120	1.352	1.628	-1.202
26	RPPos	Car(16:1)	1.63	1.447	1.230	1.187	1.664	1.335	-1.358	2.185	2.119	1.270
27	RPPos	Car(16:1-OH)	1.33	1.226	1.243	-1.107	1.400	1.266	-1.254	1.691	1.660	1.090
28	RPPos	Car(16:2)	1.38	1.505	1.308	1.197	1.539	1.307	-1.150	1.919	1.855	1.730
29	RPPos	Car(16:3)	1.25	1.237	1.032	1.012	-1.231	1.096	1.163	1.270	-1.010	-1.169
30	RPPos	Car(17:0)	2.10	-1.166	1.230	-1.368	1.508	1.555	-1.323	1.359	2.207	1.102
31	RPPos	Car(18:0)	2.24	-1.057	1.226	1.007	1.033	1.254	-1.158	1.098	1.457	1.048

32	RPPos	Car(18:0-OH)	2.00	1.572	1.159	-1.027	1.610	1.089	-1.589	1.750	1.957	1.385
33	RPPos	Car(18:1)	2.00	1.593	1.157	-1.017	1.602	1.093	-1.595	1.802	2.008	1.389
34	RPPos	Car(18:1-2OH)	1.12	1.092	2.071	-1.063	1.264	1.299	-1.277	1.455	2.229	1.234
35	RPPos	Car(18:1-OH)	1.70	1.926	1.175	-1.103	1.684	1.262	-1.470	2.578	2.019	1.455
36	RPPos	Car(18:2)	1.73	1.341	1.256	1.021	1.350	1.185	-1.246	1.956	2.228	1.850
37	RPPos	Car(18:2-2OH)	1.02	-1.097	1.573	-1.126	-1.226	1.018	-1.218	1.337	1.476	-1.165
38	RPPos	Car(18:2-OH)	1.43	1.922	1.378	1.171	1.581	1.259	-1.307	2.677	2.491	1.697
39	RPPos	Car(18:3)	1.53	1.304	1.311	1.080	1.271	1.097	-1.320	2.296	1.936	1.649
40	RPPos	Car(19:0)	2.13	-1.047	-1.159	-1.032	-1.021	-1.255	1.063	-1.068	-1.298	1.235
41	RPPos	Car(19:1)	2.15	1.207	1.391	-1.329	1.216	1.163	-1.271	1.690	1.778	1.408
42	RPPos	Car(20:1-OH)	2.08	1.499	1.247	-1.113	1.257	1.278	1.019	1.885	2.112	2.133
43	RPPos	Car(20:2)	2.08	1.654	1.365	-1.140	1.274	1.318	-1.084	2.711	2.612	2.407
44	RPPos	Car(20:3-OH)	1.59	1.236	2.183	-1.112	1.088	2.344	-1.229	1.441	2.366	-1.015
45	RPPos	Car(20:4)	1.72	1.512	1.372	1.380	1.276	1.467	-1.184	1.790	2.683	1.610
46	RPPos	Car(20:5)	1.49	1.543	1.408	1.121	1.219	1.152	-1.450	1.494	1.875	1.213
47	RPPos	Car(21:2)	2.49	-1.140	-1.101	1.094	-1.029	-1.097	-1.027	1.037	-1.063	-1.140
48	RPPos	Car(22:0)	2.84	-1.055	-1.067	-1.107	-1.055	-1.146	-1.114	-1.005	-1.133	1.363
49	RPPos	Car(22:1)	2.51	1.349	1.633	-1.168	-1.263	-1.168	-1.321	1.624	1.528	1.963
50	RPPos	Car(24:0)	3.29	-1.054	1.205	1.078	-1.074	1.036	1.186	-1.080	-1.012	1.589
51	RPPos	Car(24:1)	2.83	-1.080	1.471	-1.028	1.155	-1.119	1.019	-1.082	-1.088	1.613
52	RPPos	Car(26:1)	3.26	-1.067	1.426	1.097	-1.094	1.046	1.252	-1.027	-1.130	1.742
53	RPPos	CE(16:1)	9.11	1.042	1.005	1.060	1.051	-1.049	1.070	1.014	1.020	-1.258
54	RPPos	CE(18:0)	9.70	-1.210	1.107	1.190	1.225	1.076	1.120	1.010	1.097	1.176
55	RPPos	CE(18:1)	9.47	1.063	-1.029	1.072	-1.021	-1.095	1.245	1.047	-1.001	1.106
56	RPPos	CE(18:2)	9.16	1.050	1.041	1.048	1.009	1.034	1.149	1.037	1.035	1.127
57	RPPos	CE(18:3)	7.43	-1.065	-1.426	-1.232	-1.035	-1.144	-1.038	-1.427	-1.216	-1.709
58	RPPos	CE(18:3)	8.76	1.207	1.795	1.191	1.321	3.466	1.661	1.456	2.058	1.414
59	RPPos	CE(19:2)	9.31	1.223	1.019	1.089	1.253	1.223	1.092	1.199	1.357	1.004
60	RPPos	CE(20:4)	8.88	1.115	1.235	1.180	1.124	1.414	1.274	1.163	1.512	1.435
61	RPPos	CE(20:5)	8.53	1.330	1.392	1.544	1.176	2.418	1.650	1.438	1.923	1.784
62	RPPos	CE(20:5)	8.53	1.308	1.380	1.539	1.209	2.895	1.724	1.461	1.935	1.697
63	RPPos	CE(22:5)	8.89	-1.189	-1.082	1.231	1.152	1.449	1.607	1.193	1.340	1.221
64	RPPos	CE(22:6)	8.65	1.084	1.318	1.233	1.216	1.609	1.526	1.264	1.694	1.508
65	RPPos	CE(22:6)	8.65	1.118	1.372	1.257	1.207	1.689	1.528	1.302	1.779	1.604
66	RPPos	Cer(d32:0)	4.53	1.249	1.448	1.175	1.117	-1.061	1.526	-1.060	-1.242	1.679
67	RPPos	Cer(d34:0)>Cer(18:0/16:0)	5.19	-1.709	-1.560	-1.544	-2.200	-1.656	-1.078	-2.071	-2.052	-1.345
68	RPNeg	Cer(d34:1)	4.95	-1.003	-1.017	-1.116	-1.232	-1.481	-1.071	-1.273	-1.308	-1.060
69	RPPos	Cer(d34:1)>Cer(d18:1/16:0)	4.90	-1.077	1.027	-1.139	-1.306	-1.601	-1.367	-1.485	-1.251	-1.000

70	RPPos	Cer(d36:1)>Cer(d18:1/18:0)	5.78	1.046	1.266	-1.023	1.080	-1.054	-1.110	1.009	1.530	1.646
71	RPNeg	Cer(d38:1)	6.63	1.065	-1.040	-1.225	-1.228	1.023	-1.109	-1.143	1.204	1.327
72	RPPos	Cer(d38:1)>Cer(d18:1/20:0)	6.57	1.089	1.093	-1.072	-1.062	-1.260	-1.323	-1.110	1.289	1.070
73	RPPos	Cer(d39:1)>Cer(d18:1/21:0)	6.77	-1.047	1.067	-1.059	-1.178	1.020	1.223	-1.099	1.037	1.114
74	RPPos	Cer(d40:0)>Cer(d18:0/22:0)	7.01	-1.324	-1.289	-1.265	-1.316	-1.281	-1.107	-1.365	-1.308	-1.382
75	RPNeg	Cer(d40:1)	6.98	1.059	1.064	-1.093	1.056	1.099	1.171	-1.104	1.014	1.003
76	RPPos	Cer(d40:1)>Cer(d18:1/22:0)	6.91	1.063	1.049	-1.082	1.095	1.119	1.094	1.000	1.026	-1.035
77	RPNeg	Cer(d40:2)	6.73	1.016	-1.096	-1.228	1.060	1.027	-1.179	-1.124	-1.038	1.058
78	RPNeg	Cer(d40:2)	6.62	1.086	-1.054	-1.053	1.031	-1.010	-1.020	1.011	-1.018	1.208
79	RPPos	Cer(d40:2)>Cer(d18:1/22:1)	6.55	1.162	-1.045	-1.050	1.116	-1.057	1.046	-1.108	-1.034	1.088
80	RPPos	Cer(d40:2)>Cer(d18:2/22:0)	6.67	1.098	-1.096	-1.167	1.051	-1.152	-1.318	-1.065	-1.351	-1.077
81	RPPos	Cer(d41:0)>Cer(d18:0/23:0)	7.15	-1.457	-1.199	-1.260	-1.427	1.005	1.193	-1.390	-1.454	-1.429
82	RPNeg	Cer(d41:1)	7.12	1.077	1.230	1.040	1.219	1.239	1.176	1.000	1.162	1.097
83	RPPos	Cer(d41:1)>Cer(d18:1/23:0)	7.05	1.102	1.153	1.094	1.185	1.286	1.193	1.085	1.170	1.126
84	RPNeg	Cer(d41:2)	6.89	1.152	1.123	1.014	1.190	1.225	-1.010	-1.098	1.076	1.187
85	RPNeg	Cer(d41:2)	6.80	-1.009	-1.030	-1.197	1.246	1.029	1.208	1.059	-1.030	1.028
86	RPPos	Cer(d41:2)>Cer(d18:1/23:1)	6.74	1.011	1.051	1.041	1.104	1.100	-1.061	1.034	1.000	1.053
87	RPPos	Cer(d41:2)>Cer(d18:2/23:0)	6.83	1.025	-1.015	1.001	1.181	1.040	-1.036	1.025	-1.114	1.025
88	RPNeg	Cer(d42:0)	7.37	-1.406	-1.428	-1.181	-1.425	-1.337	1.054	-1.597	-1.588	-1.490
89	RPPos	Cer(d42:0)>Cer(d18:0/24:0)	7.30	-1.275	-1.346	-1.261	-1.335	-1.305	1.063	-1.380	-1.553	-1.329
90	RPNeg	Cer(d42:0-OH)	7.11	-1.162	-1.067	-1.189	-1.223	-1.165	-1.073	-1.405	-1.514	-1.360
91	RPNeg	Cer(d42:1)	7.26	1.145	1.104	-1.029	1.138	1.182	1.082	1.055	1.118	1.084
92	RPNeg	Cer(d42:1)	7.26	1.135	1.083	-1.075	1.116	1.140	1.094	1.064	1.128	1.024
93	RPNeg	Cer(d42:1)	7.05	-1.366	-1.158	-1.405	-1.194	-1.311	-1.177	-1.363	-1.131	-1.629
94	RPPos	Cer(d42:1)>Cer(d18:0/24:1)	6.98	-1.133	-1.059	-1.258	-1.151	-1.061	-1.095	-1.280	-1.041	-1.541
95	RPPos	Cer(d42:1)>Cer(d18:1/24:0)	7.20	1.133	1.090	-1.034	1.138	1.193	1.102	1.067	1.153	1.108
96	RPNeg	Cer(d42:2)	6.95	1.137	1.023	-1.051	1.193	1.090	1.003	1.024	1.037	1.048
97	RPNeg	Cer(d42:2)	7.04	1.002	-1.216	-1.170	1.234	1.298	1.042	1.009	1.188	-1.102
98	RPNeg	Cer(d42:2)	6.95	1.137	1.051	-1.057	1.189	1.085	1.003	1.017	1.036	1.024
99	RPPos	Cer(d42:2)>Cer(d18:1/24:1)	6.88	1.118	1.009	-1.069	1.166	1.147	1.015	1.029	1.024	-1.082
100	RPPos	Cer(d42:3)>Cer(d18:1/24:2) and Cer(d18:2/24:1)	6.62	1.072	-1.072	-1.160	1.064	1.016	-1.065	-1.017	-1.100	-1.099
101	RPPos	Cer(d43:0)>Cer(d18:0/25:0)	7.40	-1.329	-1.410	-1.401	1.015	-1.372	1.200	-1.472	-1.310	-1.172
102	RPNeg	Cer(d43:1)	7.41	-1.214	-1.061	-1.164	1.088	1.141	1.516	1.027	1.059	1.318
103	RPNeg	Cer(d43:1)	7.35	1.154	1.273	1.242	1.019	1.271	1.062	-1.111	1.260	1.203
104	RPPos	Cer(d43:1)>Cer(d18:1/25:0)	7.29	1.201	1.255	1.122	1.050	1.366	1.099	1.128	1.296	1.352
105	RPPos	Cer(d43:2)>Cer(d18:1/25:1)	7.04	1.164	1.212	1.114	1.033	1.199	1.222	1.061	1.128	1.090

106	RPNeg	Cer(d44:1)	7.57	1.131	1.073	1.093	1.100	-1.002	1.373	-1.050	-1.005	1.229
107	RPPos	Cer(d44:1)>Cer(d18:1/26:0)	7.50	1.087	1.200	-1.004	1.130	1.061	1.460	1.027	1.144	1.433
108	RPNeg	Cer(d44:2)	7.23	1.155	1.169	1.125	1.055	1.028	1.326	1.011	-1.076	1.185
109	RPPos	Cer(d44:2)>Cer(d18:1/26:1)	7.16	1.167	1.163	1.090	1.090	1.117	1.323	1.024	1.005	1.213
110	RPPos	Cer(d44:3)	6.94	1.050	-1.005	1.058	1.099	1.132	1.282	1.131	-1.061	1.528
111	RPPos	Cer(t42:0)>Cer(t18:0/24:0)	7.05	-1.197	-1.069	-1.150	-1.232	-1.192	-1.036	-1.326	-1.608	-1.294
112	RPPos	Cer(t42:1)>Cer(t18:0/24:1)	6.73	-1.209	-1.028	-1.217	-1.163	-1.226	-1.033	-1.214	-1.339	-1.273
113	RPPos	cholesterol	2.67	-1.173	-1.098	-1.167	-1.223	-1.070	1.266	-1.743	-1.463	-1.469
114	RPPos	coenzyme(Q8)	6.96	1.049	1.083	-1.180	-1.147	-1.473	-1.057	1.105	-1.046	1.506
115	RPPos	coenzyme(Q9)	7.40	1.028	1.056	-1.076	-1.023	-1.326	1.053	1.104	1.222	1.560
116	RPPos	DG(34:1)>DG(16:0_18:1_0:0)	6.48	-1.593	-1.870	-1.630	-1.194	-3.201	-1.494	-1.997	-2.352	-2.511
117	RPPos	DG(34:2)>DG(16:0_18:2_0:0)	5.87	-1.377	-1.497	-1.454	-1.303	-2.497	-1.361	-1.831	-1.826	-1.901
118	RPPos	DG(35:0)	6.63	1.029	-1.094	-1.114	1.088	-1.152	-1.016	-1.075	-1.130	-1.061
119	RPPos	DG(36:1)>DG(18:0_18:1_0:0)	6.86	-2.035	-1.942	-1.629	-1.322	-2.098	-1.426	-2.686	-2.982	-1.915
120	RPPos	DG(36:2)>DG(18:1_18:1_0:0)	6.51	-1.302	-1.592	-1.324	-1.067	-2.776	-1.447	-1.493	-2.274	-1.980
121	RPPos	DG(36:3)>DG(18:1_18:2_0:0)	5.93	-1.272	-1.444	-1.460	-1.055	-2.265	-1.366	-1.645	-1.712	-1.748
122	RPPos	DG(36:4)>DG(16:0_20:4_0:0)	5.66	-1.775	-1.365	-1.128	-1.457	-1.052	1.100	-1.787	-1.018	1.667
123	RPPos	DG(36:4)>DG(18:2_18:2_0:0)	5.24	-1.259	-1.305	-1.298	-1.105	-1.964	-1.185	-1.632	-1.342	-1.508
124	RPPos	DG(38:2)	6.51	-1.244	-1.941	1.066	-1.267	-4.056	-1.458	-1.785	-2.297	-3.034
125	RPPos	DG(38:2)	6.86	-1.975	-2.125	-1.503	-2.088	-2.366	-1.284	-2.608	-4.890	-1.725
126	RPPos	DG(38:3)>DG(18:2_20:1_0:0)	6.60	-1.704	-1.636	-1.629	-1.242	-1.963	-1.101	-2.202	-3.012	-1.791
127	RPPos	DG(38:4)>DG(18:0_20:4_0:0)	6.49	-1.208	-1.187	-1.151	1.108	-1.337	-1.002	-1.132	-1.198	-1.015
128	RPPos	DG(38:5)>DG(18:1_20:4_0:0)	5.72	-1.049	-1.110	-1.077	1.088	-1.096	1.146	1.012	1.529	1.203
129	RPPos	DG(38:6)>DG(16:0_22:6_0:0)	5.41	-1.874	-1.329	-1.504	-1.468	-1.460	1.178	-2.056	-1.427	-1.367
130	RPPos	DG(38:6)>DG(18:2_20:4_0:0)	5.05	1.100	-1.201	1.376	1.139	2.239	1.608	1.437	3.986	2.557
131	RPPos	DG(38:7)	4.52	1.037	-1.105	1.234	1.123	1.237	1.543	-1.159	1.939	1.280
132	RPPos	DG(40:5)	6.34	-1.014	-1.171	-1.169	-1.437	-1.974	-1.278	-1.371	-1.532	-1.501
133	RPPos	DG(40:6)>DG(18:0_22:6_0:0)	5.78	-1.024	-1.468	-1.181	-1.229	-1.557	-1.021	-1.361	-1.304	-1.638
134	RPPos	DG(40:7)>DG(18:1_22:6_0:0)	5.47	-1.014	-1.330	1.031	-1.151	-1.289	1.179	-1.414	-1.115	-1.089
135	RPPos	DG(40:8)	4.81	1.300	-1.006	1.344	-1.023	1.106	1.211	-1.379	1.079	1.209
136	RPPos	DG(41:1)	6.90	-1.298	1.058	-1.360	-1.191	-2.086	-1.192	-1.148	-1.226	-1.098
137	RPPos	DG(42:3)	7.19	-2.605	-1.351	1.099	-1.666	1.012	1.124	-3.952	-8.008	-1.682
138	RPPos	DG(42:6)	6.92	-1.266	-1.054	-1.177	-1.289	-1.034	-1.134	-1.044	1.074	1.077
139	RPPos	DG(42:7)	6.04	-1.549	-1.781	-1.337	-1.443	-2.626	-1.465	-1.752	-3.872	-2.389
140	RPNeg	FA(16:0)	2.75	1.256	1.228	-1.211	1.114	1.102	-1.408	2.218	1.750	1.252
141	RPNeg	FA(16:1-2OH)	1.55	1.329	1.275	-1.278	1.044	1.075	-1.187	1.703	1.318	-1.034

142	RPNeg	FA(16:1-OH)	2.11	-1.382	-1.378	-2.063	-1.866	-2.999	-3.544	-1.209	-1.488	-1.487
143	RPNeg	FA(16:2)	1.26	-1.059	1.274	-1.093	-1.335	1.086	-1.017	-1.194	-1.088	-1.093
144	RPNeg	FA(16:2-2OH)	1.36	-1.073	1.115	-1.019	1.066	1.540	1.590	1.148	1.031	-1.160
145	RPNeg	FA(18:0)	3.18	1.006	-1.008	-1.548	-1.020	1.011	-1.453	1.304	1.423	1.037
146	RPNeg	FA(18:0-2OH)	1.55	-1.183	1.083	-1.603	-3.651	-2.500	-6.770	1.072	1.006	-1.400
147	RPNeg	FA(18:0-2OH)	1.64	1.045	1.014	-2.011	-2.539	-3.493	-7.094	1.139	-1.048	-1.192
148	RPNeg	FA(18:0-OH)	2.65	-1.206	-1.237	-1.637	-1.891	-3.020	-5.207	-1.205	-1.031	-1.851
149	RPNeg	FA(18:1)	2.79	1.769	1.151	-1.573	1.237	-1.221	-1.975	2.231	2.263	1.583
150	RPNeg	FA(18:1-2OH)	1.95	1.428	1.096	-1.188	1.836	1.311	-1.008	2.957	1.531	1.058
151	RPNeg	FA(18:1-OH)	2.28	1.159	1.576	-5.665	-6.908	-11.074	-34.182	-3.565	-2.111	-6.896
152	RPNeg	FA(18:1-OH)	1.87	1.337	1.062	-1.713	-1.251	-1.231	-1.530	1.344	1.715	-1.154
153	RPNeg	FA(18:2)	2.53	1.473	1.216	-1.424	1.178	-1.214	-1.792	1.919	1.881	1.565
154	RPNeg	FA(18:2-2OH)	1.71	1.024	1.214	-1.195	-1.205	-1.107	-1.151	1.205	-1.359	-1.412
155	RPNeg	FA(18:2-OH)	2.22	-1.059	-1.427	-2.915	-1.190	-3.797	-7.477	-1.737	-1.634	-2.725
156	RPNeg	FA(18:2-OH)	1.75	1.214	-1.034	-1.995	-4.323	-2.613	-3.072	-1.566	-1.318	-1.865
157	RPNeg	FA(18:2-OH)	1.60	1.162	-1.446	-1.959	-1.386	-1.208	-1.673	-1.375	1.068	-1.402
158	RPNeg	FA(18:3-2OH)	1.47	-1.108	1.242	-1.321	-1.236	-1.094	-1.128	1.133	-1.290	-1.210
159	RPNeg	FA(20:3-OH)	2.20	1.609	-1.037	1.350	1.541	1.504	1.341	2.599	2.317	1.118
160	RPNeg	FA(20:4)	2.48	1.200	1.041	1.033	1.463	1.311	-1.104	1.228	1.467	1.193
161	RPNeg	FA(20:4)	2.57	1.185	1.022	1.032	1.463	1.298	-1.102	1.222	1.413	1.171
162	RPNeg	FA(20:4-OH)	1.85	-1.192	-1.091	1.494	1.318	-1.010	-1.129	1.139	1.387	1.023
163	RPNeg	FA(20:5)	1.87	-1.129	-1.163	1.136	1.202	1.174	1.032	1.227	1.276	1.378
164	RPNeg	FA(20:5-OH)	1.64	-1.093	1.138	1.467	-1.108	1.258	-1.708	1.129	1.419	-1.655
165	RPNeg	FA(22:4)	2.73	1.637	-1.001	-1.151	1.518	1.443	-1.273	1.874	2.655	1.737
166	RPNeg	FA(22:4-OH)	2.35	1.346	-1.009	1.138	1.380	1.174	1.056	1.392	1.877	-1.050
167	RPNeg	FA(22:5)	2.53	2.180	1.431	1.149	1.383	1.182	-1.762	1.773	2.255	1.159
168	RPNeg	FA(22:5-OH)	2.20	1.548	1.270	1.451	1.635	1.490	1.047	2.078	2.574	1.586
169	RPNeg	FA(22:5-OH)	1.89	1.207	1.136	-1.009	-1.388	1.003	-1.371	1.053	1.148	1.002
170	RPNeg	FA(22:5-OH)	2.00	1.130	1.464	1.230	-1.399	-1.100	-1.062	1.200	1.822	1.227
171	RPNeg	FA(22:6)	2.43	1.413	1.110	1.017	1.141	1.393	-1.179	1.559	2.150	1.560
172	RPNeg	FA(22:6-OH)	1.78	1.127	1.071	1.111	-1.365	-1.249	-1.717	1.015	1.565	-1.231
173	RPNeg	FA(24:1)	4.50	-1.591	1.619	-2.571	-1.543	1.172	-1.049	-1.675	-3.353	-1.242
174	RPPos	glycocholate	1.00	1.014	-2.007	-1.694	1.258	-1.887	-1.118	-2.139	-1.739	-1.481
175	RPNeg	HerCer(d34:1)	4.28	1.093	1.162	1.036	1.022	1.206	1.101	-1.026	1.417	1.293
176	RPNeg	HexCer(d38:1)	5.94	1.234	1.061	1.057	1.065	1.105	1.342	-1.069	1.010	1.497
177	RPNeg	HexCer(d40:1)	6.67	1.077	1.054	-1.001	-1.005	1.117	1.202	-1.061	1.056	1.221

178	RPPos	HexCer(d42:0-OH)> HexCer(d18:0/24:0-OH)	6.78	-1.055	-1.109	-1.065	-1.092	-1.069	1.066	-1.088	-1.092	-1.083
179	RPNeg	HexCer(d42:1)	6.97	1.027	1.036	1.018	-1.024	1.039	1.085	-1.034	1.027	1.166
180	RPPos	HexCer(d42:1-OH)	6.30	-1.019	1.015	-1.087	-1.163	-1.006	-1.045	-1.016	1.025	1.043
181	RPNeg	HexCer(d42:1-OH)	6.35	1.069	1.059	-1.052	-1.060	1.034	1.098	-1.050	1.025	1.040
182	RPNeg	HexCer(d42:2)	6.63	1.182	1.039	1.019	-1.157	-1.035	1.088	-1.083	1.052	1.145
183	RPNeg	HexCer(d42:2)	6.77	-1.033	-1.061	-1.133	1.040	1.163	1.073	-1.059	-1.047	1.105
184	RPNeg	HexCer(d42:3)	6.05	1.011	-1.085	-1.160	-1.078	1.036	1.085	-1.063	1.041	1.157
185	RPNeg	HexCer(d44:2)	6.95	-1.095	1.240	1.212	-1.083	1.120	1.752	1.086	1.020	2.013
186	RPPos	HexCer(t40:0-OH)	6.19	-1.146	-1.029	-1.139	-1.033	-1.102	-1.014	-1.056	-1.108	1.025
187	RPPos	HexCer(t42:0-OH)	6.72	-1.136	-1.081	-1.200	-1.064	-1.016	-1.010	-1.049	-1.085	-1.120
188	RPPos	LacCer(d42:2)	6.36	1.086	1.012	-1.031	-1.113	1.116	1.088	1.078	1.368	1.256
189	RPPos	LPC(14:0)	1.56	1.015	-1.010	-1.059	-1.008	-1.189	1.145	-1.073	-1.322	-1.612
190	RPNeg	LPC(14:0)	1.63	1.119	-1.135	-1.064	-1.005	-1.194	1.061	-1.129	-1.400	-1.585
191	RPPos	LPC(15:0)	1.79	-1.081	-1.060	-1.011	-1.057	-1.006	1.159	1.005	-1.152	-1.010
192	RPNeg	LPC(15:0)	1.85	-1.012	1.008	1.004	1.015	-1.100	1.198	-1.054	-1.206	-1.056
193	RPPos	LPC(15:1)	1.58	1.049	1.028	-1.121	-1.023	-1.357	1.098	-1.076	-1.456	-1.746
194	RPPos	LPC(16:0)	2.01	-1.036	-1.121	-1.042	-1.015	-1.080	1.060	-1.090	-1.170	-1.065
195	RPNeg	LPC(16:0)	1.99	-1.018	-1.035	1.064	1.047	1.010	1.220	-1.146	-1.114	1.025
196	RPPos	LPC(16:1)	1.68	-1.009	-1.085	-1.085	-1.025	-1.131	-1.049	-1.095	-1.423	-1.632
197	RPNeg	LPC(16:1)	1.74	1.075	-1.126	-1.027	1.007	-1.130	1.024	-1.136	-1.314	-1.421
198	RPPos	LPC(17:0)	2.12	1.032	-1.130	-1.030	1.021	-1.178	-1.007	-1.075	-1.196	-1.205
199	RPNeg	LPC(17:0)	2.26	1.119	-1.133	-1.022	1.004	1.033	1.087	-1.063	-1.071	1.039
200	RPPos	LPC(17:1)	1.90	-1.001	-1.208	-1.085	-1.086	-1.317	-1.057	-1.147	-1.690	-1.699
201	RPPos	LPC(18:0)	2.33	1.050	1.043	-1.044	1.098	1.068	1.092	-1.022	-1.015	1.055
202	RPNeg	LPC(18:0)	2.40	1.048	-1.017	-1.056	1.113	1.039	-1.032	-1.150	-1.093	1.051
203	RPPos	LPC(18:1)	2.08	-1.025	-1.203	-1.133	-1.012	-1.328	-1.020	-1.126	-1.416	-1.288
204	RPPos	LPC(18:2)	1.80	-1.062	-1.207	-1.090	-1.060	-1.345	1.001	-1.099	-1.702	-1.307
205	RPNeg	LPC(18:2)	1.87	1.011	-1.257	-1.108	-1.043	-1.322	1.032	-1.136	-1.609	-1.329
206	RPPos	LPC(18:3)	1.57	1.072	-1.107	-1.111	-1.010	-1.161	1.074	-1.061	-1.621	-1.879
207	RPPos	LPC(18:4)	1.35	-1.015	-1.364	1.012	1.013	-1.639	-1.106	-1.187	-1.850	-2.217
208	RPPos	LPC(19:0)	2.47	1.416	1.066	-1.306	1.319	-1.105	-1.250	1.882	1.776	1.639
209	RPNeg	LPC(19:0)	2.49	1.068	-1.054	1.034	1.125	-1.054	-1.056	-1.004	1.034	-1.046
210	RPNeg	LPC(19:0)	2.54	1.146	-1.033	-1.043	-1.071	1.165	1.151	1.159	1.081	1.154
211	RPNeg	LPC(19:1)	2.30	1.044	-1.311	-1.054	1.029	-1.055	-1.043	-1.031	-1.247	-1.092
212	RPPos	LPC(19:1)	2.23	1.010	-1.179	-1.105	-1.001	-1.141	-1.035	-1.023	-1.317	-1.274
213	RPPos	LPC(19:4)	1.57	1.042	1.135	-1.290	-1.027	-1.087	-1.042	-1.041	-1.194	-1.293

214	RPPos	LPC(20:0)	2.65	1.143	-1.175	-1.224	-1.027	1.030	-1.040	1.196	1.077	-1.084
215	RPPos	LPC(20:1)	2.36	1.073	-1.047	-1.102	-1.087	-1.108	-1.026	1.008	-1.324	1.011
216	RPPos	LPC(20:3)	1.94	1.061	-1.303	-1.277	1.048	-1.367	-1.296	-1.007	-1.725	-1.953
217	RPNeg	LPC(20:3)	2.02	1.217	-1.292	-1.254	-1.009	-1.314	-1.242	-1.053	-1.604	-1.975
218	RPPos	LPC(20:4)	1.77	1.025	1.027	1.005	1.100	-1.006	1.051	1.041	-1.110	1.103
219	RPNeg	LPC(20:4)	1.84	1.086	-1.008	1.005	1.045	-1.007	1.018	-1.015	-1.051	1.051
220	RPPos	LPC(20:5)	1.53	1.059	-1.254	1.043	1.005	-1.420	1.188	-1.082	-1.717	-1.829
221	RPNeg	LPC(20:5)	1.59	1.100	-1.504	1.108	1.008	-1.449	1.128	-1.090	-1.750	-1.910
222	RPPos	LPC(21:0)	2.73	1.222	-1.033	-1.521	1.258	1.064	-1.410	2.385	1.691	1.087
223	RPPos	LPC(22:0)	3.03	-1.041	-1.083	-1.067	1.031	1.009	1.023	-1.044	-1.058	-1.066
224	RPNeg	LPC(22:0)	3.10	1.099	-1.012	-1.074	-1.008	1.000	1.015	1.007	-1.045	-1.052
225	RPPos	LPC(22:1)	2.66	1.194	-1.104	-1.318	1.168	-1.045	-1.097	1.375	1.042	-1.097
226	RPPos	LPC(22:4)	2.08	1.039	-1.288	-1.092	-1.004	-1.449	1.008	-1.082	-1.580	-1.152
227	RPNeg	LPC(22:4)	2.15	1.143	-1.237	-1.044	-1.004	-1.335	1.100	-1.074	-1.529	-1.057
228	RPPos	LPC(22:5)	1.84	1.112	-1.204	-1.068	1.037	-1.345	1.068	1.014	-1.514	-1.192
229	RPPos	LPC(22:5)	1.96	1.176	-1.379	-1.340	-1.021	-1.452	-1.234	-1.122	-1.895	-1.504
230	RPNeg	LPC(22:5)	2.03	1.310	-1.157	-1.031	1.068	-1.426	-1.122	1.012	-1.525	-1.389
231	RPNeg	LPC(22:5)	1.92	1.204	-1.261	1.023	1.123	-1.266	1.027	-1.012	-1.515	-1.152
232	RPPos	LPC(22:6)	1.72	1.166	-1.269	-1.048	-1.006	-1.287	1.041	1.012	-1.521	-1.189
233	RPNeg	LPC(24:0)	2.77	1.028	-1.388	-1.230	-1.043	-1.205	1.016	-1.152	-1.143	-1.151
234	RPPos	LPC(24:0)	3.56	1.068	-1.042	-1.063	-1.022	1.046	1.030	-1.052	-1.039	-1.046
235	RPPos	LPC(24:1)	3.02	-1.032	-1.060	-1.066	1.051	-1.025	-1.053	-1.023	-1.148	-1.134
236	RPPos	LPC(24:2)	2.71	1.213	-1.115	-1.559	1.119	-1.090	-1.714	2.200	1.391	-1.054
237	RPPos	LPE(16:0)	2.05	-1.074	-1.101	-1.165	-1.028	-1.018	1.092	-1.147	-1.253	-1.188
238	RPNeg	LPE(16:0)	2.12	-1.081	-1.020	-1.072	-1.048	1.061	1.047	-1.182	-1.250	-1.192
239	RPPos	LPE(17:0)	2.23	-1.131	-1.180	-1.199	-1.012	-1.103	1.014	-1.131	-1.275	-1.178
240	RPPos	LPE(18:0)	2.37	-1.010	-1.026	1.007	-1.057	-1.060	1.128	-1.194	-1.208	-1.053
241	RPNeg	LPE(18:0)	2.08	-1.006	-1.122	-1.052	-1.044	-1.054	1.006	-1.061	-1.164	1.014
242	RPPos	LPE(18:1)	2.12	-1.108	-1.206	-1.175	-1.166	-1.351	-1.085	-1.113	-1.523	-1.290
243	RPPos	LPE(18:2)	1.78	1.041	-1.074	-1.172	-1.402	-1.143	-1.038	-1.507	-1.734	-1.212
244	RPPos	LPE(18:2)	1.84	-1.196	-1.076	-1.177	-1.228	-1.235	-1.021	-1.310	-1.804	-1.326
245	RPPos	LPE(18:2)	1.36	1.142	1.014	1.042	1.108	1.022	1.235	1.028	-1.093	-1.437
246	RPPos	LPE(18:3)	1.76	-1.310	-1.163	-1.468	-1.065	1.297	-1.766	-1.052	1.081	-3.045
247	RPNeg	LPE(20:0)	2.40	1.044	-1.088	-1.051	1.136	1.024	1.074	-1.095	-1.128	-1.036
248	RPPos	LPE(20:1)	2.39	-1.034	1.051	1.054	1.068	-1.027	1.111	-1.132	-1.711	-1.578
249	RPPos	LPE(20:4)	1.82	1.038	1.050	-1.088	1.026	1.101	1.174	1.080	1.025	1.009
250	RPNeg	LPE(20:4)	1.89	1.051	-1.002	-1.262	1.163	1.053	1.088	1.132	1.070	1.013

251	RPPos	LPE(21:3)	2.45	1.680	-1.026	-1.646	1.303	-1.003	-1.470	2.594	2.081	2.393
252	RPNeg	LPE(22:6)	1.83	1.000	-1.104	-1.162	1.179	1.022	1.135	1.149	-1.046	1.067
253	RPPos	LPE(22:6)	1.76	1.074	-1.010	-1.070	1.198	-1.015	1.030	1.198	1.005	-1.048
254	RPNeg	LPE(O-18:0)	2.55	-1.157	-1.231	1.163	-1.014	1.058	-1.062	1.132	-1.018	-1.196
255	RPPos	LPE(O-18:2)	2.24	-1.302	-1.287	1.187	1.071	-1.089	1.106	-1.046	-1.077	-1.777
256	RPNeg	LPE(O-18:2)	2.31	-1.212	-1.282	1.334	1.006	1.014	-1.100	-1.079	-1.141	-1.658
257	RPPos	LPE(O-18:2)	2.23	-1.283	-1.295	1.180	1.068	-1.070	1.114	-1.040	-1.050	-1.750
258	RPPos	LPI(18:0)	2.19	1.098	-1.032	-1.045	1.383	1.014	1.297	1.113	1.232	1.190
259	RPPos	LPS(18:0)	2.20	-1.283	-1.234	1.397	-1.164	-1.114	1.138	-1.056	-1.366	-1.682
260	RPPos	LPS(18:1)	1.90	-1.012	-1.377	-1.008	1.117	1.090	-1.118	-1.135	-1.370	-1.836
261	RPPos	LPS(18:2)	1.59	1.037	-1.405	-1.040	1.464	-1.001	-1.249	-1.100	-1.359	-1.961
262	RPPos	LPS(20:4)	1.58	1.013	-1.164	-1.110	1.258	1.094	-1.245	-1.066	-1.308	-1.941
263	RPPos	LPS(20:5)	1.34	1.037	-1.151	-1.114	1.321	1.147	-1.232	-1.090	-1.115	-2.447
264	RPPos	LPS(22:4)	1.91	-1.022	-1.162	-1.083	1.506	1.314	-1.004	-1.127	-1.295	-2.310
265	RPPos	LPS(22:5)	1.66	1.023	-1.222	1.011	1.278	1.203	1.016	-1.101	-1.337	-2.339
266	RPPos	LPS(22:6)	1.53	-1.089	-1.136	-1.074	1.000	1.059	-1.747	-1.104	-1.379	-1.860
267	RPPos	PA(33:0)	5.40	-1.774	-1.347	-1.433	-1.368	-1.634	1.132	-2.612	-1.377	-1.485
268	RPPos	PA(33:1)	4.51	1.158	-1.010	1.134	1.072	1.449	1.689	-1.155	2.034	1.655
269	RPPos	PA(35:0)	6.31	-2.225	-1.147	-1.130	-1.482	-1.816	1.057	-2.351	-1.443	-1.196
270	RPPos	PA(35:0)	6.04	-1.334	-1.130	-1.113	-1.104	-1.674	1.174	-1.551	-1.977	-1.477
271	RPPos	PA(35:1)	5.47	-1.006	-1.214	1.104	-1.111	-1.314	1.201	-1.472	-1.131	-1.159
272	RPPos	PA(42:5)	4.79	-1.387	-1.427	-1.570	-1.380	-1.208	-1.046	-1.090	-1.349	-2.035
273	RPPos	PC(23:0)	3.16	1.008	-1.060	-1.095	-1.023	-1.140	1.005	1.057	-1.003	1.040
274	RPPos	PC(23:0)	3.28	-1.028	-1.004	1.030	1.056	1.048	1.028	-1.072	-1.073	-1.046
275	RPNeg	PC(23:0)	3.34	-1.002	-1.064	1.007	-1.070	-1.026	1.056	-1.125	-1.104	1.009
276	RPPos	PC(28:0)	3.43	-1.163	-1.087	-1.325	-1.040	-1.268	-1.305	-1.027	-1.172	-1.558
277	RPPos	PC(29:0)	3.70	-1.086	-1.086	-1.322	1.029	-1.201	-1.041	-1.055	-1.405	-1.515
278	RPPos	PC(30:0)	4.00	-1.194	-1.194	-1.278	-1.184	-1.455	-1.311	-1.166	-1.722	-1.601
279	RPNeg	PC(30:0)>PC(14:0_16:0)	4.06	1.015	-1.259	-1.221	-1.115	-1.713	-1.223	-1.177	-1.831	-1.966
280	RPPos	PC(31:0)	4.34	-1.029	-1.159	-1.155	-1.047	-1.160	-1.034	-1.062	-1.357	-1.256
281	RPPos	PC(31:0) or PE(34:0)	5.83	-1.517	-1.702	-1.163	-1.358	-2.051	-1.048	-1.607	-2.648	-1.489
282	RPPos	PC(31:1) or PE(34:1)	4.00	-1.582	-1.163	-1.505	-1.694	-2.184	-1.751	-1.637	-2.354	-2.598
283	RPPos	PC(31:1)	3.79	-1.028	-1.047	-1.302	-1.008	-1.450	-1.240	-1.205	-1.469	-1.957
284	RPPos	PC(31:2) or PE(34:2)	3.42	-1.266	-1.143	-1.283	-1.301	-1.761	-1.356	-1.133	-1.469	-1.726
285	RPPos	PC(31:2) or PE(34:2)	3.68	1.068	-1.201	-1.212	-1.277	-1.280	-1.212	-1.118	-1.500	-1.909
286	RPPos	PC(32:0)	4.71	-1.172	-1.321	-1.227	-1.059	-1.391	-1.145	-1.122	-1.646	-1.335

287	RPPos	PC(32:0)	4.02	-1.059	-1.155	-1.285	1.199	-1.317	-1.036	-1.391	-1.383	-1.291
288	RPNeg	PC(32:0)>PC(16:0/16:0)	4.76	-1.074	-1.283	-1.133	-1.111	-1.401	-1.136	-1.148	-1.555	-1.344
289	RPNeg	PC(32:0)>PC(16:0/16:0)	4.07	1.139	-1.042	1.026	-1.189	-1.204	1.008	1.311	-1.309	-1.369
290	RPPos	PC(32:1)	4.08	-1.036	-1.064	-1.174	1.006	-1.258	-1.159	-1.100	-1.620	-1.890
291	RPPos	PC(32:1)	3.54	1.026	-1.028	-1.258	-1.027	-1.235	-1.347	-1.064	-1.215	-1.914
292	RPNeg	PC(32:1)>PC(14:0_18:1) and PC(16:0_16:1)	4.14	-1.002	-1.240	-1.228	-1.021	-1.371	-1.176	-1.032	-1.504	-2.059
293	RPPos	PC(32:2)	3.61	-1.063	-1.196	-1.177	-1.126	-1.435	-1.073	-1.136	-1.600	-1.584
294	RPNeg	PC(32:2)>PC(14:0_18:2)	3.68	-1.062	-1.238	-1.072	-1.096	-1.436	1.007	-1.045	-1.698	-1.654
295	RPPos	PC(32:3)	3.53	1.043	1.274	-1.203	1.056	1.074	-1.310	1.018	1.409	-1.324
296	RPPos	PC(33:0)	5.11	-1.066	-1.222	-1.220	-1.052	-1.339	-1.102	-1.235	-1.586	-1.309
297	RPPos	PC(33:0)	4.95	-1.055	-1.181	-1.118	-1.247	-1.309	-1.128	-1.122	-1.738	-1.603
298	RPPos	PC(33:0)	4.31	1.225	1.129	-1.031	1.145	1.148	1.054	1.085	1.127	-1.365
299	RPPos	PC(33:1)	4.43	1.059	-1.024	-1.024	1.015	-1.190	-1.004	-1.069	-1.312	-1.445
300	RPPos	PC(33:1)	3.82	-1.063	-1.007	-1.038	-1.051	-1.032	-1.008	-1.089	1.012	-1.088
301	RPNeg	PC(33:1)	4.46	1.041	-1.066	-1.143	-1.011	-1.239	1.044	-1.107	-1.342	-1.531
302	RPPos	PC(33:2)	4.39	-1.298	-1.129	-1.324	1.040	-1.826	-1.190	-1.184	-1.903	-1.529
303	RPPos	PC(33:3)	3.60	-1.103	-1.151	-1.259	-1.135	-1.375	-1.100	-1.241	-2.008	-1.874
304	RPPos	PC(34:0)	5.55	-1.104	-1.269	-1.151	-1.039	-1.401	-1.005	-1.089	-1.591	-1.006
305	RPNeg	PC(34:0)>PC(16:0_18:0)	5.60	1.015	-1.210	-1.133	-1.051	-1.342	1.032	-1.095	-1.520	-1.115
306	RPPos	PC(34:1)	4.79	1.052	1.005	-1.013	1.025	-1.073	-1.040	-1.033	-1.217	-1.232
307	RPNeg	PC(34:1)>PC(16:0_18:1)	4.84	1.092	1.012	-1.057	1.024	-1.104	1.003	-1.035	-1.104	-1.095
308	RPPos	PC(34:2)	3.65	-1.211	-1.145	-1.223	1.145	-1.055	1.215	-1.006	1.131	-1.265
309	RPPos	PC(34:2)	4.23	-1.054	-1.057	-1.075	-1.111	-1.088	-1.044	-1.125	-1.234	-1.044
310	RPPos	PC(34:2)	4.46	-1.104	-1.084	1.220	1.163	-1.005	1.200	-1.369	-2.312	-1.053
311	RPNeg	PC(34:2)>PC(16:0_18:2)	4.29	-1.046	-1.035	-1.049	-1.135	-1.186	-1.025	-1.171	-1.268	-1.048
312	RPNeg	PC(34:2)>PC(16:0_18:2)	4.29	-1.007	-1.130	1.096	-1.041	-1.020	-1.051	-1.215	-1.319	-1.168
313	RPPos	PC(34:3)	3.70	-1.110	1.030	-1.019	-1.193	-1.331	-1.178	-1.181	-1.609	-1.274
314	RPPos	PC(34:3)	4.46	-1.177	-1.496	-1.218	-1.209	-1.137	-1.097	1.434	1.078	-1.275
315	RPNeg	PC(34:3)>PC(16:0_18:3)	3.75	-1.065	-1.073	1.047	-1.196	-1.213	-1.110	-1.244	-1.599	-1.082
316	RPNeg	PC(34:3)>PC(16:0_18:3)	3.93	-1.019	-1.246	-1.211	-1.053	-1.232	-1.341	-1.191	-1.733	-1.872
317	RPPos	PC(34:4)	3.53	1.027	-1.028	-1.249	-1.027	-1.236	-1.349	-1.064	-1.212	-1.881
318	RPPos	PC(34:4)	3.53	1.025	-1.033	-1.265	-1.028	-1.239	-1.351	-1.060	-1.211	-1.910
319	RPPos	PC(34:4)	3.36	1.027	-1.053	1.027	-1.102	-1.106	-1.274	-1.168	-1.135	-1.212
320	RPPos	PC(34:5)	3.23	-1.108	-1.289	-1.357	-1.086	-1.523	-1.289	-1.195	-1.636	-2.622
321	RPNeg	PC(34:5)	4.13	1.024	-1.007	-1.620	1.100	-1.541	-1.285	1.740	-1.359	-1.043
322	RPPos	PC(35:0)	5.99	-1.022	-1.099	-1.030	-1.051	-1.199	-1.089	-1.065	-1.230	1.012

323	RPPos	PC(35:0)	5.82	-1.212	-1.061	-1.132	-1.046	-1.198	-1.218	-1.082	-1.270	-1.143
324	RPPos	PC(35:0)	5.07	1.002	-1.008	-1.222	-1.184	-1.087	-1.293	-1.133	-1.173	-1.457
325	RPPos	PC(35:1)	5.21	-1.060	-1.020	-1.112	1.124	-1.077	-1.058	-1.099	-1.038	-1.194
326	RPPos	PC(35:1)	4.42	1.294	-1.015	1.227	-1.109	1.079	1.307	-1.122	1.186	1.112
327	RPNeg	PC(35:1)>PC(17:0_18:1)	5.25	1.150	1.124	-1.015	-1.012	-1.051	1.011	-1.050	-1.096	-1.234
328	RPPos	PC(35:2)	4.60	-1.052	-1.115	-1.045	-1.076	-1.156	1.052	-1.198	-1.376	-1.196
329	RPPos	PC(35:2)	4.47	1.039	1.022	1.039	-1.052	-1.160	-1.034	-1.199	-1.407	-1.469
330	RPPos	PC(35:2)	6.02	-1.225	-1.014	-1.587	-1.320	-1.676	-1.319	-1.544	-2.342	-1.472
331	RPPos	PC(35:2)	5.17	1.049	-1.020	1.012	-1.301	-1.713	-1.291	-1.407	-1.616	-1.579
332	RPNeg	PC(35:2)>PC(17:0_18:2)	4.66	-1.044	-1.022	1.009	-1.177	-1.213	1.028	-1.191	-1.352	-1.197
333	RPNeg	PC(35:2)>PC(17:0_18:2)	4.52	-1.006	-1.116	-1.039	-1.044	-1.229	1.045	-1.211	-1.662	-1.563
334	RPPos	PC(35:3)	3.99	-1.163	-1.207	-1.119	-1.189	-1.358	-1.252	-1.074	-1.574	-1.871
335	RPPos	PC(35:4)	3.82	-1.061	-1.013	-1.031	-1.056	-1.034	-1.005	-1.084	1.018	-1.072
336	RPPos	PC(36:0)	6.39	-1.014	-1.024	-1.090	-1.099	-1.071	-1.111	-1.120	-1.016	1.051
337	RPPos	PC(36:0)	6.38	-1.014	-1.015	-1.092	-1.105	-1.062	-1.117	-1.118	-1.030	-1.033
338	RPNeg	PC(36:0)>PC(18:0/18:0)	6.45	1.032	-1.042	-1.112	-1.082	-1.088	-1.017	-1.103	-1.171	1.060
339	RPPos	PC(36:1)	5.64	-1.024	1.053	-1.030	1.042	1.047	-1.079	-1.049	1.086	-1.139
340	RPNeg	PC(36:1)>PC(16:0_20:1) and PC(18:0_18:1)	5.69	1.067	1.066	-1.055	-1.048	1.018	1.002	-1.038	1.055	-1.124
341	RPPos	PC(36:2)	5.01	1.010	-1.005	-1.022	-1.086	-1.070	1.038	-1.146	-1.184	-1.080
342	RPPos	PC(36:2)	5.08	1.084	1.019	1.217	-1.030	1.094	1.040	-1.140	1.047	-1.013
343	RPPos	PC(36:2)	5.06	-1.047	-1.078	-1.059	-1.084	1.002	1.076	-1.073	1.044	1.036
344	RPNeg	PC(36:2)>PC(18:0_18:2)	5.06	1.031	-1.016	-1.031	-1.084	-1.061	-1.012	-1.099	-1.159	-1.082
345	RPNeg	PC(36:2)>PC(18:1/18:1)	4.89	-1.005	-1.894	1.069	-1.071	-1.509	-1.038	-1.110	-1.259	-1.343
346	RPPos	PC(36:3)	4.43	1.080	1.119	1.098	1.064	-1.139	-1.096	1.012	-1.001	-1.222
347	RPPos	PC(36:3)	4.30	-1.114	-1.203	-1.121	-1.231	-1.457	-1.144	-1.432	-1.942	-1.307
348	RPNeg	PC(36:3)>PC(16:0_20:3)	4.47	1.204	-1.100	-1.288	1.208	3.082	1.422	1.374	1.575	1.188
349	RPNeg	PC(36:3)>PC(18:1_18:2)	4.34	-1.186	-1.396	-1.152	-1.304	-1.648	-1.263	-1.320	-2.326	-1.381
350	RPPos	PC(36:4)	4.14	1.024	1.054	-1.054	-1.029	1.145	-1.053	-1.002	1.168	1.088
351	RPPos	PC(36:4)	3.81	-1.331	-1.300	-1.210	-1.371	-1.855	-1.068	-1.493	-3.063	-1.620
352	RPNeg	PC(36:4)>PC(16:0_20:4)	4.19	1.031	1.016	-1.086	-1.068	1.082	-1.004	-1.055	1.082	1.016
353	RPNeg	PC(36:4)>PC(18:2/18:2)	3.87	-1.196	-1.269	-1.181	-1.274	-1.605	-1.036	-1.386	-2.532	-1.464
354	RPPos	PC(36:5)	3.74	-1.153	-1.190	-1.358	-1.159	-1.317	-1.191	-1.287	-1.606	-1.654
355	RPPos	PC(36:5)	3.45	-1.297	-1.108	-1.227	-1.309	-1.307	-1.041	-1.362	-1.908	-1.400
356	RPNeg	PC(36:5)>PC(16:0_20:5)	3.81	-1.059	-1.145	-1.286	-1.155	-1.263	-1.161	-1.207	-1.370	-1.559
357	RPNeg	PC(36:5)>PC(16:1_20:4)	3.51	-1.234	-1.104	-1.151	-1.347	-1.430	-1.041	-1.529	-1.991	-1.379
358	RPNeg	PC(36:5)>PC(16:1_20:4)	3.67	-1.001	-1.051	-1.117	-1.037	-1.037	-1.231	-1.047	-1.134	-1.433

359	RPPos	PC(36:6)	3.41	1.182	-1.058	-1.140	1.127	-1.255	-1.167	1.047	-1.325	-1.904
360	RPPos	PC(36:6)	3.29	-1.239	-1.037	-1.332	-1.103	-1.511	-1.404	-1.268	-2.067	-1.751
361	RPPos	PC(36:6) or PE(39:6)	3.70	-1.195	-1.106	-1.706	-1.033	-1.667	-1.384	-1.040	-1.103	-1.221
362	RPPos	PC(37:0)	4.63	-1.078	1.058	1.044	-1.115	1.216	1.082	1.040	1.191	1.371
363	RPPos	PC(37:1)	6.08	1.045	1.111	1.015	1.036	1.063	-1.027	1.008	1.168	1.027
364	RPPos	PC(37:1)	5.91	-1.237	-1.283	-1.218	1.014	1.002	-1.146	-1.037	-1.120	-1.512
365	RPPos	PC(37:2)	5.44	-1.011	1.082	-1.003	-1.066	-1.060	1.016	-1.084	-1.064	-1.197
366	RPPos	PC(37:2)	6.71	-1.978	-1.437	-1.392	-2.309	-1.464	-2.120	-2.147	-2.225	-2.193
367	RPNeg	PC(37:2)>PC(18:2_19:0)	5.49	1.017	1.134	1.007	-1.049	-1.016	1.113	-1.169	-1.120	-1.068
368	RPNeg	PC(37:2)>PC(18:2_19:0)	5.32	1.176	1.039	-1.057	-1.267	-1.103	-1.133	-1.132	-1.079	-1.029
369	RPPos	PC(37:3)	4.62	-1.082	-1.172	-1.224	-1.058	-1.284	-1.253	-1.103	-1.389	-1.395
370	RPPos	PC(37:3)	4.80	1.096	1.212	-1.025	-1.026	-1.057	-1.313	1.007	1.034	-1.477
371	RPPos	PC(37:4)	4.49	1.098	1.266	-1.048	1.121	1.216	1.043	-1.087	1.294	-1.015
372	RPPos	PC(37:4)	4.36	1.092	1.033	-1.095	1.064	1.139	-1.103	1.105	1.163	-1.247
373	RPPos	PC(37:4)	4.09	-1.000	1.027	-1.111	-1.072	-1.203	-1.157	-1.371	-1.326	-1.162
374	RPNeg	PC(37:4)>PC(17:0_20:4)	4.55	1.107	1.146	-1.011	1.024	1.244	1.024	-1.033	1.334	-1.036
375	RPNeg	PC(37:4)>PC(17:0_20:4)	4.43	1.080	1.089	-1.004	1.003	1.139	-1.003	-1.030	1.098	-1.277
376	RPPos	PC(37:5)	4.61	1.003	1.051	-1.074	1.025	1.124	-1.018	-1.006	1.053	-1.028
377	RPPos	PC(37:5)	5.26	1.032	1.025	-1.123	1.033	1.149	-1.024	1.026	1.126	1.320
378	RPPos	PC(37:6)	3.68	1.069	1.049	1.002	1.141	-1.091	1.036	1.023	-1.014	-1.094
379	RPPos	PC(37:6)	4.13	1.004	1.076	-1.050	-1.085	1.152	1.045	-1.037	-1.104	1.069
380	RPPos	PC(37:7) or PE(40:7)	3.40	1.096	-1.062	-1.132	1.171	-1.151	1.113	1.013	-1.010	-1.702
381	RPNeg	PC(37:8)	3.74	1.116	1.251	1.011	1.803	-1.085	1.297	-1.151	-1.176	-1.022
382	RPNeg	PC(37:8)	3.83	1.062	1.147	-1.121	1.096	-1.438	-1.216	1.063	-1.145	1.322
383	RPPos	PC(38:0)	6.43	1.031	-1.105	-1.140	-1.110	-1.053	-1.217	1.009	-1.206	-1.301
384	RPPos	PC(38:0)	6.78	1.051	1.082	1.006	1.019	-1.056	1.087	1.007	-1.090	-1.002
385	RPPos	PC(38:1)	6.39	1.006	1.025	-1.070	-1.110	1.016	1.049	-1.109	-1.068	-1.083
386	RPPos	PC(38:1)	5.68	-1.085	1.055	-1.343	1.026	1.147	-1.129	1.020	1.185	-1.569
387	RPPos	PC(38:1)	6.58	-1.090	1.022	-1.202	-1.134	-1.121	-1.107	-1.049	-1.086	-1.204
388	RPPos	PC(38:2)	5.75	1.048	1.018	-1.023	1.030	-1.018	1.005	-1.088	1.008	-1.326
389	RPPos	PC(38:2)	5.05	1.059	-1.029	-1.153	-1.027	1.110	-1.220	-1.228	1.002	-2.011
390	RPNeg	PC(38:2)>PC(18:0_20:2)	5.80	1.078	1.058	1.001	-1.025	-1.013	1.085	-1.106	-1.070	-1.406
391	RPNeg	PC(38:2)>PC(18:1_20:1)	5.67	1.019	1.010	-1.083	-1.105	-1.098	-1.029	-1.087	-1.242	-1.156
392	RPPos	PC(38:3)	5.22	1.080	1.028	-1.209	1.124	1.158	-1.247	1.035	1.189	-1.326
393	RPPos	PC(38:3)	5.31	1.243	1.147	-1.037	-1.061	1.088	-1.559	1.022	1.069	-4.308
394	RPPos	PC(38:3)	5.01	1.013	-1.121	-1.061	-1.132	-1.075	-1.156	-1.036	-1.423	-1.284
395	RPPos	PC(38:3)	5.48	1.072	1.028	-1.190	1.032	1.068	-1.527	-1.066	1.228	-1.521

396	RPNeg	PC(38:3)>PC(18:0_20:3)	5.37	1.105	1.066	-1.233	-1.353	1.041	-1.249	-1.092	1.121	-1.669
397	RPNeg	PC(38:3)>PC(18:0_20:3)	5.07	-1.134	-1.160	-1.196	-1.062	-1.156	1.165	-1.077	-1.362	-1.227
398	RPNeg	PC(38:3)>PC(18:0_20:3)	5.27	1.137	1.232	1.029	1.032	1.111	-1.103	-1.061	1.128	-1.198
399	RPNeg	PC(38:3)>PC(18:0_20:3)	5.53	1.061	1.062	-1.251	1.024	1.165	-1.182	-1.044	1.125	-1.706
400	RPPos	PC(38:4)	4.89	1.075	1.109	1.024	1.049	1.164	1.062	-1.023	1.164	-1.006
401	RPPos	PC(38:4)	4.38	1.148	1.063	1.019	-1.356	1.090	-1.061	-1.259	1.054	1.378
402	RPPos	PC(38:4)	4.07	1.067	-1.025	-1.160	1.048	1.005	-1.115	-1.026	1.139	1.034
403	RPNeg	PC(38:4)>PC(18:0_20:4)	4.94	1.127	1.093	-1.014	-1.028	1.144	1.037	-1.040	1.199	-1.023
404	RPNeg	PC(38:4)>PC(18:0_20:4)	4.68	1.024	-1.081	-1.398	1.034	-1.073	-1.380	-1.190	-1.151	-1.302
405	RPPos	PC(38:5)	4.18	1.082	1.098	-1.060	-1.069	1.083	-1.117	-1.043	1.077	-1.038
406	RPPos	PC(38:5)	4.42	1.069	-1.061	-1.096	1.017	-1.060	-1.076	-1.115	-1.110	-1.381
407	RPPos	PC(38:5)	3.61	1.032	1.099	1.084	-1.021	1.073	1.086	-1.063	1.181	1.112
408	RPNeg	PC(38:5)>PC(16:0_22:5)	3.67	-1.024	1.060	-1.085	1.087	1.372	1.414	-1.283	1.377	1.047
409	RPNeg	PC(38:5)>PC(16:0_22:5) and PC(18:1_20:4)	4.24	1.110	-1.007	-1.079	-1.013	1.013	-1.148	1.014	1.053	-1.081
410	RPNeg	PC(38:5)>PC(16:0_22:5) and PC(18:1_20:4)	4.46	1.039	-1.096	-1.160	-1.021	-1.082	1.034	-1.122	-1.099	-1.483
411	RPPos	PC(38:6)	3.72	-1.103	1.010	-1.046	-1.007	-1.030	1.056	-1.264	-1.204	-1.157
412	RPPos	PC(38:6)	3.98	1.134	1.040	-1.062	1.067	1.062	1.008	1.054	1.204	1.037
413	RPPos	PC(38:6)	3.47	1.013	1.124	-1.104	1.131	1.099	1.118	-1.136	1.450	-1.073
414	RPNeg	PC(38:6)>PC(16:0_22:6) and PC(18:0_20:6)	4.03	1.163	1.012	1.028	1.054	1.036	1.047	1.004	1.046	1.001
415	RPNeg	PC(38:6)>PC(16:0_22:6) and PC(18:0_20:6)	4.03	1.308	1.026	-1.008	1.047	1.142	1.009	1.144	1.270	1.020
416	RPNeg	PC(38:6)>PC(18:2_20:4)	3.78	1.019	-1.019	1.002	-1.080	-1.100	-1.030	-1.107	-1.176	-1.110
417	RPPos	PC(38:7)	3.32	-1.127	-1.032	-1.002	-1.335	-1.888	-1.497	-1.968	-4.237	-1.918
418	RPPos	PC(38:7)	3.38	-1.347	-1.322	-1.148	-1.325	-1.537	-1.085	-1.393	-1.930	-1.704
419	RPPos	PC(38:7)	3.65	1.174	1.034	1.432	-1.106	-1.107	-1.107	-1.061	-1.408	-1.133
420	RPPos	PC(38:7)	3.49	-1.081	-1.113	-1.191	1.016	-1.183	-1.231	1.103	1.046	-1.067
421	RPPos	PC(39:1)	6.68	-1.040	1.010	1.132	-1.029	-1.102	1.036	-1.129	-1.045	-1.032
422	RPPos	PC(39:3)	6.29	1.288	-1.243	1.004	1.099	-1.106	1.112	-1.018	-1.164	1.225
423	RPPos	PC(39:3)	5.65	1.024	1.099	-1.013	1.005	-1.057	-1.235	1.068	-1.070	-1.226
424	RPPos	PC(39:3)	6.24	1.184	1.226	-1.009	1.140	1.006	1.227	-1.100	-1.025	1.217
425	RPPos	PC(39:4)	5.31	1.095	1.155	-1.109	1.041	1.245	1.041	1.012	1.314	-1.021
426	RPPos	PC(39:4)	5.14	1.102	1.147	-1.140	1.003	1.296	-1.090	-1.065	1.344	-1.055
427	RPPos	PC(39:4)	4.34	1.062	1.173	-1.253	1.040	1.016	-1.098	-1.351	1.034	-1.381
428	RPPos	PC(39:4)	4.34	1.123	1.158	-1.109	1.169	1.086	-1.129	1.156	1.382	-1.076
429	RPNeg	PC(39:4)>PC(19:0_20:4)	5.37	1.142	1.228	-1.043	-1.010	1.230	1.123	-1.008	1.292	1.152

430	RPNeg	PC(39:4)>PC(19:0_20:4)	5.20	1.142	1.152	-1.070	1.030	1.290	1.109	-1.002	1.341	1.027
431	RPPos	PC(39:5)	3.93	-1.039	-1.111	-1.253	-1.017	-1.108	-1.059	-1.048	-1.174	-1.533
432	RPPos	PC(39:5)	4.51	1.097	1.126	-1.159	1.047	1.092	-1.009	-1.014	1.116	-1.205
433	RPPos	PC(39:6)	4.19	1.101	1.026	1.133	1.054	1.181	-1.000	1.043	1.097	-1.080
434	RPPos	PC(39:6)	4.32	1.234	1.191	1.068	1.116	1.277	1.144	1.114	1.504	1.152
435	RPPos	PC(39:6)	3.99	-1.179	-1.161	-1.655	1.020	1.126	-1.235	-1.108	1.286	-1.423
436	RPPos	PC(39:6)	3.62	-1.010	-1.054	-1.212	-1.049	-1.072	-1.249	-1.046	-1.165	-1.355
437	RPNeg	PC(39:6)>PC(17:0_22:6)	4.24	1.265	1.069	-1.017	1.030	1.085	1.203	1.126	1.113	-1.488
438	RPNeg	PC(39:6)>PC(17:0_22:6)	4.37	1.098	-1.066	1.124	1.002	1.114	1.160	1.023	1.191	1.042
439	RPPos	PC(39:7)	3.76	1.006	1.046	1.172	1.008	-1.062	-1.118	-1.034	-1.031	-1.467
440	RPPos	PC(40:0)	7.06	-1.159	-1.168	-1.176	-1.065	-1.181	-1.237	-1.075	-1.449	-1.131
441	RPNeg	PC(40:1)>PC(18:1_22:0)	6.88	-1.083	-1.286	-1.299	1.073	-1.124	-1.136	-1.084	-1.397	-1.292
442	RPPos	PC(40:2)	6.60	-1.149	-1.166	-1.264	-1.236	-1.238	-1.173	-1.157	-1.506	-1.462
443	RPPos	PC(40:2)	6.42	1.012	1.119	-1.258	-1.143	-1.027	-1.219	-1.469	-1.379	-1.288
444	RPPos	PC(40:4)	5.46	1.095	1.106	1.028	1.053	1.140	1.025	1.016	1.198	1.024
445	RPPos	PC(40:4)	5.76	1.012	1.027	-1.138	1.016	1.041	-1.028	-1.064	1.009	-1.288
446	RPNeg	PC(40:4)>PC(18:0_22:4)	5.51	1.160	1.150	1.093	1.066	1.200	1.144	-1.026	1.229	1.065
447	RPNeg	PC(40:4)>PC(18:0_22:4)	5.81	1.092	1.029	-1.176	-1.016	1.077	-1.014	-1.079	-1.048	-1.256
448	RPPos	PC(40:5)	5.21	1.125	1.083	1.075	1.099	1.095	-1.108	-1.003	1.245	-1.186
449	RPPos	PC(40:5)	4.93	1.134	1.063	1.110	1.059	1.140	1.052	1.026	1.107	-1.001
450	RPPos	PC(40:5)	4.19	1.062	1.107	1.017	-1.059	-1.061	-1.383	1.296	1.365	1.044
451	RPPos	PC(40:5)	4.10	-1.026	1.112	-1.113	1.054	1.156	1.121	1.205	1.461	1.431
452	RPPos	PC(40:5)	4.59	-1.013	-1.104	-1.130	1.067	-1.097	-1.318	-1.061	-1.201	-1.432
453	RPNeg	PC(40:5)>PC(18:0_22:5)	4.98	1.083	1.145	1.065	1.031	1.087	-1.068	-1.022	1.207	1.056
454	RPNeg	PC(40:5)>PC(18:0_22:5)	5.25	1.067	1.086	1.010	1.078	1.099	1.091	-1.022	1.141	-1.160
455	RPNeg	PC(40:5)>PC(20:1_20:4)	4.65	1.101	-1.034	-1.214	1.207	-1.047	-1.511	1.232	1.009	-1.453
456	RPPos	PC(40:6)	4.30	1.223	-1.008	-1.235	1.137	1.313	-1.119	1.144	1.706	-1.004
457	RPPos	PC(40:6)	4.69	1.100	1.065	1.039	1.130	1.107	1.121	-1.018	1.197	1.031
458	RPPos	PC(40:6)	4.30	1.230	-1.002	-1.229	1.134	1.312	-1.119	1.146	1.725	-1.004
459	RPNeg	PC(40:6)>PC(18:0_22:6)	4.74	1.140	1.098	1.104	1.053	1.123	1.138	-1.050	1.192	1.082
460	RPNeg	PC(40:6)>PC(18:0_22:6)	4.35	-1.180	1.225	-1.274	-1.165	1.129	-1.025	-1.064	1.518	-1.276
461	RPPos	PC(40:7)	4.02	1.138	1.092	-1.005	1.104	-1.020	-1.001	1.037	1.138	-1.030
462	RPPos	PC(40:7)	3.86	1.189	1.112	1.016	1.134	-1.103	-1.353	1.011	1.377	-1.403
463	RPPos	PC(40:7)	3.74	-1.005	1.091	1.211	1.042	1.026	1.005	-1.134	1.014	-1.181
464	RPNeg	PC(40:7)>PC(18:1_22:6)	4.09	1.163	1.025	1.034	1.084	-1.012	-1.014	-1.023	1.102	-1.192
465	RPNeg	PC(40:7)>PC(18:2_22:5)	3.81	1.062	-1.052	1.005	1.061	1.012	1.071	-1.077	1.126	-1.076
466	RPPos	PC(40:8)	3.61	1.037	1.106	1.096	-1.018	1.076	1.090	-1.059	1.190	1.115

467	RPNeg	PC(40:8)>PC(20:4_20:4)	3.67	1.106	1.084	1.085	-1.025	1.067	1.069	-1.054	1.175	1.101
468	RPPos	PC(40:9)	3.29	-1.161	-1.046	1.191	1.105	-1.068	1.275	-1.042	-1.062	-1.413
469	RPNeg	PC(40:9)	3.36	1.052	-1.118	1.096	-1.043	1.002	1.094	-1.150	-1.130	-1.238
470	RPPos	PC(41:1)	6.95	-1.117	-1.122	-1.192	-1.039	-1.132	-1.009	-1.167	-1.331	-1.095
471	RPPos	PC(41:2)	6.77	-1.140	-1.020	-1.198	-1.236	-1.198	1.045	-1.167	-1.364	-1.253
472	RPPos	PC(41:3)	3.19	-1.062	-1.163	-1.280	-1.137	-1.482	-1.050	-1.323	-1.186	-1.541
473	RPPos	PC(41:6)	5.10	1.022	1.069	1.004	1.079	1.157	1.082	-1.014	1.226	1.119
474	RPPos	PC(41:6)	4.94	1.075	-1.019	1.121	-1.012	1.157	1.130	-1.001	1.091	-1.107
475	RPPos	PC(41:7)	3.57	-1.044	-1.101	-1.291	-1.023	-1.205	-1.257	-1.105	-1.338	-1.469
476	RPPos	PC(41:7)	4.34	1.153	1.108	-1.101	1.179	1.093	-1.133	1.195	1.425	-1.043
477	RPPos	PC(41:7)	4.45	1.070	-1.051	1.102	1.260	1.350	1.202	-1.076	1.203	1.076
478	RPPos	PC(42:1)	7.08	-1.130	-1.115	-1.122	-1.168	-1.225	1.011	-1.133	-1.292	-1.225
479	RPNeg	PC(42:1)>PC(18:1_24:0)	7.14	-1.118	-1.211	-1.179	-1.240	-1.032	-1.203	-1.141	-1.359	-1.199
480	RPPos	PC(42:10)	3.48	1.059	1.168	1.069	1.045	1.093	1.065	-1.028	1.290	1.167
481	RPNeg	PC(42:10)>PC(20:4_22:6)	3.54	1.157	1.186	-1.011	1.039	1.105	1.152	-1.017	1.235	1.037
482	RPPos	PC(42:2)	6.90	-1.021	-1.125	-1.214	-1.163	-1.236	1.009	-1.238	-1.504	-1.294
483	RPPos	PC(42:3)	6.56	-1.089	-1.136	-1.233	-1.202	-1.202	-1.068	-1.251	-1.651	-1.373
484	RPPos	PC(42:4)	6.54	-1.076	-1.047	-1.186	-1.126	-1.048	-1.131	-1.102	-1.101	-1.201
485	RPPos	PC(42:4)	6.13	1.091	1.031	-1.038	-1.037	1.117	1.063	1.066	1.203	1.332
486	RPPos	PC(42:5)	5.57	1.002	1.084	1.087	1.055	1.080	1.369	1.102	1.281	1.339
487	RPPos	PC(42:5)	5.71	-1.013	-1.009	-1.146	-1.026	-1.001	-1.046	-1.096	-1.062	-1.108
488	RPPos	PC(42:6)	5.18	1.155	1.134	1.022	1.141	1.169	1.007	-1.041	1.160	-1.348
489	RPPos	PC(42:6)	5.55	1.116	-1.010	1.006	1.024	-1.129	1.043	1.021	1.075	-1.196
490	RPPos	PC(42:6)	4.73	1.184	1.135	-1.125	-1.005	-1.024	-1.206	1.030	1.168	-1.317
491	RPPos	PC(42:6)	4.73	1.177	1.129	-1.120	-1.012	-1.031	-1.153	1.027	1.175	-1.279
492	RPPos	PC(42:7)	4.70	1.123	1.032	-1.027	1.079	1.120	1.052	-1.005	1.112	-1.175
493	RPPos	PC(42:7)	4.34	1.069	1.189	1.053	1.218	1.379	1.040	1.101	1.676	-1.007
494	RPPos	PC(42:7)	4.45	-1.078	1.139	1.113	-1.029	-1.039	-1.016	1.155	1.061	-1.008
495	RPNeg	PC(42:7)>PC(20:1_22:6)	4.75	1.186	1.069	-1.070	1.056	1.052	-1.017	1.083	1.152	-1.121
496	RPPos	PC(42:8)	4.03	-1.034	-1.245	-1.165	1.023	-1.044	1.063	1.081	-1.237	1.048
497	RPPos	PC(42:8)	4.13	1.290	1.208	1.166	1.034	1.013	1.150	1.048	1.070	1.090
498	RPPos	PC(42:8)	3.69	1.098	1.040	-1.107	1.143	1.065	-1.025	-1.047	1.141	-1.325
499	RPPos	PC(42:9)	3.69	1.233	1.210	-1.224	1.250	1.036	-1.124	1.019	1.092	-1.186
500	RPPos	PC(43:4)	6.72	-1.198	1.040	-1.144	-1.099	1.044	1.081	-1.106	-1.145	-1.031
501	RPPos	PC(44:12)	3.36	1.083	1.149	1.048	-1.057	-1.063	-1.133	1.017	1.233	1.030
502	RPPos	PC(44:2)	7.05	-1.206	-1.157	-1.218	-1.240	-1.183	-1.165	-1.281	-1.476	-1.205
503	RPPos	PC(44:2)	7.16	1.013	-1.190	-1.144	-1.159	-1.366	1.048	-1.226	-1.732	-1.602

504	RPPos	PC(44:4)	6.85	-1.072	-1.024	-1.167	-1.060	1.022	1.010	-1.093	-1.106	-1.115
505	RPPos	PC(44:4)	6.68	1.101	1.093	-1.107	-1.152	1.135	1.161	1.060	1.143	1.169
506	RPPos	PC(44:5)	6.48	-1.063	-1.096	-1.166	-1.153	-1.081	-1.055	-1.133	-1.231	-1.237
507	RPNeg	PC(44:5)>PC(20:4_24:1)	6.55	-1.036	-1.046	-1.190	1.046	-1.050	1.031	-1.187	-1.394	-1.177
508	RPPos	PC(44:6)	6.40	1.029	-1.095	-1.223	-1.052	-1.069	1.051	-1.062	-1.094	-1.098
509	RPPos	PC(46:5)	6.83	-1.131	-1.058	-1.223	-1.155	1.039	-1.042	-1.124	-1.327	-1.171
510	RPPos	PC(46:7)	6.35	-1.005	1.064	-1.107	-1.070	-1.012	1.044	-1.098	1.025	1.031
511	RPPos	PC(O-16:1) or PC(P-16:0)	2.13	-1.047	-1.164	-1.032	-1.033	-1.250	1.061	-1.077	-1.296	1.232
512	RPPos	PC(O-18:0)	2.46	1.281	-1.093	-1.468	1.235	-1.124	-1.534	1.659	1.678	1.451
513	RPNeg	PC(O-18:0)	2.53	1.240	-1.181	1.014	-1.057	1.101	1.007	1.074	-1.125	1.085
514	RPPos	PC(O-18:1) or PC(P-18:0)	2.44	1.322	-1.118	-1.270	1.309	-1.052	-1.187	1.625	1.371	1.859
515	RPPos	PC(O-18:1) or PC(P-18:0)	2.21	1.003	-1.128	1.062	1.043	-1.218	-1.030	-1.064	-1.184	1.062
516	RPNeg	PC(O-18:1) or PC(P-18:0)	2.50	1.161	-1.097	1.067	1.021	-1.134	1.042	1.057	-1.207	1.190
517	RPNeg	PC(O-18:1) or PC(P-18:0)	2.28	1.072	-1.147	1.059	1.047	-1.046	1.129	-1.054	-1.368	1.029
518	RPPos	PC(O-26:1)	3.52	1.015	-1.041	-1.043	1.016	-1.017	-1.049	-1.010	-1.017	-1.089
519	RPPos	PC(O-26:1)	3.39	1.135	1.045	-1.044	1.269	1.311	1.130	1.002	-1.091	-1.055
520	RPNeg	PC(O-26:1)	3.58	1.033	1.070	1.046	-1.001	1.051	-1.075	-1.049	-1.119	-1.097
521	RPPos	PC(O-30:0)	4.44	1.152	-1.001	-1.249	1.109	-1.121	-1.059	-1.112	-1.265	-1.249
522	RPPos	PC(O-31:0)	4.81	-1.040	-1.051	-1.152	1.105	1.027	-1.029	-1.213	-1.313	-1.243
523	RPPos	PC(O-32:0)	5.23	1.003	1.002	-1.123	-1.023	1.008	-1.140	-1.031	-1.060	-1.037
524	RPNeg	PC(O-32:0)>PC(O-16:0/16:0)	5.28	1.021	1.001	-1.092	1.010	1.015	-1.121	-1.014	-1.036	-1.013
525	RPPos	PC(O-32:1)	5.11	1.053	1.009	-1.086	-1.023	-1.001	-1.078	-1.086	-1.073	1.040
526	RPPos	PC(O-32:1)	4.49	1.219	1.116	-1.116	-1.017	-1.154	-1.171	-1.202	-1.230	-1.132
527	RPPos	PC(O-32:1)	4.77	1.050	1.068	-1.184	-1.147	-1.272	-1.424	-1.273	-1.194	-1.603
528	RPNeg	PC(O-32:1)>PC(O-16:1/16:0)	5.17	1.064	1.005	-1.093	-1.044	1.081	1.010	-1.023	1.011	1.144
529	RPPos	PC(O-32:2)	4.47	1.121	1.231	-1.212	1.052	-1.044	1.061	-1.094	-1.051	1.086
530	RPPos	PC(O-33:0)	5.68	1.077	-1.007	-1.072	1.217	-1.031	-1.218	-1.155	-1.279	-1.085
531	RPPos	PC(O-33:0)	5.51	1.124	1.049	-1.085	1.492	1.001	-1.121	1.027	-1.128	-1.240
532	RPPos	PC(O-33:1) or PC(P-33:0) or PE(O-36:1)	6.45	-1.134	-1.163	-1.189	-1.010	-1.217	-1.225	-1.121	-1.416	-1.661
533	RPPos	PC(O-34:0)	6.13	-1.069	-1.118	-1.198	-1.017	-1.089	-1.201	-1.007	-1.202	-1.097
534	RPPos	PC(O-34:1)	5.27	1.048	1.048	-1.144	-1.009	1.069	-1.133	-1.007	1.032	-1.141
535	RPNeg	PC(O-34:1)>PC(O-18:1/16:0)	5.33	1.104	1.065	-1.082	-1.007	1.076	-1.093	1.036	1.019	1.105
536	RPPos	PC(O-34:2)	4.70	-1.096	-1.074	-1.277	-1.060	-1.083	-1.083	-1.133	-1.147	-1.107
537	RPPos	PC(O-34:2)	5.19	1.052	1.033	-1.115	1.003	-1.001	-1.069	-1.011	-1.024	1.130
538	RPPos	PC(O-34:3) or PC(P-34:2)	4.04	1.021	-1.419	-1.325	1.014	-1.206	-1.059	-1.425	-1.232	-1.931
539	RPPos	PC(O-34:3) or PC(P-34:2)	4.58	1.044	1.038	-1.127	1.019	1.006	-1.068	-1.052	-1.073	-1.044

540	RPNeg	PC(O-34:3) or PC(P-34:2)	4.62	1.037	-1.007	-1.159	-1.079	-1.089	-1.111	-1.029	-1.060	-1.029
541	RPPos	PC(O-34:3) or PC(P-34:2) or PE(O-37:3)	5.24	-1.031	1.017	-1.116	1.026	1.074	-1.129	-1.116	-1.040	-1.003
542	RPPos	PC(O-34:4) or PC(P-34:3)	3.88	-1.041	-1.179	-1.240	-1.141	-1.470	-1.165	-1.183	-1.488	-1.700
543	RPPos	PC(O-34:8) or PC(P-34:3)	4.33	1.134	1.292	-1.011	1.070	1.369	1.256	1.098	1.457	1.493
544	RPPos	PC(O-34:8)	3.78	1.119	1.183	-1.071	-1.384	-1.390	-1.009	-1.153	-1.330	-2.284
545	RPPos	PC(O-36:0)	6.70	-1.104	-1.072	-1.345	-1.083	-1.039	-1.136	1.072	-1.036	-1.254
546	RPPos	PC(O-36:1)	6.20	1.038	-1.024	-1.134	-1.063	-1.059	-1.197	-1.155	-1.058	-1.135
547	RPNeg	PC(O-36:1)>PC(O-18:0/18:1)	5.60	1.008	1.136	-1.122	-1.158	1.015	1.010	-1.031	1.221	1.036
548	RPNeg	PC(O-36:1)>PC(O-18:0/18:1)	5.41	-1.082	-1.040	-1.171	-1.083	1.034	-1.105	1.061	1.085	-1.011
549	RPNeg	PC(O-36:1)>PC(O-18:1/18:0) and PC(O-16:0/20:1)	6.25	-1.123	-1.145	-1.299	1.146	-1.087	-1.143	-1.020	-1.311	-1.088
550	RPPos	PC(O-36:2)	2.95	-1.753	1.204	-2.266	-2.347	-1.594	-3.331	1.290	1.519	-1.689
551	RPPos	PC(O-36:2)	5.56	-1.048	-1.038	-1.172	-1.072	-1.145	-1.140	-1.008	-1.139	-1.064
552	RPPos	PC(O-36:2)	5.35	1.356	-1.133	-1.286	1.063	1.080	-1.001	1.075	1.035	1.062
553	RPPos	PC(O-36:2)	6.07	1.032	-1.016	-1.264	-1.039	1.001	-1.011	-1.022	1.058	1.138
554	RPPos	PC(O-36:2)	5.57	-1.093	-1.001	-1.181	-1.044	-1.155	-1.186	-1.066	-1.193	-1.295
555	RPPos	PC(O-36:3)	4.74	-1.005	1.000	-1.155	1.051	-1.039	-1.111	-1.032	-1.027	-1.122
556	RPPos	PC(O-36:3)	4.73	-1.013	-1.001	-1.176	1.025	-1.075	-1.170	-1.080	-1.020	-1.208
557	RPPos	PC(O-36:4)	4.58	1.007	1.024	-1.040	1.034	1.121	-1.008	-1.098	-1.015	1.012
558	RPPos	PC(O-36:4)	4.44	1.132	1.036	1.128	1.130	-1.033	1.045	-1.082	-1.234	-1.178
559	RPNeg	PC(O-36:4)>PC(O-16:0/20:4)	4.63	1.047	1.026	-1.072	-1.009	1.045	-1.039	-1.029	1.102	1.068
560	RPPos	PC(O-36:5)	4.45	1.218	1.096	1.031	1.089	1.131	1.181	-1.040	1.113	1.378
561	RPPos	PC(O-36:5)	3.94	1.029	-1.191	-1.163	-1.127	-1.246	-1.303	-1.135	-1.254	-1.393
562	RPNeg	PC(O-36:5)>PC(O-16:1/20:4)	4.51	1.079	1.072	-1.030	1.030	1.197	1.102	-1.027	1.104	1.436
563	RPPos	PC(O-37:5)	4.83	1.076	1.014	1.129	1.056	1.072	1.143	1.031	-1.026	1.197
564	RPPos	PC(O-38:0)	6.98	1.111	1.039	-1.150	-1.207	-1.220	-1.294	1.014	-1.144	-1.408
565	RPPos	PC(O-38:0)	6.76	-1.010	-1.088	-1.119	-1.132	1.011	-1.246	-1.049	-1.062	-1.001
566	RPPos	PC(O-38:1) or PC(P-38:0)	6.73	-1.037	-1.032	-1.223	-1.045	-1.101	-1.064	-1.001	-1.039	1.000
567	RPNeg	PC(O-38:1) or PC(P-38:0)	6.98	-1.035	-1.094	1.081	1.103	1.101	1.229	-1.025	-1.043	1.063
568	RPPos	PC(O-38:2)	6.42	1.016	-1.341	-1.155	-1.054	-1.082	-1.229	1.064	-1.296	-1.439
569	RPPos	PC(O-38:2)	6.17	-1.017	1.045	-1.106	-1.014	1.106	-1.028	1.050	1.148	1.065
570	RPPos	PC(O-38:3)	5.77	1.327	1.247	-1.004	1.022	1.076	1.077	-1.145	1.130	1.054
571	RPPos	PC(O-38:3)	6.31	-1.072	-1.038	-1.144	-1.031	-1.104	-1.200	-1.089	-1.225	-1.210
572	RPPos	PC(O-38:4)	5.43	1.033	1.058	-1.225	-1.035	1.045	-1.079	-1.032	-1.026	-1.207
573	RPPos	PC(O-38:4)	5.13	1.030	1.159	-1.057	-1.016	1.311	1.111	-1.009	1.107	1.278
574	RPPos	PC(O-38:4)	5.19	1.046	-1.026	-1.037	1.171	-1.038	-1.043	-1.113	-1.215	-1.177

575	RPNeg	PC(O-38:4)>PC(O-16:0/22:4)	5.19	1.031	1.116	-1.015	-1.046	1.209	1.098	-1.021	1.056	1.221
576	RPNeg	PC(O-38:4)>PC(O-18:0/20:4)	5.47	1.107	1.069	-1.170	1.008	1.053	-1.126	-1.056	1.034	-1.048
577	RPPos	PC(O-38:6)	4.06	1.061	-1.023	-1.155	1.043	1.062	-1.099	-1.034	1.122	1.015
578	RPPos	PC(O-38:6)	4.74	1.013	1.184	-1.084	1.129	1.113	1.117	1.147	1.117	-1.005
579	RPPos	PC(O-38:6)	4.74	1.013	1.184	-1.084	1.129	1.113	1.117	1.147	1.117	-1.005
580	RPNeg	PC(O-38:6)>PC(O-16:0/22:6)	4.44	1.062	1.054	1.026	-1.096	1.124	1.123	-1.093	1.180	1.090
581	RPNeg	PC(O-38:6)>PC(O-18:2/20:4)	4.12	-1.079	-1.078	-1.168	-1.109	1.088	-1.099	-1.006	1.041	-1.034
582	RPNeg	PC(O-38:6)>PC(O-18:2/20:4)	4.55	-1.011	1.042	1.010	-1.019	1.255	1.099	-1.088	1.105	1.222
583	RPPos	PC(O-38:7)	4.27	1.129	-1.025	1.019	1.057	1.342	1.123	-1.022	1.368	1.355
584	RPPos	PC(O-38:7)	3.74	-1.032	-1.078	-1.157	-1.067	-1.061	-1.042	-1.077	-1.080	-1.185
585	RPPos	PC(O-38:7)	4.27	1.127	1.089	1.025	1.024	1.271	1.127	1.047	1.406	1.510
586	RPNeg	PC(O-38:7)>PC(O-16:2/22:5)	5.07	-1.115	-1.310	-1.150	-1.017	-1.012	1.052	-1.029	-1.032	1.038
587	RPPos	PC(O-40:1) or PC(P-40:0)	7.00	-1.139	-1.110	-1.388	-1.086	-1.252	-1.216	-1.040	-1.081	-1.560
588	RPPos	PC(O-40:4)	6.33	1.054	1.003	-1.087	-1.054	-1.003	-1.079	-1.045	1.010	1.184
589	RPPos	PC(O-40:4)	6.02	1.015	1.035	-1.092	-1.031	1.081	-1.026	1.003	1.044	1.077
590	RPPos	PC(O-40:4)	5.81	1.030	1.005	-1.144	1.023	1.094	-1.020	-1.034	1.014	1.004
591	RPNeg	PC(O-40:4)>PC(O-20:0/20:4)	6.36	1.084	1.038	-1.079	-1.003	1.094	1.033	-1.045	-1.024	1.152
592	RPNeg	PC(O-40:4)>PC(O-20:0/20:4)	6.07	1.100	1.026	-1.169	-1.015	1.114	1.013	1.018	1.149	1.272
593	RPPos	PC(O-40:5) or PC(P-40:4)	5.45	1.319	1.175	1.080	-1.118	1.229	-1.050	-1.018	1.240	1.162
594	RPPos	PC(O-40:5) or PC(P-40:4)	5.18	1.169	1.066	-1.085	-1.048	1.117	1.145	-1.032	1.096	1.158
595	RPNeg	PC(O-40:5) or PC(P-40:4)	5.22	1.055	1.020	-1.079	-1.001	1.239	1.141	1.020	1.096	1.219
596	RPNeg	PC(O-40:5) or PC(P-40:4)	5.50	1.047	1.078	-1.122	-1.049	1.205	1.007	-1.043	1.033	1.104
597	RPPos	PC(O-40:6)	5.20	1.066	1.083	-1.166	-1.005	1.111	1.081	1.003	1.182	1.101
598	RPPos	PC(O-40:6)	4.65	-1.122	-1.081	-1.011	1.053	1.214	1.186	1.014	1.029	1.296
599	RPPos	PC(O-40:7)	4.44	1.150	1.137	-1.024	1.149	1.190	1.073	1.018	1.202	1.322
600	RPPos	PC(O-40:7)	5.05	1.066	1.058	1.025	1.148	1.249	1.267	1.005	1.294	1.337
601	RPPos	PC(O-40:7)	4.32	1.204	1.105	-1.000	1.207	1.468	1.172	1.138	1.607	1.298
602	RPPos	PC(O-40:7)	4.44	1.138	1.127	-1.028	1.157	1.179	1.077	1.011	1.189	1.312
603	RPNeg	PC(O-40:9)>PC(O-20:5/20:4)	4.94	1.012	1.139	-1.010	1.023	1.062	1.051	1.018	1.231	-1.030
604	RPPos	PC(O-42:3)	6.26	-1.223	-1.082	-1.126	-1.228	-1.137	-1.232	-1.132	-1.282	-1.313
605	RPPos	PC(O-42:6)	5.53	-1.033	1.124	-1.193	-1.068	1.172	-1.276	1.120	1.205	-1.085
606	RPPos	PC(O-42:6)	6.09	1.050	1.084	-1.031	-1.034	1.134	1.111	1.020	1.122	1.206
607	RPNeg	PC(O-38:5)>PC(O-18:1/20:4)	4.67	1.068	1.011	-1.071	-1.025	1.107	-1.081	-1.006	1.089	1.095
608	RPNeg	PC(O-38:5)>PC(O-18:1/20:4)	5.32	1.090	1.100	-1.055	-1.114	1.159	1.133	-1.019	1.066	1.313
609	RPPos	PE(34:1)	5.04	-1.161	-1.415	-1.149	-1.348	-2.217	-1.184	-1.530	-1.839	-1.952
610	RPNeg	PE(34:1)>PE(16:0_18:1)	5.09	-1.087	-1.583	-1.180	-1.262	-2.074	-1.096	-1.462	-2.140	-1.628
611	RPPos	PE(34:2)	4.45	-1.275	-1.122	-1.109	-1.227	-1.772	1.014	-2.078	-1.974	-1.373

612	RPNeg	PE(34:2)>PE(16:0_18:2)	4.51	-1.247	-1.115	-1.260	-1.419	-1.619	1.092	-2.141	-1.856	-1.401
613	RPPos	PE(36:1)	5.91	-1.505	-1.714	-1.414	-1.601	-2.099	-1.388	-1.967	-2.549	-2.041
614	RPNeg	PE(36:1)>PE(18:0_18:1)	5.96	-1.562	-1.729	-1.390	-1.608	-2.044	-1.293	-2.116	-2.410	-2.043
615	RPNeg	PE(36:2)>PE(18:0_18:2)	5.31	-1.507	-1.583	-1.297	-1.585	-1.664	-1.266	-2.308	-2.437	-1.963
616	RPNeg	PE(36:2)>PE(18:0_18:2)	4.29	-1.031	-1.041	-1.028	-1.089	-1.067	1.086	-1.134	-1.343	-1.139
617	RPPos	PE(36:2)>PE(18:2_18:0)	5.26	-1.438	-1.546	-1.320	-1.497	-1.645	-1.302	-2.196	-2.159	-2.219
618	RPPos	PE(36:2)>PE(18:2_18:0)	5.10	-1.243	-1.465	-1.417	-1.426	-1.962	-1.496	-1.738	-2.174	-1.763
619	RPPos	PE(36:2)>PE(18:2_18:0)	4.36	-1.305	1.176	-1.191	-1.318	-1.275	1.607	-2.094	-2.089	-1.520
620	RPPos	PE(36:3)	4.51	-1.495	-1.310	-1.381	-1.770	-1.941	-1.367	-2.412	-3.356	-2.179
621	RPNeg	PE(36:3)>PE(18:1_18:2)	4.54	-1.416	-1.299	-1.645	-1.655	-2.198	-1.346	-2.140	-3.675	-2.129
622	RPNeg	PE(36:4)>(16:0_20:4)	4.40	-1.082	-1.155	-1.090	-1.148	-1.589	-1.142	-1.339	-1.549	-1.362
623	RPPos	PE(36:4)>PE(16:0_20:4)	4.34	-1.001	-1.111	-1.146	-1.111	-1.520	-1.171	-1.300	-1.419	-1.305
624	RPPos	PE(36:5)	4.45	-1.704	-1.284	1.116	-1.106	-1.573	-1.014	-2.881	-1.897	1.107
625	RPPos	PE(36:5)	3.92	-1.311	-1.715	-1.289	-1.343	-1.937	-1.192	-1.926	-2.289	-1.831
626	RPNeg	PE(36:5)>PE(16:0_20:5)	3.98	-1.396	-1.291	-1.357	-1.581	-2.427	-1.044	-2.088	-2.491	-2.084
627	RPPos	PE(38:3)>PE(18:0_20:3)	5.47	-1.123	-1.364	-1.300	-1.326	-1.830	-1.472	-1.630	-2.051	-2.319
628	RPNeg	PE(38:4)>PE(18:0_20:4)	5.19	-1.166	-1.064	-1.108	-1.202	-1.487	-1.138	-1.348	-1.344	-1.327
629	RPPos	PE(38:4)>PE(18:0_20:4)	5.13	-1.135	-1.110	-1.143	-1.236	-1.702	-1.118	-1.353	-1.353	-1.384
630	RPNeg	PE(38:4)>PE(18:0_20:4)	5.19	-1.163	-1.076	-1.168	-1.236	-1.560	-1.122	-1.362	-1.363	-1.242
631	RPPos	PE(38:5)	4.62	-1.447	-1.320	-1.424	-1.213	-1.523	-1.487	-2.168	-2.415	-2.316
632	RPPos	PE(38:5)	5.27	-1.550	-1.842	-1.284	-1.686	-1.647	-1.137	-2.531	-2.352	-2.050
633	RPPos	PE(38:5) or PC(35:5)	3.47	-1.178	-1.155	-1.288	-1.161	-1.472	-1.249	-1.175	-1.396	-1.634
634	RPNeg	PE(38:5)>PE(18:0_20:5)	4.68	-1.302	-1.239	-1.188	-1.391	-1.875	-1.180	-1.781	-2.042	-2.112
635	RPPos	PE(38:5)>PE(18:1_20:4)	4.41	2.038	1.202	1.681	1.159	-1.253	1.415	-1.026	-1.534	-1.288
636	RPPos	PE(38:6)	3.60	-1.055	-1.094	-1.291	-1.070	-1.320	-1.070	-1.092	-1.491	-1.476
637	RPPos	PE(38:6)	3.88	-1.288	-1.208	-1.377	1.023	-1.259	1.006	-1.507	-1.896	-1.497
638	RPPos	PE(38:6)	3.88	-1.294	-1.167	-1.305	-1.348	-1.256	-1.044	-1.358	-1.853	-1.533
639	RPPos	PE(38:6)	4.17	1.131	1.153	1.124	1.064	-1.587	-1.027	-1.143	-1.267	-1.297
640	RPNeg	PE(38:6)>PE(18:2_20:4)	3.95	-1.092	-1.060	-1.201	-1.031	-1.408	-1.165	-1.288	-1.918	-1.321
641	RPPos	PE(40:4)>PE(18:0_22:4)	5.72	-1.303	-1.176	-1.437	-1.008	-1.315	-1.337	-1.368	-1.478	-1.578
642	RPPos	PE(40:5)	5.17	1.142	1.083	-1.010	-1.217	-1.489	-1.313	-1.368	-1.521	-1.401
643	RPPos	PE(40:5)	5.46	-1.316	-1.387	-1.317	-1.487	-1.918	-1.164	-1.552	-1.648	-1.974
644	RPPos	PE(40:6)	4.94	-1.172	-1.087	-1.175	-1.368	-1.717	-1.083	-1.469	-1.755	-1.519
645	RPNeg	PE(40:6)>PE(18:0_22:6)	4.99	-1.143	-1.226	-1.118	-1.214	-1.474	-1.074	-1.346	-1.546	-1.503
646	RPNeg	PE(40:7)>PE(18:1_22:6) and PE(18:0_22:7)	4.28	-1.204	-1.417	-1.503	-1.321	-2.079	-1.383	-1.640	-2.028	-1.913
647	RPPos	PE(42:10)	3.63	1.038	-1.182	-1.237	1.329	-1.319	-1.061	1.103	-1.166	1.037

648	RPPos	PE(44:4)	5.72	1.060	1.045	-1.011	1.214	1.119	1.200	1.083	1.210	-1.197
649	RPPos	PE(44:4)	5.91	1.037	-1.015	-1.008	-1.085	1.056	-1.077	1.138	1.314	1.277
650	RPPos	PE(44:4)	6.21	-1.112	-1.050	-1.122	1.019	-1.079	1.005	-1.163	-1.194	-1.122
651	RPPos	PE(O-34:1)	5.59	1.025	-1.206	-1.256	-1.268	-1.314	-1.339	-1.144	-1.605	-2.098
652	RPPos	PE(O-34:1)>PE(O-18:1/16:0)	6.30	1.074	1.048	-1.134	-1.132	-1.273	-1.042	-1.033	-1.656	-1.398
653	RPPos	PE(O-34:2)>PE(O-16:1/18:1) and PE(O-18:2/16:0)	5.47	-1.046	1.005	-1.332	1.124	-1.379	-1.330	-1.005	-1.324	-1.635
654	RPNeg	PE(O-34:2)>PE(O-16:2/18:0) and PE(O-18:2/16:0)	5.52	-1.002	-1.006	-1.306	1.042	-1.320	-1.286	-1.030	-1.429	-1.371
655	RPNeg	PE(O-34:5)>PE(O-17:1/20:4)	5.17	-1.031	-1.100	-1.216	1.035	-1.197	-1.223	-1.176	-1.186	-1.108
656	RPPos	PE(O-36:2)	5.86	-1.040	-1.045	-1.154	1.064	-1.169	-1.125	-1.068	-1.250	-1.313
657	RPPos	PE(O-36:3)>PE(O-18:1/18:2)	5.71	-1.129	1.028	-1.135	1.042	-1.123	-1.194	-1.098	-1.223	-1.104
658	RPPos	PE(O-36:3)>PE(O-18:2/18:1)	5.54	-1.036	-1.181	-1.120	-1.200	-1.199	-1.231	1.090	-1.158	-1.082
659	RPPos	PE(O-36:4)	4.83	-1.187	-1.424	-1.082	-1.067	-1.562	-1.126	-1.232	-1.817	-1.904
660	RPNeg	PE(O-36:4)>PE(O-16:0/20:4)	4.89	-1.457	-1.308	-1.186	1.039	-1.564	-1.551	-1.242	-1.593	-1.492
661	RPPos	PE(O-36:4)>PE(O-16:1/20:3)	5.13	-1.116	-1.008	-1.131	-1.038	-1.298	-1.279	-1.038	-1.242	-1.377
662	RPPos	PE(O-36:5)>PE(O-16:1/20:4)	4.71	-1.131	-1.177	-1.161	1.120	-1.164	-1.209	1.055	-1.310	-1.259
663	RPNeg	PE(O-36:5)>PE(O-16:1/20:4)	4.76	-1.035	-1.116	-1.217	1.060	-1.223	-1.183	-1.045	-1.340	-1.316
664	RPPos	PE(O-37:5)>PE(O-17:1/20:4)	5.11	-1.164	-1.014	-1.238	1.074	-1.209	-1.185	-1.059	-1.155	-1.290
665	RPPos	PE(O-38:2)	6.51	-1.017	1.017	-1.096	-1.106	-1.065	-1.018	-1.101	-1.249	1.084
666	RPNeg	PE(O-38:2)>PE(O-20:0/18:2)	6.68	1.047	1.029	-1.166	1.092	-1.016	-1.088	-1.050	-1.009	-1.037
667	RPPos	PE(O-38:4)	5.42	-1.212	-1.148	-1.145	1.003	-1.111	-1.236	-1.142	-1.426	-1.590
668	RPNeg	PE(O-38:4)>PE(O-16:0/22:4)	5.47	-1.127	-1.153	-1.182	-1.123	-1.297	-1.341	-1.071	-1.441	-1.476
669	RPNeg	PE(O-38:4)>PE(O-18:0/20:4) and PE(O-18:2/20:2)	5.77	-1.129	-1.165	-1.159	-1.093	-1.334	-1.387	-1.115	-1.507	-1.170
670	RPPos	PE(O-38:5)>PE(O-16:1/22:4)	5.27	-1.055	1.015	-1.194	1.143	-1.101	-1.161	-1.032	-1.153	-1.356
671	RPNeg	PE(O-38:5)>PE(O-18:1/20:4)	5.61	-1.039	-1.068	-1.341	1.026	-1.228	-1.142	-1.048	-1.317	-1.231
672	RPPos	PE(O-38:5)>PE(O-18:1/20:4)	5.56	-1.092	-1.090	-1.260	1.023	-1.230	-1.158	-1.015	-1.261	-1.296
673	RPNeg	PE(O-38:5)>PE(O-18:1/20:4)	5.61	-1.000	-1.031	-1.226	1.085	-1.186	-1.108	1.016	-1.233	-1.143
674	RPPos	PE(O-38:6)>PE(O-18:2/20:4) and PE(O-16:1/22:5)	4.75	-1.065	1.085	-1.119	1.094	-1.171	-1.187	-1.086	-1.244	-1.106
675	RPPos	PE(O-38:6)>PE(O-18:2/20:4) and PE(O-16:1/22:5)	4.62	1.243	1.030	-1.125	-1.037	-1.057	-1.043	-1.236	-1.542	-1.602
676	RPNeg	PE(O-38:6)>PE(O-18:2/20:4) and PE(O-16:1/22:5)	5.07	1.017	-1.003	-1.079	1.052	-1.073	-1.256	-1.004	-1.273	-1.079
677	RPNeg	PE(O-38:6)>PE(O-18:2/20:4) and PE(O-16:1/22:5)	4.81	1.032	-1.137	-1.196	1.061	-1.221	-1.258	-1.039	-1.256	-1.258

678	RPNeg	PE(O-38:6)>PE(O-18:2/20:4) and PE(O-16:1/22:5)	4.81	1.040	-1.068	-1.207	1.084	-1.197	-1.199	-1.007	-1.233	-1.282
679	RPPos	PE(O-38:7)>PE(O-16:1/22:6)	4.51	1.096	1.068	-1.013	-1.020	-1.012	-1.010	-1.046	-1.056	1.071
680	RPNeg	PE(O-38:7)>PE(O-16:1/22:6) and PE(O-20:7/18:0)	4.54	1.152	1.125	-1.192	-1.025	-1.019	-1.029	-1.074	-1.103	1.094
681	RPNeg	PE(O-38:7)>PE(O-20:7/18:0) and PE(O-16:1/22:6)	4.55	1.034	1.027	-1.038	1.031	1.022	1.032	-1.125	-1.113	1.066
682	RPPos	PE(O-39:3)	5.20	1.049	-1.025	-1.084	-1.029	1.199	-1.181	1.062	1.014	1.004
683	RPPos	PE(O-39:7)	3.43	-1.318	-1.385	-1.543	-1.262	-1.776	-1.260	-1.218	-1.857	-1.735
684	RPPos	PE(O-40:0)	4.03	-1.006	-1.027	-1.135	1.521	-1.020	-1.124	-1.147	-1.015	1.169
685	RPPos	PE(O-40:4)	6.52	1.027	-1.074	-1.201	-1.054	-1.134	-1.150	-1.060	-1.147	-1.109
686	RPPos	PE(O-40:4)	6.30	-1.074	1.027	-1.127	-1.056	-1.089	-1.096	1.008	-1.158	-1.018
687	RPNeg	PE(O-40:4)>PE(O-18:0/22:4)	6.35	-1.081	-1.099	-1.155	-1.097	-1.229	-1.236	-1.102	-1.300	-1.264
688	RPPos	PE(O-40:5)>PE(O-18:1/22:4)	6.16	-1.049	-1.086	-1.187	-1.048	-1.220	-1.130	-1.020	-1.237	-1.146
689	RPPos	PE(O-40:5)>PE(O-18:1/22:4)	5.75	1.012	-1.001	-1.210	-1.020	-1.156	-1.209	-1.028	-1.234	-1.194
690	RPPos	PE(O-40:5)>PE(O-18:1/22:4)	6.41	1.032	-1.020	-1.140	-1.043	-1.022	1.003	-1.000	-1.201	-1.065
691	RPNeg	PE(O-40:5)>PE(O-18:1/22:4)	6.21	-1.028	-1.041	-1.256	1.183	-1.135	-1.109	-1.044	-1.282	-1.182
692	RPNeg	PE(O-40:6)>PE(O-18:1/22:5)	5.93	1.164	-1.062	-1.252	-1.078	-1.278	-1.194	-1.022	-1.295	-1.077
693	RPNeg	PE(O-40:6)>PE(O-18:1/22:5)	5.65	-1.010	-1.015	1.002	-1.044	-1.420	-1.196	-1.083	-1.470	1.001
694	RPNeg	PE(O-40:6)>PE(O-18:1/22:5)	5.53	-1.050	-1.068	-1.380	1.009	-1.086	-1.074	1.073	-1.218	-1.237
695	RPPos	PE(O-40:6)>PE(O-18:1/22:5)	5.89	1.042	-1.001	-1.316	-1.028	-1.232	-1.197	-1.041	-1.222	-1.104
696	RPPos	PE(O-40:6)>PE(O-18:1/22:5)	5.60	-1.016	-1.178	-1.273	1.112	-1.470	-1.113	1.095	-1.199	-1.156
697	RPNeg	PE(O-40:6)>PE(O-18:2/22:4)	5.39	-1.062	1.060	-1.171	-1.034	-1.083	-1.337	1.045	-1.093	-1.033
698	RPPos	PE(O-40:6)>PE(O-18:2/22:4)	5.34	-1.125	-1.211	-1.432	-1.048	-1.206	-1.122	1.012	-1.140	-1.220
699	RPPos	PE(O-40:7)>PE(O-18:1/22:6)	5.32	1.018	1.052	-1.140	-1.029	-1.016	-1.065	1.004	-1.020	1.080
700	RPNeg	PE(O-40:7)>PE(O-18:1/22:6) and PE(O-22:6/18:1)	5.38	1.097	-1.002	-1.035	1.024	1.077	1.042	1.058	-1.054	1.270
701	RPNeg	PE(O-40:7)>PE(O-18:1/22:6) and PE(O-22:7/18:0)	5.38	1.078	1.047	-1.124	-1.001	-1.003	-1.054	-1.042	-1.036	1.217
702	RPNeg	PE(O-40:7)>PE(O-18:2/22:5)	4.86	-1.042	1.011	-1.294	-1.055	1.046	-1.043	1.020	-1.242	-1.158
703	RPPos	PE(O-40:7)>PE(O-18:2/22:5)	4.80	-1.157	-1.119	-1.306	1.167	-1.107	-1.144	1.185	-1.003	1.138
704	RPPos	PE(O-40:8)>PE(O-18:2/22:6)	4.55	-1.014	1.093	-1.102	-1.038	1.082	-1.017	1.017	1.029	1.113
705	RPNeg	PE(O-40:8)>PE(O-22:8/18:0) and PE(O-18:2/22:6)	4.61	1.017	1.066	-1.072	-1.021	-1.008	-1.028	-1.034	1.022	1.069
706	RPNeg	PE(O-42:5)>PE(O-20:1/22:4)	6.78	-1.038	1.050	-1.059	1.011	1.048	1.092	-1.005	-1.151	1.087
707	RPNeg	PE(O-42:7)>PE(O-20:1/22:6)	6.27	1.012	1.037	-1.107	1.012	1.036	1.027	-1.049	-1.067	1.126
708	RPNeg	PE(P-40:3)>PE(P-18:0/22:3)	6.58	-1.034	-1.003	-1.149	-1.027	-1.035	-1.006	-1.092	-1.086	-1.114
709	RPNeg	PE(P-40:4)>PE(P-20:0/20:4)	6.47	1.027	1.006	-1.103	-1.036	-1.097	-1.040	1.018	-1.111	1.046

710	RPNeg	PE(P-40:4)>PE(P-20:0/20:4)	6.47	-1.002	-1.050	-1.101	-1.005	-1.091	-1.036	-1.028	-1.176	1.064
711	RPPos	PG(40:8)	2.72	1.359	-1.194	-1.592	-1.254	-1.262	-2.492	1.803	1.771	-1.169
712	RPPos	PG(43:1)	4.43	1.313	1.138	1.212	-1.016	-1.116	-1.145	1.015	-1.005	-1.270
713	RPPos	phenylalanine	0.79	-1.206	-1.312	-1.429	-1.409	-1.736	-1.408	-2.116	-1.799	-1.944
714	RPPos	PI(34:1)	4.23	-1.055	-1.040	1.074	1.024	-1.244	1.077	1.000	-1.283	-1.171
715	RPNeg	PI(34:1)	4.29	1.159	1.025	-1.016	-1.022	-1.197	-1.071	-1.028	-1.147	-1.168
716	RPPos	PI(34:1)	4.22	-1.040	-1.102	1.084	1.044	-1.249	1.130	1.045	-1.293	-1.157
717	RPPos	PI(34:2)	3.75	-1.030	-1.015	1.048	1.028	-1.034	1.148	-1.124	-1.306	1.096
718	RPNeg	PI(34:2)>PI(16:0_18:2)	3.82	1.055	-1.078	1.112	-1.062	-1.125	1.136	-1.093	-1.142	1.076
719	RPPos	PI(36:1)	4.97	-1.250	-1.539	-1.197	1.103	-1.279	-1.079	-1.082	-2.097	-1.139
720	RPPos	PI(36:1)	4.97	-1.251	-1.523	-1.208	1.120	-1.312	-1.083	-1.089	-2.140	-1.160
721	RPNeg	PI(36:1)>PI(18:0_18:1)	5.04	-1.069	-1.306	-1.097	-1.076	-1.346	1.039	-1.072	-1.836	-1.094
722	RPPos	PI(36:2)	4.46	1.187	1.211	1.000	1.148	1.060	1.596	1.075	1.221	1.854
723	RPPos	PI(36:2)	4.41	1.104	-1.045	1.067	1.056	1.045	1.218	-1.038	-1.175	1.151
724	RPPos	PI(36:2)	2.65	1.788	1.260	-1.131	-1.070	-1.086	-1.731	1.197	1.270	-1.114
725	RPNeg	PI(36:2)>PI(18:0_18:2)	4.50	-1.043	-1.099	1.002	-1.018	-1.101	1.468	-1.132	-1.202	1.256
726	RPNeg	PI(36:2)>PI(18:1_18:1)	4.35	-1.208	-1.278	-1.193	1.010	-1.361	-1.049	-1.148	-1.838	-1.429
727	RPPos	PI(36:3)	3.81	-1.116	-1.183	-1.110	-1.082	-1.336	1.005	-1.192	-1.390	-1.236
728	RPNeg	PI(36:3)>PI(18:1_18:2)	3.88	-1.037	-1.220	-1.063	-1.009	-1.228	-1.014	-1.219	-1.748	-1.091
729	RPPos	PI(36:4)>PI(16:0_20:4)	3.68	1.087	1.100	1.012	1.057	1.106	1.022	-1.047	-1.008	-1.016
730	RPNeg	PI(36:4)>PI(16:0_20:4)	3.75	1.051	1.056	-1.011	1.007	1.120	-1.024	-1.037	-1.011	-1.084
731	RPPos	PI(38:3)>PI(18:0_20:3)	4.84	1.156	1.293	-1.064	1.220	1.191	-1.089	1.106	1.088	-1.267
732	RPPos	PI(38:3)>PI(18:0_20:3)	4.60	1.086	1.094	-1.180	1.153	1.303	-1.052	1.053	1.136	-1.213
733	RPNeg	PI(38:3)>PI(18:0_20:3)	4.67	1.224	1.202	1.029	1.078	1.264	-1.122	1.092	1.220	-1.111
734	RPPos	PI(38:3)>PI(18:0_20:3)	4.84	1.183	1.282	-1.094	1.255	1.197	-1.084	1.141	1.092	-1.277
735	RPNeg	PI(38:4)>PI(18:0_20:4)	4.38	1.109	1.166	1.022	1.084	1.249	1.141	-1.004	1.233	1.070
736	RPPos	PI(38:4)>PI(18:0_20:4)	4.31	1.130	1.213	-1.032	1.125	1.235	1.072	1.067	1.290	1.147
737	RPNeg	PI(38:4)>PI(18:0_20:4)	4.40	1.116	1.153	1.061	1.123	1.250	1.125	1.074	1.381	1.278
738	RPNeg	PI(38:5)>PI(18:1_20:4)	3.80	1.112	1.015	-1.032	1.103	-1.064	-1.037	-1.144	-1.086	-1.196
739	RPPos	PI(38:5)>PI(18:1_20:4)	3.29	-1.008	1.166	-1.055	-1.091	-1.072	-1.154	-1.078	1.037	1.180
740	RPPos	PI(38:5)>PI(18:1_20:4)	3.73	-1.055	1.067	-1.098	1.108	-1.066	-1.050	-1.076	-1.062	-1.253
741	RPPos	PI(38:5)>PI(18:1_20:4)	3.92	1.047	-1.055	-1.270	1.094	1.058	-1.016	-1.066	-1.157	-1.643
742	RPNeg	PI(40:4)>PI(18:0_22:4)	4.89	1.163	1.096	-1.049	1.063	1.207	-1.060	1.077	1.129	1.032
743	RPNeg	PI(40:5)>PI(18:0_22:5)	4.42	1.125	1.174	-1.042	1.221	1.317	-1.032	1.011	1.289	-1.029
744	RPNeg	PI(40:5)>PI(18:0_22:5)	4.67	1.106	1.111	-1.064	1.132	1.117	1.026	-1.046	1.011	-1.228
745	RPPos	PI(40:5)>PI(18:0_22:5)	4.35	1.245	1.172	-1.078	1.133	1.242	1.013	-1.030	1.208	-1.025
746	RPPos	PI(40:5)>PI(18:0_22:5)	4.60	1.062	1.056	-1.040	1.156	1.260	-1.037	1.005	-1.017	-1.246

747	RPPos	PI(40:5)>PI(18:0_22:5)	4.17	1.063	1.261	1.113	-1.016	1.276	1.059	1.000	1.385	1.087
748	RPPos	PI(40:5)>PI(18:0_22:5)	4.35	1.252	1.177	-1.062	1.114	1.238	1.017	-1.008	1.246	-1.040
749	RPPos	PI(40:5)>PI(18:0_22:5)	4.60	1.066	1.071	-1.063	1.155	1.246	-1.049	1.038	-1.022	-1.231
750	RPNeg	PI(40:6)>PI(18:0_22:6)	4.23	1.165	1.062	-1.008	1.118	1.242	1.154	-1.018	1.104	1.030
751	RPPos	PI(40:6)>PI(18:0_22:6)	4.17	1.173	1.185	1.037	1.049	1.203	1.217	1.002	1.112	1.058
752	RPPos	PI(40:6)>PI(18:0_22:6)	4.16	1.180	1.183	1.048	1.044	1.206	1.233	1.009	1.117	1.074
753	RPPos	PS(36:1)	5.14	-1.149	-1.105	-1.063	-1.211	-1.487	-1.127	-1.325	-1.280	-1.331
754	RPNeg	PS(36:1)>PS(18:0_18:1)	5.16	-1.593	-1.218	-1.191	-1.119	-1.385	-1.221	-1.356	-1.759	-1.596
755	RPPos	PS(36:2)	4.52	-1.213	-1.543	-1.301	-1.174	-1.586	-1.395	-1.190	-2.020	-2.455
756	RPPos	PS(36:3)	3.89	1.045	-1.109	-1.289	1.354	-1.639	-1.099	1.136	-1.203	-1.037
757	RPPos	PS(38:2)	1.90	-1.228	1.266	-2.760	-2.476	-2.655	-5.410	-1.306	-1.949	-1.357
758	RPPos	PS(38:3)	2.71	1.263	1.123	-1.603	-1.106	-1.209	-1.402	1.284	1.021	-1.000
759	RPPos	PS(38:3)	2.47	1.022	1.074	-1.762	-1.075	-1.341	-1.878	1.152	1.349	1.260
760	RPPos	PS(38:4)>PS(18:0_20:4)	4.41	1.103	-1.270	-1.297	1.320	-1.523	-1.400	-1.069	-1.909	-2.089
761	RPNeg	PS(38:4)>PS(18:0_20:4)	4.49	-1.108	-1.479	-1.597	1.296	-1.732	-1.347	-1.019	-2.207	-2.287
762	RPNeg	PS(38:5)	3.88	1.045	1.100	-1.035	-1.031	1.110	-1.011	-1.008	1.127	-1.041
763	RPNeg	PS(40:0)	6.13	-1.038	-1.148	-1.074	1.129	1.147	1.000	-1.022	-1.076	-1.158
764	RPPos	PS(40:4)	3.12	-1.040	-1.021	-1.646	-1.404	-1.793	-1.518	1.465	1.527	-1.391
765	RPPos	PS(40:4)	2.71	1.091	1.238	1.030	1.092	-1.409	-1.263	1.560	1.311	1.018
766	RPPos	PS(40:5)	2.47	-1.014	1.153	-1.465	-1.152	-1.216	-1.825	1.546	1.388	1.130
767	RPPos	PS(40:6)	2.71	1.180	-1.111	-1.665	1.199	-1.272	-1.612	1.155	1.012	-1.074
768	RPNeg	PS(40:6)>PS(18:0_22:6)	4.31	1.168	-1.072	-1.022	1.058	-1.174	1.012	-1.009	-1.291	-1.115
769	RPNeg	PS(40:6)>PS(18:0_22:6)	4.32	1.122	1.035	1.005	1.095	1.173	1.207	1.066	1.227	1.231
770	RPPos	PS(40:8)	3.31	1.430	1.459	-1.019	1.250	-1.848	-1.352	1.020	-1.785	1.009
771	RPNeg	PS(40:8)>PS(20:4/20:4)	3.38	1.348	1.316	-1.014	1.331	-1.512	-1.429	1.105	-1.956	1.080
772	RPPos	PS(O-36:2)	2.91	-1.127	-1.099	-1.281	-1.336	-1.796	-1.418	-1.110	-1.594	-1.529
773	RPPos	PS(O-36:2)	3.16	-1.269	1.067	-1.284	-1.232	-1.436	-1.140	-1.155	-1.290	-1.377
774	RPPos	PS(O-36:2)	3.57	-1.040	-1.014	-1.139	-1.014	-1.064	-1.146	-1.005	-1.105	-1.191
775	RPPos	PS(O-36:3)	3.40	1.421	1.366	1.266	1.006	1.294	-1.189	1.122	1.623	-1.017
776	RPPos	PS(O-36:3)	3.01	-1.073	-1.050	-1.227	-1.681	-1.194	-1.220	-1.066	-1.182	-1.238
777	RPPos	PS(O-36:3)	2.93	-1.636	-1.362	-1.782	-1.262	-1.521	-1.500	-1.103	-1.579	-1.214
778	RPNeg	PS(O-42:7)>PS(O-20:3/22:4)	6.21	1.130	-1.124	-1.151	1.075	-1.395	-1.025	-1.037	-1.272	-1.156
779	RPPos	SiE(18:2)	9.40	-1.010	-1.086	-1.013	1.170	1.038	-1.114	1.133	1.286	-1.079
780	RPPos	SiE(20:4)	9.18	1.084	1.105	1.029	1.018	1.344	1.140	1.161	1.354	1.380
781	RPPos	SiE(20:4)	9.18	1.082	1.116	1.026	1.027	1.430	1.140	1.198	1.423	1.420
782	RPPos	SiE(22:6)	8.96	1.256	1.242	1.159	1.084	1.372	1.287	1.237	1.587	1.348
783	RPPos	SM(d30:1)	2.97	-1.001	1.052	-1.061	-1.045	1.148	-1.005	-1.284	1.151	1.065

784	RPPos	SM(d31:0)	2.64	1.019	-1.184	-1.199	-1.272	-1.375	-1.231	-1.080	-1.164	-1.125
785	RPPos	SM(d32:1)	3.41	-1.023	-1.004	-1.136	-1.032	-1.015	-1.042	-1.054	-1.011	-1.020
786	RPPos	SM(d32:2)	3.02	1.037	1.046	-1.010	-1.065	1.071	1.060	-1.052	1.133	-1.009
787	RPPos	SM(d33:0)	3.90	-1.034	1.123	1.161	1.256	1.433	2.008	1.218	1.100	1.175
788	RPPos	SM(d33:1)	3.68	1.001	1.114	-1.003	1.023	1.140	1.020	-1.026	1.115	1.153
789	RPPos	SM(d33:2)	7.44	1.103	1.027	1.224	-1.144	-1.264	1.181	-1.058	1.040	1.136
790	RPPos	SM(d34:0)	4.23	1.049	-1.023	-1.116	1.092	1.300	1.316	1.076	1.476	1.377
791	RPNeg	SM(d34:0)	4.28	1.197	1.037	1.029	1.103	1.304	1.360	1.070	1.267	1.492
792	RPPos	SM(d34:1)	3.98	1.081	1.116	1.019	1.047	1.137	1.156	1.085	1.216	1.296
793	RPNeg	SM(d34:1)	4.03	1.081	1.091	1.057	-1.047	1.139	1.056	-1.018	1.160	1.388
794	RPPos	SM(d34:1-OH)	3.76	-1.008	1.022	1.023	1.104	1.026	-1.016	-1.042	1.012	1.209
795	RPPos	SM(d34:1-OH)	3.22	-1.003	1.008	-1.036	-1.056	1.132	1.039	-1.036	1.095	1.117
796	RPPos	SM(d34:2)	3.49	1.020	1.049	1.001	1.034	1.077	1.055	-1.019	1.153	1.186
797	RPNeg	SM(d34:2)	3.55	1.055	1.064	1.041	-1.008	1.167	1.104	-1.036	1.100	1.230
798	RPPos	SM(d35:0)	4.33	1.190	1.250	1.205	1.311	1.496	1.250	1.141	1.563	2.020
799	RPNeg	SM(d35:1)	4.39	1.112	1.141	1.101	-1.048	1.177	1.111	1.105	1.210	1.471
800	RPPos	SM(d35:1)	4.33	1.077	1.193	1.106	1.147	1.437	1.076	1.035	1.710	1.760
801	RPPos	SM(d35:2)	3.78	1.046	1.017	-1.068	-1.011	1.085	1.060	-1.047	1.091	1.196
802	RPPos	SM(d36:0)	5.02	1.479	1.551	1.108	1.406	1.808	1.707	1.167	2.069	2.121
803	RPPos	SM(d36:1)	4.10	-1.080	-1.061	-1.092	1.082	1.303	1.090	-1.159	1.307	1.451
804	RPPos	SM(d36:1)	4.71	-1.009	1.149	-1.037	1.096	1.393	1.327	-1.033	1.487	1.653
805	RPNeg	SM(d36:1)	4.76	1.073	1.192	1.051	1.027	1.372	1.155	-1.074	1.537	1.851
806	RPPos	SM(d36:2)	4.10	1.055	1.126	-1.002	1.035	1.247	1.023	-1.038	1.384	1.508
807	RPNeg	SM(d36:2)	4.15	1.070	1.097	1.040	1.048	1.318	1.093	1.017	1.420	1.553
808	RPPos	SM(d36:3)	3.61	-1.026	1.192	1.036	1.278	1.584	1.222	-1.056	1.632	1.631
809	RPPos	SM(d36:4)	4.00	-1.107	1.041	1.016	1.027	1.056	1.076	1.034	1.067	-1.050
810	RPPos	SM(d37:1)	5.13	1.040	1.124	-1.011	1.066	1.485	1.216	1.007	1.793	2.099
811	RPPos	SM(d37:2)	4.47	1.193	1.479	1.322	1.082	1.410	1.195	-1.046	1.687	2.289
812	RPPos	SM(d38:0)	5.91	-1.036	1.152	-1.047	-1.021	1.097	1.250	-1.174	1.353	1.570
813	RPPos	SM(d38:1)	5.58	1.094	1.196	1.055	1.070	1.341	1.154	1.020	1.456	1.606
814	RPNeg	SM(d38:1)	5.63	1.133	1.257	1.091	1.024	1.352	1.051	1.010	1.527	1.712
815	RPPos	SM(d38:2)	4.87	1.043	1.019	-1.002	1.036	1.175	1.144	1.042	1.014	1.288
816	RPPos	SM(d38:2)	4.74	1.173	1.360	1.014	-1.038	1.310	1.133	-1.117	1.399	1.448
817	RPNeg	SM(d38:2)	4.92	1.052	1.083	1.003	1.023	1.168	1.239	-1.101	1.229	1.434
818	RPNeg	SM(d38:2)	4.79	1.016	1.357	-1.057	1.061	1.686	1.114	-1.171	1.746	2.197
819	RPPos	SM(d38:3)	4.17	1.143	1.533	1.295	1.028	1.664	-1.014	-1.187	2.195	2.052
820	RPPos	SM(d38:4)	3.76	-1.023	1.068	-1.269	-1.234	-1.086	-1.285	-1.226	1.188	-1.463

821	RPPos	SM(d39:0)	6.21	-1.012	-1.014	-1.188	-1.051	-1.021	-1.233	1.014	1.015	-1.051
822	RPPos	SM(d39:1)	6.05	1.040	1.234	1.029	1.046	1.312	1.157	1.048	1.427	1.441
823	RPNeg	SM(d39:1)	6.10	1.121	1.189	1.020	1.064	1.399	1.350	1.013	1.452	1.587
824	RPPos	SM(d39:2)	5.13	1.144	1.185	1.051	-1.018	1.369	1.216	-1.081	1.392	1.326
825	RPPos	SM(d39:2)	5.32	1.057	1.118	1.076	-1.022	1.343	1.198	1.023	1.310	1.619
826	RPPos	SM(d40:0)	6.62	1.035	1.046	-1.073	-1.074	1.256	1.140	-1.092	1.297	1.346
827	RPNeg	SM(d40:0-OH)	6.89	1.123	1.177	1.031	1.030	1.168	1.114	-1.010	1.127	1.074
828	RPPos	SM(d40:1)	6.44	1.057	1.212	1.017	1.020	1.264	1.063	1.020	1.446	1.223
829	RPPos	SM(d40:1)	5.89	1.027	1.153	-1.055	-1.115	1.209	1.450	-1.101	1.159	1.792
830	RPNeg	SM(d40:1)	6.50	1.145	1.179	1.042	1.051	1.209	1.081	-1.033	1.241	1.278
831	RPNeg	SM(d40:1)	5.93	1.011	1.168	-1.028	-1.023	1.365	1.319	-1.135	1.228	2.220
832	RPPos	SM(d40:2)	5.55	1.055	1.207	-1.043	1.020	1.279	1.150	-1.037	1.231	1.567
833	RPPos	SM(d40:2)	5.78	1.026	1.134	-1.054	-1.008	1.186	-1.008	-1.005	1.266	1.161
834	RPNeg	SM(d40:2)	5.60	1.025	1.221	-1.001	1.063	1.378	1.226	-1.099	1.247	1.552
835	RPNeg	SM(d40:2)	5.83	1.070	1.151	-1.061	-1.008	1.175	1.018	-1.044	1.173	1.187
836	RPPos	SM(d40:3)	4.85	1.025	1.089	1.072	1.100	1.183	1.117	-1.011	1.040	1.280
837	RPPos	SM(d40:4)	4.33	1.146	1.368	-1.257	1.095	1.885	1.075	-1.012	1.676	1.709
838	RPPos	SM(d41:0)	6.80	1.007	1.071	1.060	1.048	1.147	-1.113	-1.391	1.073	1.123
839	RPPos	SM(d41:1)	6.68	1.097	1.283	1.048	1.023	1.327	1.294	1.046	1.569	1.334
840	RPNeg	SM(d41:1)	6.74	1.101	1.157	1.022	1.024	1.281	1.206	-1.004	1.363	1.288
841	RPPos	SM(d41:2)	6.23	1.012	1.178	1.003	-1.014	1.244	1.012	1.011	1.373	1.235
842	RPPos	SM(d41:2)	5.98	1.051	1.188	-1.011	-1.010	1.272	1.117	1.003	1.308	1.322
843	RPNeg	SM(d41:2)	6.28	1.048	1.201	-1.019	-1.003	1.244	1.047	-1.008	1.269	1.250
844	RPNeg	SM(d41:2)	6.03	1.090	1.221	-1.011	1.002	1.281	1.071	1.001	1.315	1.349
845	RPPos	SM(d41:3)	4.38	1.255	1.340	1.219	1.084	1.161	1.249	1.103	1.344	1.501
846	RPPos	SM(d41:3)	5.46	1.082	-1.035	-1.179	1.171	1.294	1.060	1.035	1.208	1.255
847	RPPos	SM(d42:0)	6.92	1.045	1.060	-1.049	1.040	1.169	1.097	1.014	1.184	1.270
848	RPPos	SM(d42:1)	6.83	1.060	1.164	1.022	1.053	1.272	1.051	1.044	1.306	1.226
849	RPPos	SM(d42:1)	6.59	1.021	1.070	-1.037	-1.108	1.284	1.245	-1.046	1.279	1.318
850	RPNeg	SM(d42:1)	6.89	1.068	1.130	1.031	-1.020	1.170	1.059	1.005	1.272	1.242
851	RPPos	SM(d42:2)	6.56	-1.095	1.122	-1.048	-1.118	-1.154	1.088	-1.063	1.362	1.297
852	RPPos	SM(d42:2)	6.38	1.045	1.212	-1.020	-1.016	1.258	1.065	1.007	1.374	1.303
853	RPNeg	SM(d42:2)	6.43	1.074	1.181	1.016	1.016	1.216	1.068	-1.040	1.194	1.213
854	RPNeg	SM(d42:2)	6.62	-1.025	1.088	-1.019	-1.007	1.172	1.015	-1.088	1.180	1.067
855	RPPos	SM(d42:3)	5.69	-1.002	1.116	-1.065	1.031	1.161	-1.054	-1.043	1.214	1.115
856	RPNeg	SM(d42:3)	5.74	1.030	1.090	-1.103	-1.060	1.157	1.002	-1.049	1.140	1.161
857	RPPos	SM(d42:4)	4.98	1.005	1.118	1.059	1.331	1.524	1.197	1.024	1.062	1.225
858	RPPos	SM(d42:5)	4.51	1.038	1.285	-1.153	1.120	1.400	1.296	1.001	1.273	2.548

859	RPNeg	SM(d42:5)	4.58	1.055	1.414	-1.066	1.003	1.409	1.447	-1.248	1.446	2.262
860	RPPos	SM(d43:1)	6.91	1.171	1.222	-1.006	1.162	1.254	1.185	1.160	1.345	1.335
861	RPNeg	SM(d43:1)	6.98	1.112	1.199	1.062	1.129	1.292	1.232	1.029	1.291	1.342
862	RPPos	SM(d43:2)	6.67	1.066	1.212	1.048	-1.037	1.260	1.150	1.056	1.400	1.352
863	RPPos	SM(d43:3)	5.14	1.128	1.262	-1.116	1.059	1.266	1.038	-1.083	1.325	-1.158
864	RPPos	SM(d43:3)	6.45	1.201	1.143	-1.105	-1.135	1.343	1.177	1.040	1.403	1.114
865	RPPos	SM(d44:1)	7.09	1.049	1.017	1.090	1.003	1.104	1.278	-1.001	1.176	1.401
866	RPNeg	SM(d44:1)	7.16	1.169	1.127	1.082	1.149	1.166	1.258	1.030	1.192	1.490
867	RPPos	SM(d44:2)	6.66	1.020	1.107	-1.448	-1.342	1.172	1.266	-1.131	1.165	-1.026
868	RPPos	SM(d44:2)	6.80	1.063	1.194	1.109	-1.006	1.261	1.342	1.068	1.401	1.507
869	RPNeg	SM(d44:2)	6.86	1.080	1.190	1.152	-1.036	1.149	1.211	-1.001	1.160	1.521
870	RPPos	SM(d44:3)	6.50	-1.043	1.114	1.010	-1.037	1.154	1.073	-1.024	1.265	1.379
871	RPPos	SM(d44:4)	5.84	-1.161	-1.012	-1.019	1.099	1.035	-1.029	-1.169	1.096	1.188
872	RPPos	sn-glycero-3-phosphocholine	0.74	-1.024	1.122	1.234	-1.142	-1.374	1.142	-1.237	-1.286	1.134
873	RPPos	sn-glycero-3-phosphocholine	0.86	-1.243	1.017	1.019	1.106	-1.189	1.358	-1.145	-1.436	1.316
874	RPPos	sphinganine (C18)	1.84	1.263	-1.006	1.242	1.435	-1.206	1.185	1.159	-1.281	-1.136
875	RPPos	sphingosine (C18)	1.70	1.153	1.027	1.023	1.026	-1.220	-1.053	-1.029	-1.042	-1.508
876	RPPos	taurocholate	0.93	1.130	-1.798	-2.570	1.342	-1.280	-2.896	-1.771	-1.740	-1.889
877	RPPos	tauroursodeoxycholate	0.97	1.181	-1.524	-2.223	1.257	1.207	-2.151	-1.792	-1.188	-1.859
878	RPPos	TG(32:0)	5.83	1.356	1.293	-1.209	-1.029	-1.002	1.095	1.001	-1.032	1.071
879	RPPos	TG(34:0)	6.58	1.523	-1.040	1.029	1.053	1.104	1.071	-1.279	-1.010	1.071
880	RPPos	TG(34:0)	5.83	1.108	1.257	-1.137	-1.214	-1.042	-1.125	-1.586	-1.544	-1.323
881	RPPos	TG(36:0)>TG(10:0_12:0_14:0) and TG(10:0_10:0_16:0)	6.89	1.207	1.238	-1.132	1.012	1.043	1.180	1.247	1.167	1.275
882	RPPos	TG(36:3)	6.58	1.399	1.230	1.069	1.291	1.213	1.131	1.129	1.028	1.207
883	RPPos	TG(38:0)	6.88	1.873	1.275	1.126	1.133	1.244	1.023	1.027	1.126	1.287
884	RPPos	TG(40:0)	7.46	-1.453	1.108	-1.147	-1.316	-1.370	-1.178	-1.677	-1.647	-1.234
885	RPPos	TG(40:5)	4.33	1.005	-1.109	-1.145	-1.112	-1.519	-1.169	-1.296	-1.415	-1.289
886	RPPos	TG(41:2)	6.94	-1.141	-1.016	-1.121	-1.044	-1.054	-1.070	-1.147	-1.027	-1.018
887	RPPos	TG(42:0)>TG(10:0_16:0_16:0) and TG(12:0_14:0_16:0)	7.76	-2.238	-1.317	-1.631	-2.032	-2.067	-1.395	-2.544	-4.600	-2.270
888	RPPos	TG(42:1)	7.48	-2.617	-2.220	-1.994	-1.712	-2.291	-1.414	-2.869	-4.900	-5.040
889	RPPos	TG(42:2)	7.24	-2.990	-2.426	-1.861	-2.565	-3.064	-1.085	-4.039	-5.031	-6.204
890	RPPos	TG(43:0)	7.15	1.299	1.198	-1.096	-1.025	1.446	1.291	1.137	-1.082	1.050
891	RPPos	TG(43:1)	7.62	-2.740	-2.081	-2.730	-2.313	-1.931	-1.211	-3.146	-4.734	-5.524
892	RPPos	TG(43:2)	7.37	-3.180	-2.123	-2.196	-2.339	-2.397	1.146	-3.962	-11.814	-6.482
893	RPPos	TG(44:1)>TG(10:0_16:0_18:1)	7.77	-2.624	-2.394	-2.036	-2.003	-2.404	-1.507	-2.970	-6.680	-5.739
894	RPPos	TG(44:2)	7.51	-2.739	-2.269	-1.814	-2.262	-1.994	-1.165	-3.690	-4.717	-5.588

895	RPPos	TG(44:3)>TG(8:0_18:1_18:2) and TG(10:0_16:1_18:2)	7.27	-3.063	-1.415	-1.806	-2.366	-2.290	-2.386	-3.250	-7.210	-3.007
896	RPPos	TG(44:4)	7.04	-3.707	-2.051	-2.229	-4.980	-3.972	-1.506	-6.066	-7.697	-7.386
897	RPPos	TG(45:0)	8.26	-1.577	-1.109	-1.234	-1.347	-1.123	1.031	-1.576	-1.506	-1.410
898	RPPos	TG(45:2)	7.66	-3.665	-2.114	-2.204	-1.339	-1.930	-1.497	-3.202	-7.962	-5.026
899	RPPos	TG(45:3)	7.39	-3.676	-3.246	-1.743	-1.322	-2.280	1.277	-5.041	-6.126	-5.391
900	RPPos	TG(46:0)>TG(14:0_16:0_16:0) and TG(12:0_16:0_18:0)	8.44	-1.504	1.111	-1.018	-1.548	-1.468	1.077	-1.320	-1.806	-1.249
901	RPPos	TG(46:0)>TG(14:0_16:0_16:0) and TG(12:0_16:0_18:0)	8.35	-1.797	-1.292	-1.226	1.091	-1.082	-1.046	1.231	1.009	1.033
902	RPPos	TG(46:1)>TG(12:0_16:0_18:1) and TG(10:0_18:0_18:1) and TG(14:0_16:0_16:1)	8.08	-2.569	-2.584	-1.846	-1.753	-2.582	-1.534	-2.532	-4.683	-4.800
903	RPPos	TG(46:2)	7.80	-2.642	-2.639	-1.831	-1.909	-2.450	-1.486	-3.052	-5.783	-5.682
904	RPPos	TG(46:2)	7.79	-2.637	-2.634	-1.826	-1.907	-2.447	-1.485	-3.054	-5.760	-5.657
905	RPPos	TG(46:3)>TG(10:0_18:1_18:2) and TG(12:0_16:1_18:2)	7.51	-2.986	-2.507	-2.474	-3.999	-3.166	-1.998	-3.793	-10.817	-7.093
906	RPPos	TG(46:3)>TG(10:0_18:1_18:2) and TG(12:0_16:1_18:2)	7.54	-2.822	-2.321	-2.474	-4.304	-2.513	-1.846	-3.196	-3.954	-3.094
907	RPPos	TG(46:4)>TG(10:0_18:2_18:2)	7.28	-3.520	-2.096	-2.468	-2.934	-2.236	-1.336	-5.936	-10.106	-6.010
908	RPPos	TG(46:5)	7.12	-3.736	-3.209	-1.926	-3.498	-2.985	-1.362	-5.702	-5.504	-4.591
909	RPPos	TG(47:0)	8.56	-1.717	-1.238	-1.138	-1.092	-1.259	1.023	-1.735	-2.049	-1.764
910	RPPos	TG(47:1)	8.20	-2.225	-2.196	-1.710	-1.524	-1.937	-1.490	-1.977	-4.251	-2.506
911	RPPos	TG(47:2)	7.97	-2.243	-1.641	-1.743	-1.607	-1.888	-1.366	-2.485	-3.853	-3.220
912	RPPos	TG(47:2)	7.24	-2.894	-1.270	-1.851	-1.565	-2.134	-1.752	-2.509	-2.667	-2.752
913	RPPos	TG(48:0)>TG(16:0/16:0/16:0) and TG(14:0_16:0_18:0)	8.84	1.419	1.345	1.460	1.052	-1.060	1.011	1.598	1.568	1.459
914	RPPos	TG(48:1)>TG(16:0_16:0_16:1) and TG(14:0_16:0_18:1)	8.43	-2.082	-2.360	-1.571	-1.739	-2.331	-1.375	-1.874	-3.387	-3.492
915	RPPos	TG(48:2)>TG(14:0_16:0_18:2) and TG(14:0_16:1_18:1)	8.13	-2.090	-2.315	-1.594	-1.800	-2.398	-1.464	-2.317	-2.933	-3.710
916	RPPos	TG(48:3)	7.81	-2.314	-3.792	-1.820	-2.133	-2.634	-1.397	-2.120	-4.554	-5.798
917	RPPos	TG(48:3)	7.89	-2.154	-2.233	-1.297	-3.033	-2.800	-1.162	-3.039	-3.489	-2.634
918	RPPos	TG(48:4)>TG(12:0_18:2_18:2) and TG(14:1_16:1_18:2)	7.58	-2.934	-1.944	-2.205	-1.844	-2.424	-1.210	-2.566	-2.887	-2.668
919	RPPos	TG(48:4)>TG(12:0_18:2_18:2) and TG(14:1_16:1_18:2)	7.68	-2.513	-2.321	-1.229	-2.081	-2.449	-1.421	-2.858	-2.005	-2.987
920	RPPos	TG(48:6)	7.21	-3.535	-2.660	-1.843	-2.673	-3.269	-1.411	-3.568	-3.527	-5.941

921	RPPos	TG(49:0)>TG(16:0_16:0_17:0) and TG(15:0_16:0_18:0) and TG(14:0_16:0_19:0)	8.97	-1.169	1.032	1.194	1.072	-1.148	1.100	-1.216	-1.360	-1.209
922	RPPos	TG(49:1)>TG(15:0_16:0_18:0) and TG(16:0_16:0_17:1) and TG(16:0_16:1_17:0)	8.63	-1.737	-1.727	-1.405	-1.583	-2.267	-1.221	-1.984	-2.670	-2.341
923	RPPos	TG(49:1)>TG(15:0_16:0_18:0) and TG(16:0_16:0_17:1) and TG(16:0_16:1_17:0)	7.77	-3.326	-2.647	-1.982	-2.500	-2.768	-1.469	-3.898	-4.475	-8.663
924	RPPos	TG(49:2)	8.30	-1.887	-1.811	-1.476	-1.566	-2.582	-1.247	-2.108	-2.784	-2.509
925	RPPos	TG(49:2)	8.41	1.168	4.455	1.118	1.099	1.451	1.150	8.291	13.787	12.332
926	RPPos	TG(49:4)	7.86	-2.056	-1.492	-1.780	-2.834	-1.856	-1.494	-2.113	-2.197	-2.824
927	RPPos	TG(49:4)	7.74	-2.289	-2.109	-1.769	-1.434	-2.246	-1.113	-2.742	-2.718	-2.987
928	RPPos	TG(50:0)>TG(16:0_16:0_18:0)	9.25	1.686	1.373	1.482	1.057	-1.085	-1.088	1.837	2.014	1.581
929	RPPos	TG(50:0)>TG(16:0_16:0_18:0)	9.13	-1.052	-1.176	1.247	1.146	1.071	1.148	-1.611	-1.467	-1.219
930	RPPos	TG(50:1)>TG(16:0_16:0_18:1)	8.83	-1.516	-1.733	-1.347	-1.436	-1.943	-1.213	-1.491	-1.823	-1.791
931	RPPos	TG(50:2)>TG(16:0_16:0_18:2) and TG(16:0_16:1_18:1)	8.50	-1.536	-1.321	-1.205	-1.414	-1.551	-1.073	-1.621	-1.276	-1.183
932	RPPos	TG(50:2)>TG(16:0_16:0_18:2) and TG(16:0_16:1_18:1)	8.43	-1.425	-2.580	-1.600	-1.261	-3.165	-1.624	-1.345	-2.967	-4.457
933	RPPos	TG(50:2)>TG(16:0_16:0_18:2) and TG(16:0_16:1_18:1)	8.43	-1.023	1.010	-1.129	-1.266	-2.008	-1.033	-1.386	-1.879	-1.996
934	RPPos	TG(50:3)	8.01	-2.165	-1.983	-1.625	-1.826	-2.505	-1.199	-2.467	-1.986	-2.566
935	RPPos	TG(50:3)	8.13	-1.407	-1.888	-1.522	-1.334	-2.259	-1.376	-1.444	-2.134	-2.336
936	RPPos	TG(50:4)>TG(16:1_16:1_18:2)	7.64	-2.728	-2.925	-1.678	-1.968	-2.829	-1.429	-2.689	-3.683	-5.064
937	RPPos	TG(50:4)>TG(16:1_16:1_18:2) and TG(16:1_16:1_18:2)	7.85	-1.639	-2.451	-1.497	-1.472	-2.465	-1.480	-1.995	-2.542	-3.586
938	RPPos	TG(50:4)>TG(16:1_16:1_18:2) and TG(16:1_16:1_18:2)	7.86	-1.635	-2.403	-1.489	-1.494	-2.406	-1.469	-1.965	-2.574	-3.532
939	RPPos	TG(50:6)	7.49	-2.395	-2.706	-1.801	-1.962	-2.847	-1.589	-2.988	-3.273	-4.122
940	RPPos	TG(51:0)	9.34	-1.151	1.330	1.443	-1.018	1.386	1.541	1.168	-1.032	1.202
941	RPPos	TG(51:1)	8.94	-1.829	-1.498	-1.222	-1.855	-3.004	-1.173	-2.337	-4.118	-2.638
942	RPPos	TG(51:1)	9.03	-1.911	-1.869	-1.394	-1.615	-2.231	-1.369	-1.833	-2.411	-1.769
943	RPPos	TG(51:2)	8.69	-1.955	-1.406	-1.079	-1.451	-1.728	1.005	-1.480	-1.060	4.612
944	RPPos	TG(51:2)>TG(15:0_18:1_18:1) and TG(16:0_17:1_18:1) and TG(16:1_17:0_18:1)	8.62	-1.393	-1.914	-1.440	-1.399	-2.967	-1.272	-1.581	-3.123	-3.534
945	RPPos	TG(51:3)>TG(15:0_18:1_18:2) and TG(16:0_17:1_18:2) and TG(16:1_17:1_18:1)	8.30	-1.427	-1.605	-1.337	-1.281	-2.388	-1.274	-1.510	-2.139	-2.425

946	RPPos	TG(51:4)>TG(16:1_17:1_18:2) and TG(15:0_18:2_18:2) and TG(15:1_18:1_18:2)	8.01	-1.535	-1.709	-1.350	-1.346	-2.234	-1.225	-1.791	-2.293	-2.248
947	RPPos	TG(51:4)>TG(16:1_17:1_18:2) and TG(15:0_18:2_18:2) and TG(15:1_18:1_18:2)	7.78	-1.846	-1.371	-1.530	-1.796	-3.446	-1.604	-1.883	-2.483	-2.157
948	RPPos	TG(51:5)	7.87	-1.113	-1.116	-1.189	-1.353	-1.130	1.290	-1.138	-1.324	1.033
949	RPPos	TG(51:6)	7.62	-1.866	-2.633	-1.685	-1.082	-1.696	1.019	-2.073	-2.723	-3.313
950	RPPos	TG(52:0)>TG(16:0_16:0_20:0)	9.49	1.179	1.385	1.429	-1.035	-1.043	-1.141	1.506	1.537	1.259
951	RPPos	TG(52:1)	9.23	-1.568	-1.699	-1.498	-1.700	-1.766	-1.118	-1.468	-1.819	-1.636
952	RPPos	TG(52:1)	9.23	-1.564	-1.700	-1.495	-1.688	-1.782	-1.119	-1.459	-1.812	-1.625
953	RPPos	TG(52:2)>TG(16:0_18:1_18:1)	8.81	-1.216	-1.346	-1.232	-1.175	-2.291	-1.272	-1.136	-1.707	-1.856
954	RPPos	TG(52:2)>TG(16:0_18:1_18:1)	8.81	-1.222	-1.338	-1.243	-1.159	-2.312	-1.308	-1.101	-1.709	-1.816
955	RPPos	TG(52:3)>TG(16:0_18:1_18:2) and TG(16:1_18:1_18:1)	8.37	-1.408	-1.021	1.015	-1.716	-1.401	1.144	-1.401	1.367	1.328
956	RPPos	TG(52:3)>TG(16:0_18:1_18:2) and TG(16:1_18:1_18:1)	8.49	-1.078	-1.280	-1.188	-1.024	-1.641	-1.227	-1.152	-1.245	-1.353
957	RPPos	TG(52:3)>TG(16:0_18:1_18:2) and TG(16:1_18:1_18:1)	8.49	-1.081	-1.305	-1.197	-1.027	-1.676	-1.246	-1.163	-1.271	-1.381
958	RPPos	TG(52:3)>TG(16:0_18:1_18:2) and TG(16:1_18:1_18:1)	8.33	-1.484	-2.479	-1.688	-1.477	-4.570	-1.579	-1.684	-3.210	-3.133
959	RPPos	TG(52:4)>TG(16:0_16:0_20:4) and TG(16:1_18:1_18:2) and TG(16:0_18:1_18:2)	8.37	-1.788	-1.154	-1.144	-1.638	-1.417	1.088	-1.518	-1.045	1.201
960	RPPos	TG(52:4)>TG(16:1_18:1_18:2)	8.18	-1.026	-1.269	-1.170	-1.010	-2.049	-1.273	-1.136	-1.184	-1.259
961	RPPos	TG(52:5)>TG(16:0_18:2_18:3)	7.94	-1.374	-1.896	-1.376	-1.178	-2.282	-1.651	-1.364	-1.681	-2.370
962	RPPos	TG(52:5)>TG(16:0_18:2_18:3)	7.94	-1.376	-1.896	-1.376	-1.178	-2.282	-1.650	-1.364	-1.680	-2.374
963	RPPos	TG(52:5)>TG(16:0_18:2_18:3)	7.94	-1.382	-1.903	-1.373	-1.180	-2.282	-1.653	-1.360	-1.688	-2.378
964	RPPos	TG(52:6)	7.75	-1.597	-2.312	-1.630	-1.531	-2.479	-1.457	-1.787	-2.190	-2.595
965	RPPos	TG(52:7)	7.52	-2.163	-2.350	-1.515	-1.599	-2.096	-1.341	-2.174	-2.171	-2.265
966	RPPos	TG(52:7)	7.54	-2.190	-2.355	-1.517	-1.606	-2.128	-1.341	-2.189	-2.233	-2.323
967	RPPos	TG(53:0)	9.54	1.166	1.577	1.489	1.310	1.214	1.270	1.165	-1.037	1.135
968	RPPos	TG(53:1)>TG(16:0_18:1_19:0) and TG(17:0_18:0_18:1)	9.37	-1.721	-1.394	-1.399	-1.729	-1.579	1.091	-1.632	-1.731	-1.560
969	RPPos	TG(53:2)	9.01	-1.606	-1.790	-1.318	-1.368	-2.929	-1.273	-1.481	-2.694	-2.237
970	RPPos	TG(53:3)>TG(17:0_18:1_18:2) and TG(17:1_18:1_18:1) and TG(16:1_18:1_19:1) and TG(16:0_18:1_19:2)	8.68	-1.496	-1.572	-1.207	-1.373	-2.077	-1.375	-1.451	-2.046	-2.223

971	RPPos	TG(53:3)>TG(17:0_18:1_18:2) and TG(17:1_18:1_18:1) and TG(16:1_18:1_19:1) and TG(16:0_18:1_19:2)	8.60	-1.401	-2.020	-1.395	-1.309	-2.398	-1.324	-1.815	-3.948	-3.017
972	RPPos	TG(53:3)>TG(17:0_18:1_18:2) and TG(17:1_18:1_18:1) and TG(16:1_18:1_19:1) and TG(16:0_18:1_19:2)	8.89	-1.439	-3.022	-2.069	-1.881	-2.141	-1.919	-2.107	-1.650	-2.213
973	RPPos	TG(53:4)>TG(17:0_18:2_18:2) and TG(17:1_18:1_18:2)	8.33	-1.312	-1.594	-1.455	-1.417	-2.456	-1.654	-1.360	-2.363	-1.846
974	RPPos	TG(53:5)>TG(17:1_18:2_18:2)	8.02	-1.479	-2.352	-1.697	-1.256	-2.020	-1.604	-1.788	-2.292	-3.077
975	RPPos	TG(53:5)>TG(17:1_18:2_18:2)	8.17	-1.390	-1.064	-1.422	1.120	1.018	1.427	-1.475	-1.053	-1.015
976	RPPos	TG(53:6)	8.06	-1.847	-1.349	-1.266	-1.249	-1.834	-1.094	-1.762	-1.173	-1.794
977	RPPos	TG(53:7)	7.69	-1.441	-1.533	-1.226	-1.374	-1.808	-1.046	-1.749	-1.527	-1.909
978	RPPos	TG(54:0)	9.63	-1.183	1.151	1.232	-1.066	-1.157	1.090	1.153	-1.094	1.027
979	RPPos	TG(54:1)>TG(18:0_18:0_18:1) and TG(16:1_18:0_20:0) and TG(16:0_18:0_20:1)	9.47	-1.519	-1.074	-1.180	-1.759	-1.579	-1.029	-1.596	-1.649	-1.330
980	RPPos	TG(54:2)>TG(16:0_18:1_20:1) and TG(18:0_18:1_18:1)	8.87	-1.655	-1.909	-1.497	-1.443	-2.171	-1.355	-1.881	-2.047	-2.108
981	RPPos	TG(54:2)>TG(16:0_18:1_20:1) and TG(18:0_18:1_18:1)	9.21	-1.810	-2.041	-1.692	-1.602	-2.496	-1.389	-2.050	-3.381	-2.010
982	RPPos	TG(54:2)>TG(16:0_18:1_20:1) and TG(18:0_18:1_18:1)	9.20	-1.685	-2.047	-1.674	-1.575	-2.348	-1.339	-1.839	-3.010	-1.985
983	RPPos	TG(54:2)>TG(16:0_18:1_20:1) and TG(18:0_18:1_18:1)	9.21	-1.802	-2.065	-1.679	-1.585	-2.588	-1.390	-2.053	-3.408	-2.004
984	RPPos	TG(54:3)>TG(18:0_18:1_18:2) and TG(18:1/18:1/18:1) and TG(16:0_18:2_20:1)	8.88	-1.506	-1.445	-1.243	-1.434	-2.158	-1.443	-1.666	-2.356	-1.958
985	RPPos	TG(54:3)>TG(18:0_18:1_18:2) and TG(18:1/18:1/18:1) and TG(16:0_18:2_20:1)	8.87	-1.606	-1.977	-1.507	-1.264	-2.207	-1.344	-1.640	-2.336	-2.107
986	RPPos	TG(54:4)>TG(18:1_18:1_18:2)	8.56	-1.350	-1.206	-1.177	-1.518	-2.538	-1.056	-1.981	-1.705	-1.877
987	RPPos	TG(54:4)>TG(18:1_18:1_18:2)	8.49	-1.656	-1.232	-1.553	-1.284	-1.504	-1.120	1.045	-1.588	-1.584
988	RPPos	TG(54:4)>TG(18:1_18:1_18:2)	8.48	-1.675	-1.223	-1.564	-1.219	-1.427	-1.220	-1.368	-1.901	-1.629
989	RPPos	TG(54:5)>TG(18:1_18:2_18:2)	8.18	-1.691	-1.511	-1.499	-1.435	-1.795	-1.286	-2.376	-2.562	-1.787
990	RPPos	TG(54:5)>TG(18:1_18:2_18:2)	8.18	-1.692	-1.483	-1.497	-1.437	-1.748	-1.277	-2.300	-2.530	-1.761
991	RPPos	TG(54:5)>TG(18:1_18:2_18:2)	8.09	1.221	1.125	1.422	1.053	-1.119	-1.043	-1.023	1.546	-1.219

992	RPPos	TG(54:5)>TG(18:1_18:2_18:2) and TG(16:0_16:0_22:5) and TG(16:0_18:1_20:4)	8.36	- 1.168	- 1.149	- 1.221	-1.004	-1.492	-1.095	-1.011	1.102	-1.145
993	RPPos	TG(54:6)>TG(16:0_16:0_22:6)	8.24	- 1.272	- 1.176	- 1.303	-1.490	-1.876	-1.139	-2.425	-2.843	-1.408
994	RPPos	TG(54:6)>TG(16:0_18:2_20:4)	8.08	1.063	- 1.066	1.076	-1.106	-1.364	-1.095	1.034	1.576	-1.098
995	RPPos	TG(54:6)>TG(16:0_18:2_20:4)	8.11	1.185	- 1.027	1.046	1.037	-1.370	-1.055	1.039	1.235	-1.407
996	RPPos	TG(54:6)>TG(16:0_18:2_20:4)	8.09	1.065	1.078	1.093	1.016	-1.355	-1.067	1.173	1.592	1.096
997	RPPos	TG(54:6)>TG(18:2/18:2/18:2)	7.90	- 2.064	- 1.737	- 1.607	-1.654	-2.206	-1.638	-1.884	-2.107	-1.581
998	RPPos	TG(54:6)>TG(18:2/18:2/18:2) and TG(18:1_18:2_18:3)	7.69	- 1.661	- 1.518	- 1.316	-1.849	-1.951	-1.137	-2.127	-1.943	-2.184
999	RPPos	TG(54:6)>TG(18:2/18:2/18:2) and TG(18:1_18:2_18:3)	7.63	- 1.174	- 1.153	- 1.064	-1.176	-1.544	1.030	-1.468	-1.136	-1.878
1000	RPPos	TG(54:7)>TG(16:0_16:1_22:6) and TG(16:0_18:2_20:5)	7.84	- 1.045	- 1.309	- 1.008	1.003	-1.540	-1.115	-1.056	1.067	-1.612
1001	RPPos	TG(54:7)>TG(18:2_18:2_18:3)	7.68	- 2.786	- 2.036	- 1.491	-1.463	-3.074	-1.310	-2.278	-1.922	-2.268
1002	RPPos	TG(54:7)>TG(18:2_18:2_18:3)	7.68	- 2.647	- 2.043	- 1.508	-1.452	-2.995	-1.304	-2.197	-1.973	-2.254
1003	RPPos	TG(54:8)	7.63	- 1.387	- 1.489	- 1.174	-1.269	-1.273	-1.095	-1.530	-1.396	-2.028
1004	RPPos	TG(54:8)	7.52	- 1.543	- 1.546	- 1.138	-1.403	-1.787	-1.385	-1.832	-1.550	-2.246
1005	RPPos	TG(54:9)	7.43	- 1.950	- 1.243	- 1.132	-1.341	-1.447	-1.269	-1.805	-1.389	-1.402
1006	RPPos	TG(55:1)>TG(18:0_18:1_19:0) and TG(16:0_18:1_21:0)	9.54	- 1.871	- 1.081	- 1.068	-1.626	-1.399	-1.026	-1.720	-2.413	-1.248
1007	RPPos	TG(55:2)	8.43	- 1.739	- 2.045	- 1.800	-1.366	-2.962	-1.758	-1.377	-3.759	-3.358
1008	RPPos	TG(55:2)	9.37	- 2.016	- 1.544	- 1.472	-1.916	-2.131	-1.170	-2.150	-3.498	-2.025
1009	RPPos	TG(55:2)	8.81	1.039	- 1.277	- 1.011	1.013	-1.232	1.118	1.034	-1.077	-1.078
1010	RPPos	TG(55:2)	8.51	- 1.054	- 1.067	1.005	-1.178	-1.271	1.073	-1.303	1.065	-1.016
1011	RPPos	TG(55:3)>TG(18:1_18:1_19:1) and TG(18:1_18:2_19:0)	9.02	- 1.435	- 1.533	- 1.363	-1.573	-2.923	-1.191	-1.903	-3.073	-2.115
1012	RPPos	TG(55:4)>TG(18:1_18:2_19:1) and TG(18:2_18:2_19:0) and TG(18:1_18:1_19:2)	8.67	- 1.822	- 1.848	- 1.664	-1.282	-1.970	-1.292	-1.745	-2.383	-2.025
1013	RPPos	TG(55:4)>TG(18:1_18:2_19:1) and TG(18:2_18:2_19:0) and TG(18:1_18:1_19:2)	8.67	- 1.822	- 1.795	- 1.552	-1.281	-2.093	-1.432	-1.744	-2.400	-2.024
1014	RPPos	TG(55:5)>TG(18:2_18:2_19:1) and TG(18:1_18:2_19:2)	7.64	- 2.288	- 1.067	- 1.599	-1.743	-2.463	-1.354	-2.471	-4.301	-2.826
1015	RPPos	TG(55:6)	8.23	- 1.270	- 1.199	- 1.047	-1.233	-1.457	1.025	-1.222	-1.338	-1.648
1016	RPPos	TG(55:7)	7.94	- 1.170	- 1.318	- 1.057	-1.163	-1.309	-1.048	-1.186	-1.224	-1.551
1017	RPPos	TG(55:8)	7.78	- 1.300	- 1.094	- 1.004	-1.104	-1.281	1.231	-1.700	-1.428	-1.483
1018	RPPos	TG(56:0)	9.25	1.484	1.173	1.227	-1.007	-1.128	-1.367	1.142	1.177	1.079

1019	RPPos	TG(56:0)	9.77	-1.661	-1.240	-1.260	-1.342	-1.300	1.350	-1.444	-1.553	-1.151
1020	RPPos	TG(56:1)	9.62	-1.736	-1.016	-1.027	-1.725	-1.270	1.031	-1.812	-2.601	-1.112
1021	RPPos	TG(56:10)	7.53	-1.873	-1.246	-1.197	-1.555	-1.753	-1.007	-1.410	1.109	1.026
1022	RPPos	TG(56:2)	9.45	-2.299	-1.476	-1.559	-1.999	-1.991	-1.251	-2.389	-3.626	-2.041
1023	RPPos	TG(56:3)>TG(18:1_18:1_20:1) and TG(18:1_18:2_20:0) and TG(18:0_18:2_20:1) and TG(16:0_18:1_22:2)	9.25	-2.075	-1.441	-1.518	-1.840	-1.762	-1.159	-2.501	-3.010	-1.831
1024	RPPos	TG(56:3)>TG(18:1_18:1_20:1) and TG(18:1_18:2_20:0) and TG(18:0_18:2_20:1) and TG(16:0_18:1_22:2)	8.95	-1.520	-1.834	-1.513	-1.546	-2.858	-1.375	-1.693	-2.533	-1.997
1025	RPPos	TG(56:4)>TG(18:0_18:0_20:4)	9.17	-1.659	-1.134	-1.235	-2.262	-1.397	-1.146	-2.105	1.115	1.064
1026	RPPos	TG(56:4)>TG(18:1_18:2_20:1) and TG(18:1_18:1_20:2)	8.85	-1.747	-1.512	-1.560	-1.477	-2.039	-1.212	-2.025	-3.381	-2.056
1027	RPPos	TG(56:4)>TG(18:1_18:2_20:1) and TG(18:1_18:1_20:2)	8.54	-1.435	-1.348	-1.169	-1.058	-1.633	-1.128	-1.567	-2.146	-1.988
1028	RPPos	TG(56:4)>TG(18:1_18:2_20:1) and TG(18:1_18:1_20:2)	8.75	-1.339	-1.398	-1.243	-1.208	-1.449	-1.036	-1.247	-1.132	-1.042
1029	RPPos	TG(56:5)>TG(16:0_18:1_22:4)	8.63	-1.094	-1.010	-1.054	-1.067	-1.610	-1.155	1.040	-1.074	-1.504
1030	RPPos	TG(56:5)>TG(16:0_18:1_22:4)	8.75	-1.404	-1.372	-1.252	-1.134	-1.287	1.033	-1.185	-1.150	1.029
1031	RPPos	TG(56:6)>TG(18:0_16:0_22:6)	8.61	-1.846	-1.623	-1.556	-1.546	-1.428	1.147	-1.549	-1.145	-1.737
1032	RPPos	TG(56:6)>TG(18:1_18:1_20:4) and TG(16:0_18:1_22:5) and TG(18:1_18:2_20:3)	8.36	-1.147	1.090	1.023	1.096	-1.452	-1.013	-1.255	-1.066	-1.394
1033	RPPos	TG(56:7)	8.07	1.040	-1.003	-1.062	-1.020	-1.442	1.094	-1.088	1.092	-1.098
1034	RPPos	TG(56:7)	7.81	-1.144	1.052	-1.129	-1.097	-1.100	-1.149	-1.275	1.348	-1.102
1035	RPPos	TG(56:7)>TG(16:0_18:1_22:6)	8.23	-1.038	1.026	1.088	-1.130	-1.647	1.113	-1.108	-1.179	-1.421
1036	RPPos	TG(56:8)	7.80	-1.137	-1.026	-1.242	-1.123	-1.384	-1.222	-1.363	1.117	-1.289
1037	RPPos	TG(56:8)	7.94	1.148	1.113	1.167	-1.081	-1.378	1.319	-1.086	1.036	-1.111
1038	RPPos	TG(56:8)	7.94	1.172	1.114	1.171	-1.073	-1.362	1.329	-1.079	1.043	-1.107
1039	RPPos	TG(56:9)	7.62	-1.235	-1.076	-1.058	-1.174	-1.540	1.039	-1.558	-1.129	-1.886
1040	RPPos	TG(57:1)	9.70	-1.874	1.105	-1.058	-1.624	-1.295	1.194	-1.689	-2.174	-1.170
1041	RPPos	TG(57:1)	9.22	-1.621	-1.715	-1.539	-1.744	-2.076	-1.237	-1.445	-1.778	-1.603
1042	RPPos	TG(57:2)	9.55	-2.470	-1.397	-1.471	-1.851	-1.680	1.043	-1.925	-3.121	-1.438
1043	RPPos	TG(57:2)	8.81	-1.112	-1.324	-1.174	-1.024	-1.889	-1.210	-1.120	-1.456	-1.596
1044	RPPos	TG(57:2)	8.92	-1.905	-1.280	-1.137	-1.784	-1.583	-1.421	-1.721	-1.159	-2.286
1045	RPPos	TG(57:3)	9.39	-2.465	-1.349	-1.423	-2.069	-1.896	-1.023	-2.474	-3.980	-1.845
1046	RPPos	TG(57:3)	8.80	-1.598	-1.148	-1.322	-1.393	-1.203	-1.000	-1.345	-1.279	-1.075

1047	RPPos	TG(57:3)	8.50	1.122	-1.061	1.016	1.049	-1.201	-1.027	1.022	-1.011	-1.038
1048	RPPos	TG(57:4)	8.18	1.025	-1.260	-1.118	-1.008	-1.685	-1.205	-1.144	-1.212	-1.369
1049	RPPos	TG(57:4)	9.07	-2.252	-1.657	-1.502	-1.754	-2.474	-1.095	-3.361	-5.343	-2.750
1050	RPPos	TG(57:4)	9.32	-2.897	-1.194	-1.316	-1.837	-2.793	1.352	-2.584	-1.847	1.013
1051	RPPos	TG(57:5)	8.75	-2.221	-1.530	-1.304	-1.719	-1.728	-1.689	-2.209	-2.596	-2.094
1052	RPPos	TG(57:5)	8.96	-1.704	-1.320	-1.036	-1.323	1.044	1.112	-1.301	-1.341	-1.390
1053	RPPos	TG(57:6)	8.56	-1.212	-1.276	-1.023	-1.065	-1.371	1.124	-1.223	-1.254	-1.261
1054	RPPos	TG(57:6)	8.29	-1.395	-1.529	-1.294	-1.290	-1.529	-1.021	-1.596	-1.743	-1.780
1055	RPPos	TG(57:7)	8.40	-1.185	-1.049	1.042	-1.265	-1.802	1.228	-1.251	-1.560	-1.167
1056	RPPos	TG(57:7)	7.53	-2.169	-2.219	-1.410	-1.867	-2.585	-1.605	-2.388	-2.355	-3.647
1057	RPPos	TG(57:8)	7.94	1.029	1.077	1.174	1.048	-1.170	1.460	-1.429	-1.255	-1.367
1058	RPPos	TG(57:8)	8.08	-1.119	-1.473	1.075	-1.404	-1.631	1.331	-1.069	-1.416	-1.261
1059	RPPos	TG(58:1)	9.77	-2.020	-1.063	-1.107	-1.847	-1.299	1.077	-1.886	-3.171	-1.117
1060	RPPos	TG(58:1)	9.38	-1.400	-1.244	-1.385	-2.036	-1.404	-1.030	-1.397	-1.500	-1.516
1061	RPPos	TG(58:1)	9.23	-1.530	-1.518	-1.408	-1.669	-1.852	-1.117	-1.403	-1.691	-1.380
1062	RPPos	TG(58:10)	7.70	1.066	1.275	1.027	1.078	1.457	1.237	-1.053	2.304	1.278
1063	RPPos	TG(58:10)	7.62	-2.324	-1.253	-1.442	-2.053	1.040	1.335	-2.048	1.472	1.193
1064	RPPos	TG(58:11)	7.49	1.119	1.450	1.269	1.164	2.176	1.442	1.132	4.473	1.538
1065	RPPos	TG(58:11)	7.49	1.119	1.450	1.269	1.165	2.176	1.442	1.130	4.490	1.541
1066	RPPos	TG(58:2)	9.61	-2.548	-1.294	-1.194	-1.958	-1.591	-1.012	-2.817	-4.414	-1.964
1067	RPPos	TG(58:3)	9.49	-2.635	-1.478	-1.498	-1.895	-1.693	-1.047	-2.657	-4.402	-2.186
1068	RPPos	TG(58:3)>TG(18:1_20:1_20:1) and TG(18:1_18:1_22:1)	9.35	-2.541	-1.473	-1.357	-1.374	-1.803	-1.070	-2.624	-3.211	-1.761
1069	RPPos	TG(58:4)	9.24	-1.913	-1.369	-1.302	-2.114	-1.842	-1.070	-2.316	-4.727	-2.476
1070	RPPos	TG(58:5)	8.94	-1.744	-1.356	-1.457	-1.153	-2.017	-1.367	-2.121	-3.741	-1.826
1071	RPPos	TG(58:6)	8.43	-1.634	-1.223	-1.007	1.059	-2.527	-1.061	-1.586	-2.019	-1.053
1072	RPPos	TG(58:7)>TG(18:0_18:1_22:6)	8.60	-1.486	-1.320	-1.283	-1.401	-1.501	1.038	-1.548	-1.447	-1.255
1073	RPPos	TG(58:8)	8.07	1.058	-1.044	-1.028	-1.009	-2.010	-1.022	1.029	1.083	1.047
1074	RPPos	TG(58:8)>TG(18:0_20:4_20:4) and TG(58:8)>TG(18:0_18:2_22:6)	8.24	1.186	-1.122	-1.046	1.026	-1.468	1.029	-1.178	-1.058	-1.185
1075	RPPos	TG(58:9)	7.94	-1.032	1.015	-1.103	1.095	1.041	1.061	-1.074	1.303	1.069
1076	RPPos	TG(58:9)	7.60	1.087	1.910	1.363	1.128	2.309	1.658	1.153	3.989	2.540
1077	RPPos	TG(58:9)	7.61	-1.577	-1.219	-1.284	-1.095	-1.004	-1.124	-1.136	-1.032	-1.142
1078	RPPos	TG(59:5)	9.42	-2.525	-1.781	-1.566	-2.032	-1.827	1.022	-2.235	-2.839	-1.713
1079	RPPos	TG(59:5)	8.76	-1.822	-1.021	-1.563	-1.748	-1.396	1.163	-1.366	1.572	1.126
1080	RPPos	TG(59:5)	8.56	-1.728	-1.892	-1.615	-1.323	-2.832	-1.075	-1.790	-1.591	-2.036
1081	RPPos	TG(60:1)	9.91	-1.798	-1.037	-1.045	-1.771	-1.114	1.087	-1.683	-3.452	1.019

1082	RPPos	TG(60:10)	8.18	1.091	1.333	1.076	1.227	1.380	1.467	-1.007	1.354	1.441
1083	RPPos	TG(60:10)	7.69	1.020	1.577	1.571	1.098	1.150	1.657	1.143	1.828	2.579
1084	RPPos	TG(60:11)	7.50	1.212	1.408	1.353	1.222	2.202	1.525	1.183	3.857	1.512
1085	RPPos	TG(60:11)	7.75	1.151	1.457	1.168	1.341	1.327	1.382	1.236	3.060	1.613
1086	RPPos	TG(60:11)	7.70	1.302	1.724	1.522	1.408	2.027	1.271	1.237	2.295	1.561
1087	RPPos	TG(60:11)	7.84	1.062	1.724	1.075	1.093	1.613	1.368	1.078	2.584	1.694
1088	RPPos	TG(60:12)	7.60	1.101	1.973	1.404	1.154	2.394	1.696	1.217	4.338	2.629
1089	RPPos	TG(60:12)	7.73	-1.228	1.130	-1.030	-1.197	-1.691	1.114	-1.365	1.060	-1.030
1090	RPPos	TG(60:2)	9.21	-1.750	-1.924	-1.681	-1.834	-3.029	-1.403	-1.672	-4.006	-2.063
1091	RPPos	TG(60:2)	9.75	-2.591	-1.294	-1.292	-1.983	-1.396	1.034	-2.118	-3.926	-1.590
1092	RPPos	TG(60:3)	9.63	-2.625	-1.330	-1.295	-1.807	-1.554	-1.022	-2.483	-4.279	-1.997
1093	RPPos	TG(60:3)	9.52	-3.113	-1.383	-1.655	-1.826	-1.581	-1.089	-2.962	-4.788	-2.046
1094	RPPos	TG(60:4)	9.48	-2.711	-1.607	-1.680	-1.790	-1.495	-1.000	-2.590	-5.769	-2.214
1095	RPPos	TG(60:4)	8.76	-1.361	-1.197	-1.075	-1.413	-1.522	-1.101	-1.615	-1.110	-1.212
1096	RPPos	TG(60:5)	9.30	-2.847	-1.570	-1.566	-2.280	-1.857	-1.024	-3.126	-5.743	-1.975
1097	RPPos	TG(60:5)	9.42	-2.598	1.021	-1.219	-1.556	-1.389	1.076	-2.003	-2.819	1.047
1098	RPPos	TG(60:6)	9.01	-2.108	-1.520	-1.530	-1.516	-1.454	1.076	-1.734	-3.189	-2.547
1099	RPPos	TG(60:6)	9.37	-3.023	-1.274	-1.297	-1.755	-1.284	1.144	-1.691	-1.554	-1.060
1100	RPPos	TG(60:6)	9.25	-2.193	-1.564	-1.339	-1.617	-1.866	-1.091	-2.309	-3.363	-1.693
1101	RPPos	TG(60:7)	7.84	1.220	1.005	1.170	1.086	-1.347	1.115	-1.084	1.087	-1.474
1102	RPPos	TG(60:7)	8.06	-1.032	1.029	1.069	-1.119	-1.395	1.325	-1.651	-1.225	-2.161
1103	RPPos	TG(60:7)	8.70	1.001	1.011	-1.096	-1.041	-1.591	1.070	-1.434	-1.635	-1.137
1104	RPPos	TG(60:8)	8.41	1.139	-1.019	1.019	1.225	-1.287	1.013	-1.007	-1.318	-1.362
1105	RPPos	TG(60:8)	8.57	-1.291	-1.125	-1.093	-1.008	-1.425	1.168	-1.159	-1.545	-1.204
1106	RPPos	TG(60:9)	8.27	1.012	-1.026	-1.093	1.136	-1.164	1.092	-1.062	1.097	1.070
1107	RPPos	TG(61:3)	9.23	-2.217	-1.597	-1.326	-2.016	-2.296	-1.283	-2.157	-3.967	-2.557
1108	RPPos	TG(61:3)	9.70	-2.762	-1.475	-1.497	-1.897	-1.463	1.115	-2.244	-4.084	-1.650
1109	RPPos	TG(61:4)	8.94	-1.510	-1.582	-1.548	-1.794	-2.407	-1.216	-2.021	-2.329	-2.528
1110	RPPos	TG(61:4)	8.84	-1.149	-1.022	1.141	-1.169	-1.283	-1.164	-1.688	-2.341	-1.320
1111	RPPos	TG(61:4)	9.58	-2.415	-1.450	-1.506	-1.726	-1.562	1.090	-1.946	-3.691	-1.393
1112	RPPos	TG(62:11)	8.20	-1.567	1.501	1.332	-1.129	-1.370	2.204	1.663	1.862	3.260
1113	RPPos	TG(62:2)	9.89	-2.438	-1.151	-1.232	-1.755	-1.096	1.082	-1.768	-6.586	-1.254
1114	RPPos	TG(62:3)	9.75	-3.289	-1.528	-1.413	-2.309	-1.518	1.078	-1.937	-5.066	-1.446
1115	RPPos	TG(62:3)	9.40	-2.042	-1.184	-1.345	-2.614	-1.923	-1.128	-3.832	-2.372	-1.929
1116	RPPos	TG(62:3)	9.25	-1.497	-1.142	-2.520	-1.059	-1.850	1.019	-2.539	-2.618	-1.343
1117	RPPos	TG(62:4)	9.62	-3.218	-1.724	-1.483	-1.954	-1.391	1.020	-2.544	-5.138	-1.835
1118	RPPos	TG(62:4)	8.95	-1.648	-1.428	-1.650	-1.491	-1.958	-1.492	-1.913	-2.534	-3.020

1119	RPPos	TG(62:5)	9.58	-2.247	-1.333	-1.313	-1.323	-1.167	1.135	-1.692	-2.822	1.038
1120	RPPos	TG(62:5)	9.51	-2.569	-1.446	-1.427	-1.650	-1.462	-1.035	-2.816	-5.977	-2.297
1121	RPPos	TG(62:6)	9.54	-2.570	-1.031	-1.282	-1.153	-1.047	1.308	-2.202	-2.398	-1.031
1122	RPPos	TG(62:6)	8.36	1.076	-1.031	1.040	-1.093	-1.512	-1.144	-1.129	-1.177	-1.476
1123	RPPos	TG(62:6)	9.41	-2.460	-1.486	-1.384	-1.306	-1.061	1.211	-2.721	-3.163	-1.902
1124	RPPos	TG(62:6)	8.61	-1.448	-1.051	-1.093	-1.521	-1.846	-1.035	-1.878	-1.246	-1.496
1125	RPPos	TG(64:4)	9.75	-3.459	-1.888	-1.127	-2.007	-1.314	1.135	-2.227	-8.648	-1.333
1126	RPPos	TG(64:6)	9.02	-2.186	-1.122	-1.424	-1.772	-1.123	1.125	-2.412	1.283	1.085
